# A randomized, double‐blind phase 1b study evaluating the safety, tolerability, pharmacokinetics and pharmacodynamics of the NLRP3 inhibitor selnoflast in patients with moderate to severe active ulcerative colitis

**DOI:** 10.1002/ctm2.1471

**Published:** 2023-11-14

**Authors:** Barbara Klughammer, Luca Piali, Alexandra Nica, Sandra Nagel, Lorna Bailey, Christoph Jochum, Stanislav Ignatenko, Angela Bläuer, Sabrina Danilin, Pratiksha Gulati, Joanne Hayward, Petar Scepanovic, Jitao David Zhang, Satish Bhosale, Chui Fung Chong, Andreas Christ

**Affiliations:** ^1^ F. Hoffmann‐La Roche AG Basel Switzerland; ^2^ Roche Products Limited Welwyn Garden City UK; ^3^ Charité ‐ Universitätsmedizin Berlin Berlin Germany; ^4^ Charité Research Organisation GmbH Berlin Germany; ^5^ A4P Bio Sandwich UK; ^6^ IQVIA RDS (India) Pvt Ltd Thane India

**Keywords:** biomarker, inflammatory bowel disease, interleukin‐1β, NLRP3 inflammasome, NLRP3 inhibitor, pharmacokinetics, phase 1b, safety, ulcerative colitis

## Abstract

**Background:**

The NLRP3 inflammasome drives release of pro‐inflammatory cytokines including interleukin (IL)‐1β and IL‐18 and is a potential target for ulcerative colitis (UC). Selnoflast (RO7486967) is an orally active, potent, selective and reversible small molecule NLRP3 inhibitor. We conducted a randomized, placebo‐controlled Phase 1b study to assess the safety, tolerability, pharmacokinetics (PK) and pharmacodynamics (PD) of selnoflast.

**Methods:**

Nineteen adults with previous diagnosis of UC and current active moderate to severe disease were randomized 2:1 to selnoflast or placebo for 7 days. A dose of 450 mg QD (once daily) was selected to achieve 90% IL‐1β inhibition in plasma and colon tissue. Consecutive blood, sigmoid colon biopsies and stool samples were analyzed for a variety of PD markers. Safety and PK were also evaluated.

**Results:**

Selnoflast was well‐tolerated. Plasma concentrations increased rapidly after oral administration, reaching T_max_ 1 h post‐dose. Mean plasma concentrations stayed above the IL‐1β IC_90_ level throughout the dosing interval (mean C_trough_ on Day 1 and Day 5: 2.55 μg/mL and 2.66 μg/mL, respectively). At steady state, post‐dose selnoflast concentrations in sigmoid colon (5‐20 μg/g) were above the IC_90_. Production of IL‐1β was reduced in whole blood following ex vivo stimulation with lipopolysaccharide (LPS) (in the selnoflast arm). No changes were observed in plasma IL‐18 levels. There were no meaningful differences in the expression of an IL‐1‐related gene signature in sigmoid colon tissue, and no differences in the expression of stool biomarkers.

**Conclusions:**

Selnoflast was safe and well‐tolerated. Selnoflast 450 mg QD achieved plasma and tissue exposure predicted to maintain IL‐1β IC_90_ over the dosing interval. However, PD biomarker results showed no robust differences between treatment arms, suggesting no major therapeutic effects are to be expected in UC. The limitations of this study are its small sample size and indirect assessment of the effect on IL‐1β in tissue.

**Trial registration:**

ISRCTN16847938

## Graphical abstract

Although selnoflast was well‐tolerated and reached sufficient plasma and tissue exposure to inhibit the NLRP3 inflammasome, there were no meaningful changes in sigmoid colon histology or markers of inflammation. No major therapeutic effect is expected in ulcerative colitis.

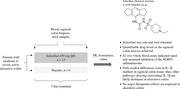

## 1 INTRODUCTION

As a major subtype of inflammatory bowel disease (IBD), ulcerative colitis (UC) is a chronic, debilitating, and progressive autoimmune disorder characterized by relapsing and remitting mucosal inflammation which extends from the rectum up to proximal segments of the colon.^1^ The unpredictable clinical course is reflected by periods of exacerbation and remission which undermine health‐related quality of life leading to a greater personal and socioeconomical burden.^2^, ^3^ The incidence and prevalence of UC are increasing worldwide, suggesting an involvement of environmental factors alongside genetic predisposition, immunity and the gut microbiome in the etiology of this complex disorder.^4^

Nearly half of patients with UC require hospitalization at some point, and of these 50% have a 5‐year risk of re‐hospitalization.^5^ There is no cure: the goal of treatment is to achieve durable symptomatic and endoscopic remission without the need for corticosteroids, and to prevent colectomy and colorectal cancer.^1^, ^6^ Current non‐surgical therapies, including 5‐aminosalicylic acid (5‐ASA), glucocorticoids, immunosuppressants, and biological agents (such as anti‐TNF agents and others), are limited by insufficient efficacy evidenced by flares, side effects and a high relapse rate.^7^, ^8^, ^9^ Despite recent advances in novel therapeutic targets in IBD, the majority of patients with moderate to severe UC still lack an effective, long‐term therapy.^10^, ^11^

Inflammasomes are multimeric protein complexes that are formed in various immune and non‐immune cells in response to signals from pathogens, and are a key component of the innate immune response.^12^ An inflammasome is defined by its pattern‐recognition receptor (PRR) protein, which upon activation, oligomerizes with other proteins to form a multimeric complex that acts as a platform for the activation of caspase‐1.^13^ In turn, this leads to the maturation and release of the cytokines interleukin (IL)−1β and IL‐18 and the onset of programmed lytic cell death (pyroptosis).^12^ The nucleotide binding oligomerization domain‐like receptor family pyrin 3 (NLRP3) inflammasome is expressed in many tissues, including in the immune cells of the gut.^14^ The NLRP3 inflammasome is activated upon exposure to diverse signals, such as pathogen‐associated molecular patterns (PAMPs), danger‐associated molecular patterns (DAMPs), dead cells, and external irritants.^15^ Aberrant activation of the NLRP3 inflammasome has been implicated in a broad spectrum of inflammatory disorders including atherosclerosis, Alzheimer's disease, gastrointestinal cancers, and UC.^15^, ^16^ NLRP3 and IL‐1β are upregulated in active UC,^17^ and genomic studies have shown that polymorphisms in NLRP3‐related genes may affect individual susceptibility to IBD.^14^ Finally, inhibition of IL‐1β and IL‐18 downstream of NLRP3 inflammasome signaling ameliorates gut inflammation in several animal models of colitis.^18^, ^19^, ^20^, ^21^ However, the mechanisms that regulate inflammasome activity are complex, frequently hampering the interpretation of results and leading to observations of contradictory findings.^22^

Selnoflast (RO7486967) is a selective and reversible small molecule NLRP3 inflammasome inhibitor. In vitro pharmacological studies revealed that selnoflast is a potent inhibitor of IL‐1β release in activated human monocyte‐derived macrophages. Selnoflast has no inhibitory activity on two other inflammasomes, the Nucleotide‐binding Oligomerization Domain (NOD)‐like receptor (NLR) family CARD domain containing 4 (NLRC4) or absent in melanoma‐2 (AIM2) (unpublished results). Selnoflast has been studied in one completed Phase 1, randomized, double‐blind, placebo‐controlled Entry‐Into‐Human study (NCT04086602), which evaluated the safety, tolerability, pharmacokinetics (PK) and pharmacodynamics (PD) after single and multiple ascending doses administered orally in 64 healthy subjects.

Here, we report the findings from a randomized, placebo‐controlled, investigator‐ and patient‐blinded Phase 1b study designed to evaluate the safety, tolerability, PK, and PD of selnoflast in patients with moderate to severe active UC.

## 2 METHODS

The protocol for this study and CONSORT checklist are available as Supporting Information.

### 2.1 Study design

This was a randomized, placebo controlled, investigator‐ and patient‐blinded study to assess the safety, tolerability, PK and PD of selnoflast in patients with moderate to severe active UC. The sponsor teams and study personnel were fully blinded, with the exception of selected individuals (e.g., in the analysis laboratories), who had to be unblinded in order to identify the correct samples for PK analysis. A total of 19 patients were enrolled. All had been previously diagnosed with UC and were in an active stage of their disease during the study. Patients were randomized 2:1 to receive either 450 mg selnoflast orally or placebo. The treatment duration was 7 days (at the clinic). Patients were recruited and enrolled at one study site in Berlin, Germany, between December 2021‐June 2022. The dose of 450 mg administered once daily was selected based on the results from an entry‐in‐humans (EIH) study in healthy subjects, where it was well tolerated over 7 days of treatment and showed 99% inhibition of IL‐1β release up to 10 h post‐dose in ex vivo stimulated whole blood, and over 90% inhibition over the dosing interval, compared to baseline (unpublished results).

The study protocol was approved by the institutional review board of the participating study site and complied with the Declaration of Helsinki. All patients provided written informed consent prior to starting the study.

### 2.2 Study participants

Male and female adults (18–75 years) were enrolled in the study. All patients had been diagnosed with moderate to severe UC as defined by a partial Mayo Clinic Score (MCS) ≥3 and ≤8, with stool frequency subscore ≥1, rectal bleeding subscore ≥1, and fecal calprotectin (FC) ≥150 μg/g. Patients had to have their UC diagnosis at least 12 weeks prior to screening, and the most recent colonoscopy within the last 3 years prior to screening. Those with a history of pancolitis and disease duration of ≥8 years, and those with a history of left sided colitis and disease duration ≥12 years, had to have undergone colonoscopy screening for colorectal cancer within 2 years prior to screening.

Patients were excluded if they had fulminant UC, Crohn's disease, indeterminate colitis, microscopic colitis, segmental colitis associated with diverticulosis, ischemic colitis, radiation‐induced colitis, chronic liver disease or abnormal hepatic enzyme or liver function test values, history of colectomy or partial colectomy. Prohibited concomitant therapies included calcineurin inhibitors (e.g., tacrolimus, cyclosporine), vedolizumab, ustekinumab, anti‐TNFα therapeutics or any other immune system‐targeted therapy within 12 weeks or 5 half‐lives (whichever was longer) prior to screening, rectal therapy with 5‐ASA or corticosteroids within 2 weeks of screening, and leukocyte apheresis within 12 weeks of screening.

### 2.3 Study treatment and randomization

Selnoflast (450 mg QD or matching placebo) was administered orally in the morning following an overnight fast of at least 8 h during the patients’ 7‐day clinic stay. Water intake was restricted for 1 h pre‐dose and 1 h post‐dose, and no food was allowed until at least 4 h post‐dose.

### 2.4 Study assessments and endpoints

The primary objective of this study was to evaluate the PK, safety, and tolerability of selnoflast. The main endpoints were the incidence and severity of adverse events (AEs), changes in vital signs, 12‐lead electrocardiogram (ECG) parameters, and clinical laboratory safety parameters. AEs were monitored throughout the study and at the 14‐day follow‐up visit. Safety assessments included recording of the incidence and type of treatment‐emergent adverse events (TEAEs), discontinuations, serious TEAEs, and adverse events of special interest (AESIs) (including cases of elevated alanine aminotransferase [ALT] or aspartate aminotransferase [AST] in combination with either elevated bilirubin or clinical jaundice). Vital signs, ECGs, blood chemistry and hematology panels, coagulation panels and urinalysis were assessed at screening and at various time points during the study. Changes in absolute lymphocyte count were also assessed. Blood samples for PK analyses were obtained pre‐dose and at various timepoints up to 24 h post‐dose throughout the 7‐day in‐clinic treatment period, as well as once during the 14‐day follow‐up visit. As the 1‐week duration of the study was too short for assessing clinical changes, no clinical assessments were planned. Further details are given in the study protocol (Supporting Information).

### 2.5 Pharmacokinetics

The concentrations of selnoflast were determined in a total of 260 human plasma samples (obtained during the study) using a validated liquid chromatography‐tandem mass spectrometry (LC‐MS/MS) method. Sample analysis was performed after the addition of the internal standard by protein precipitation followed by LC‐MS/MS. The calibration range was between 40.0 ng/mL and 40000.0 ng/mL; the lower limit of quantification in human plasma was 40.0 ng/mL. Study samples were analyzed within the validated frozen storage stability timeframe of 371 days at −20°C and −70°C, the five validated freeze/thaw cycles, and proven room temperature storage stability of 24 h, in a total of six analytical runs. Five out of six runs met the acceptance criteria. The precision and accuracy (expressed as %RE) of the assay, as determined from the analysis of quality control samples, was satisfactory throughout the study.

### 2.6 Pharmacodynamics and gene expression analysis

Tissue biopsies from the sigmoid colon were obtained before start of treatment (up to Day −3) and at the end of treatment (Day 7). For operational reasons, biopsies were taken 2−4.5 h post‐dose on Day 7. Stool samples were obtained at screening, before start of treatment, throughout the study, and at the 14‐day follow‐up visit. Levels of calprotectin were determined in stool samples. Sigmoid colon tissue was processed for RNA and DNA extraction as well as for single‐cell analysis, subsequent transcriptomic analysis, and identification of PD biomarkers. Sigmoid colon tissue samples were also processed for histology (hematoxylin and eosin staining, immunohistochemistry, fluorescence in situ hybridization) in order to characterize the cell types present (i.e., neutrophils and inflammatory monocytes). Histological analysis was performed on sigmoid colon tissue samples (from baseline and Day 7) using the disease‐specific histological indices Geboes score^23^ and Nancy index.^24^ Caspase‐1 (pro and active form) was assessed using a validated WES‐assay (manuscript in preparation).

A predefined IL‐1 gene signature comprising eight genes (i.e., CXCL1, CXCL2, CXCL3, CXCL5, CXCL6, CCL2, IL6, TNFAIP6) relevant in the context of IBD was assessed in RNA extracted from the sigmoid colon tissue. The signature had been determined by combining gene expression data that met the following criteria: (1) overlapping expression profiles (upregulation by IL‐1β stimulation) in multiple cell types,^25^ (2) differential expression in relevant cell types across published and proprietary single‐cell RNA‐sequencing (scRNA‐seq) datasets involving IBD patients and controls^26^ and (3) co‐expression with the IL1R1 receptor and differential colonic gene expression between UC patients and healthy individuals. The 8‐gene panel was then assessed by quantitative PCR (qPCR) before and after treatment (baseline and Day 7), normalized to a set of housekeeping genes (B2M, GusB, HPRT1, MRLP19) and log_2_ fold changes (log_2_FC) from baseline were reported per gene as well as across the eight genes (median log_2_FC = gene signature).

Blood samples were drawn pre‐dose and at various timepoints and placed in TruCulture whole blood collection and culture tubes that were pre‐filled with lipopolysaccharide (LPS) (final working concentration of 100 ng/mL LPS after addition of 1 mL of blood to the LPS‐containing Truculture tubes). Following 24 h incubation, TruCulture supernatants were collected, stored, and levels of secreted IL‐1β were subsequently measured in order to understand how these responded to NLRP3 inhibition by selnoflast. The levels of IL‐1β were also quantified in plasma K_2_EDTA samples using the same SIMOA IL‐1β Assay Kit (Quanterix). The quantitative range of the method was 0.196 to 490 pg/mL for plasma and 4.01 to 1470 pg/mL in Truculture supernatants. Plasma samples were also assessed for IL‐18, using a commercial SIMOA IL‐18 Assay Kit (Quanterix; the quantitative range for plasma was 0.0200–45.0 pg/mL).

### 2.7 Single‐cell RNA‐sequencing and data analysis

Single cell suspensions were obtained from cryopreserved sigmoid colon tissue samples through mechanical and enzymatic dissociation, and loaded onto a 10X chromium controller for cell partitioning. Single‐cell RNA‐sequencing libraries were prepared using 10X genomics NextGEM single‐cell 3′ version 3.1 reagents and sequenced on an Illumina NovaSeq 6000 sequencer. Quality control, data filtering, and data analysis was performed with the BESCA software (version 2.5.1)^27^ and the RiBIOS software suite, as well as using custom scripts in both R (version 4.2.0) and Python (version 3.8).

### 2.8 Statistical analysis

All randomized patients were included in the intent‑to‑treat population. All patients completed all doses of study treatment and all were included in the safety, PK and PD analyses. Safety data were grouped according to treatment received and were tabulated and/or listed, as appropriate. PK parameters (including T_max_, C_max_, C_trough_, total exposure, drug accumulation, clearance, volume of distribution and T_1/2_) were extrapolated directly from the plasma concentration‐versus‐time profiles or calculated using standard non‐compartmental methods. PK and PD parameters were summarized and presented descriptively.

## 3 RESULTS

### 3.1 Study population

**FIGURE 1:**
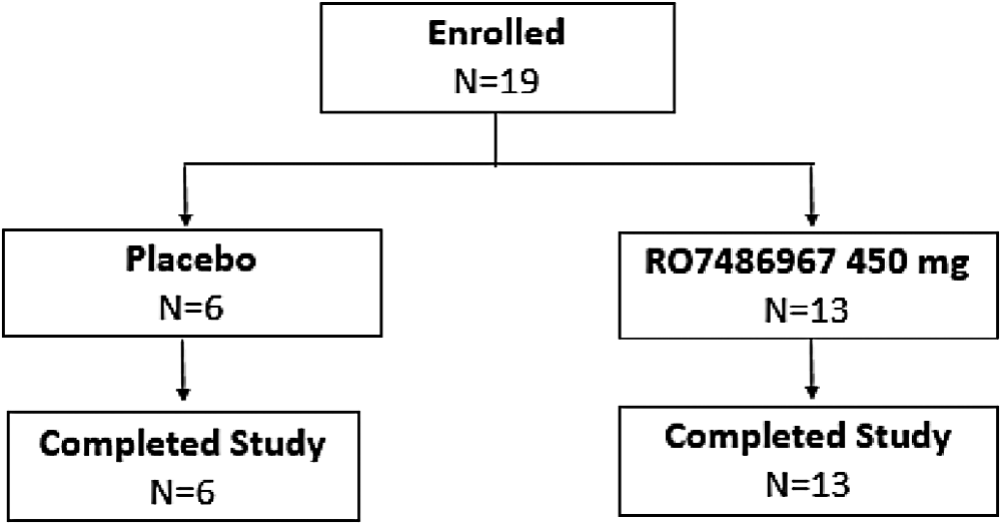
Flow chart of patient disposition.

Nineteen patients were randomized at one study site in Germany. Thirteen received selnoflast and six received placebo. All patients completed the study (patient disposition is shown in Figure 1).

The mean (± standard deviation [SD]) age was higher in the selnoflast arm (38.8 [± 13.7] years) compared to the placebo arm (29.4 [± 6.3] years). There was a higher proportion of males in the placebo arm (5 [83.3%] compared to 5 [38.5%] in the selnoflast arm). On average, the weight was also higher in the placebo group. Otherwise, the baseline characteristics of the patients were similar in both arms (Table 1).

**TABLE 1:** Baseline characteristics of the study population.

	Placebo (*N* = 6)	Selnoflast, 450 mg (*N* = 13)
Age (years)		
Mean (SD)	29.4 (6.3)	38.8 (13.7)
Median (Min—Max)	27.2 (23.8–40.8)	35.7 (19.9–64.6)
Sex (*n* [%])		
Male	5 (83.3%)	5 (38.5%)
Female	1 (16.7%)	8 (61.5%)
Race (*n* [%])		
White	6 (100%)	13 (100%)
Weight (kg)		
Mean (SD)	86.0 (9.6)	71.4 (13.7)
Median (Min—Max)	85.3 (70.0–97.1)	71.5 (49.9–93.8)
Partial Mayo Clinic Score		
Mean (SD)	5.5 (1.5)	5.9 (1.0)
Median (Min—Max)	5.5 (4.0–8.0)	6 (4.0–8.0)
Stool calprotectin (μg/g)		
Mean (SD)	5673.2 (11 911.6)	1581.0 (1932.0)
Median (Min—Max)	667.6 (99.0–29947.0)	694.5 (59.0–6284.8)
Prior anti‐TNF therapy (n [%])		
Infliximab or biosimilar	0	1 (7.7%)
Golimumab	0	1 (7.7%)
Prior immunosuppressive therapy (n [%])		
Vedolizumab	0	2 (15.4%)
Risankizumab	1 (16.7%)	0
Azathioprine	2 (33.3%)	1 (7.7%)

### 3.2 Prior and concomitant medications

Overall, 26.3% (five patients, [two patients on placebo and three patients on selnoflast]) had at least one immunosuppressive therapy prior to participating in the study (azathioprine: three patients, risankizumab: one patient, and vedolizumab: two patients). One of the patients with prior vedolizumab treatment also had prior anti‐TNF medication (infliximab and golimumab).

Twelve patients (63.1%) took mesalazine before and during the study. A total of 18 patients (94.7%) had been vaccinated against COVID‑19 at baseline. After the start of the treatment period, three patients (15.8%) received concomitant medications (beclometasone dipropionate; formoterol fumarate, and nadroparin calcium).

### 3.3 Exposure

All patients in both study arms received all seven doses of study treatment. The total cumulative dose was 3150 mg per patient in the selnoflast arm.

### 3.4 Safety

A total of 13 AEs was reported in 11 patients, 12 of which were reported by 10 patients in the selnoflast arm. All AEs were mild (Grade 1), with the exception of one AE of Grade 2 severity (asthma; unrelated to the study drug). The most frequently reported AEs (by Preferred Term [PT]) in the selnoflast arm were headache and dyspepsia (two patients each [15.4%]); all other AEs were reported once in individual patients. One patient in the placebo arm experienced a single AE of headache. Three AEs (23.1%) in the selnoflast arm (dyspepsia [two patients] and headache [one patient]) and one AE (16.7%) in the placebo arm (headache) were considered treatment‐related. No AEs led to treatment discontinuation or dose interruption. There were no serious AEs or deaths during the study. There were no clinically meaningful changes in clinical chemistry (including liver function tests), urinalysis, vital signs, ECGs, or other safety parameters. A summary of the safety findings is provided in Table 2.

**TABLE 2:** Summary of safety results in patients with ulcerative colitis.

	Placebo (*N* = 6)	Selnoflast (*N* = 13)	All patients (*N* = 19)
Patients with at least one adverse event (AE)	1 (16.7%)	10 (76.9%)	11 (57.9%)
Total AEs	1	12	13
Deaths	0	0	0
Patients withdrawn from study due to AE	0	0	0
Patients with at least one			
AE with fatal outcome	0	0	0
Serious AE	0	0	0
AE leading to withdrawal from treatment	0	0	0
AE leading to dose modification/interruption	0	0	0
Related AE	1 (16.7%)	3 (23.1%)	4 (21.1%)
Related AE leading to withdrawal from treatment	0	0	0
Related AE leading to dose modification/ interruption	0	0	0

### 3.5 Pharmacokinetics

**FIGURE 2:**
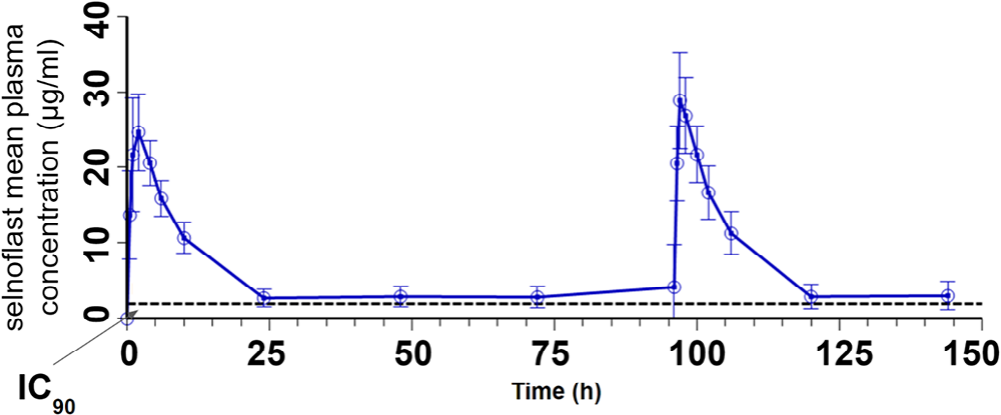
Selnoflast plasma concentrations (mean ± SD) versus time. Treatment showed a rapid increase and limited accumulation over 7 days. Steady state was achieved on Day 2. The inter‐patient variability was low to moderate. Mean plasma concentrations remained above the IC_90_ at trough.

**FIGURE 3:**
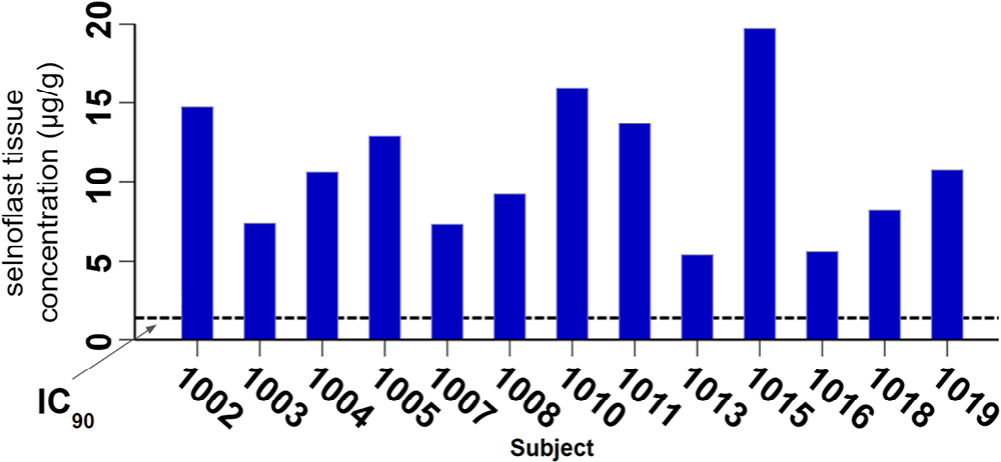
Concentration of selnoflast in sigmoid colon mucosal tissue. Selnoflast concentrations were measured in sigmoid colon biopsies from individual patients, taken 2–4.5 h post‐dose on Day 7. The dotted line represents the tissue IC_90_ value.

The mean plasma concentrations of selnoflast over the treatment period are depicted in Figure 2 and the main PK parameters are summarized in Table 3. There was a rapid increase in plasma concentrations of selnoflast after oral administration following an overnight fast of at least 8 h. T_max_ (median) was reached at 1 h at steady‐state. Steady‐state was achieved on Day 2 of treatment. After repeat dosing, there was a low accumulation in terms of maximum plasma concentration (C_max_), area under the concentration‐time curve over the dosing interval (AUC_tau_) and C_last_, which increased by 17%, 8.6% and 4.3%, respectively, from Day 1 to Day 5. Clearance and the terminal half‐life on Day 5 were similar to those observed after the first dose on Day 1. Inter‐patient variability remained low to moderate, with a coefficient of variation (CV%) of less than 40% except for trough plasma concentrations (C_trough_) (Table 3). The mean plasma C_trough_ level was above the IL‐1β IC_90_ as calculated from in vitro studies (estimated IC_90_ 2.0 μg/mL or 1.94 μg/g). Individual C_trough_ at steady‐state showed that IC_90_ was maintained over the dosing interval in 10 out of 13 patients who were administered selnoflast. Post‐dose sigmoid colon biopsies (taken at Day 7 [end of treatment]) showed concentrations of selnoflast ranging from 5−20 μg/g in all 13 treated patients; all concentrations were above the IC_90_ level (Figure 3).

**TABLE 3:** Summary of selnoflast plasma pharmacokinetic parameters at Day 1 and at steady‐state (Day 5).

Selnoflast, 450 mg (*N* = 13)								
	C_max_ (μg/mL)	T_max_ (h)	AUC_tau_ (h*μg/mL)	AUC_0‐inf_ (h*μg/ mL)	T_1/2_ (h)	Vz/F (L)	C_trough_ (μg/mL)	CL/F (L/h)
**Day 1**	25.0 (20.4)	1.97 (0.97‐2.00)	247 (17.5)	275 (20.7)	6.90 (17.6)	18.1 (16.6)	2.55 (38.8)	1.82 (17.5)
**Day 5**	29.3 (17.8)	1.00 (1.00‐2.07)	268 (21.1)	NA	6.93 (18.9)	NA	2.66 (46.6)	1.68 (21.0)

*Note*: All parameters are reported as geometric means (CV% geo means) with three significant digits (one decimal digit) except T_max_ reported as median (range) with two decimal digits, as per internal specifications for PK data reporting. C_trough_ values reported for Day 1 and Day 5 are the last concentrations measured over the dosing interval (or C_last_, collected 24 h following the last dose as per protocol).

Abbreviations: AUC_tau_  = area under curve over the dosing interval; AUC_0‐inf_  = area under curve from time 0 extrapolated to infinity; CL/F = apparent clearance; C_max_ = observed maximum plasma concentration; C_trough_ = observed trough plasma concentration; h = hour; NA = not applicable per internal non‐compartmental analysis (NCA) Guiding Principles; T_max_  = time to maximum plasma concentration; T_1/2_ =  plasma elimination half‐life; Vz/F = apparent volume of distribution after the first administration.

### 3.6 Pharmacodynamics and biomarker analyses

**FIGURE 4:**
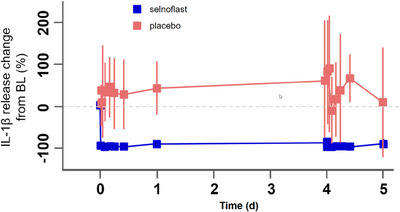
Inhibition of IL‐1β release in whole blood upon ex vivo stimulation with LPS. BL, baseline; d, days. Blood samples were obtained from patients treated with either placebo or selnoflast, at various timepoints pre‐ and post‐dosing. Blood samples were treated with LPS for 24 h prior to measurement of IL‐1β levels (in the supernatants). The level of IL‐1β is expressed as the percentage change from baseline (pre‐dose), including standard error bars.

Secreted IL‐1β was assessed in whole blood following ex vivo stimulation with LPS, to evaluate target engagement with the NLRP3 inflammasome. Data were expressed as percentage of inhibition of IL‐1β release compared to baseline (Figure 4). There was strong and rapid inhibition of IL‐1β release following the first oral administration of selnoflast on Day 1. Inhibition occurred starting from 30 min post‐dose and was maintained until at least 10 h post‐dose (mean [± SD] of > 95% [± 4.16%]). The level of inhibition decreased to a mean (SD) of 91.2% (± 3.4%) just before the next administration of selnoflast on Day 2. Overall, the level of inhibition of IL‐1β release was high at steady‐state (Day 2), with a similar level of inhibition as seen on Day 1. Levels of inhibition were maintained at around 90% at trough (25–100 h post‐dose). No inhibition of IL‐1β release was observed in whole blood obtained from placebo‐treated patients (Figure 4).

**FIGURE 5:**
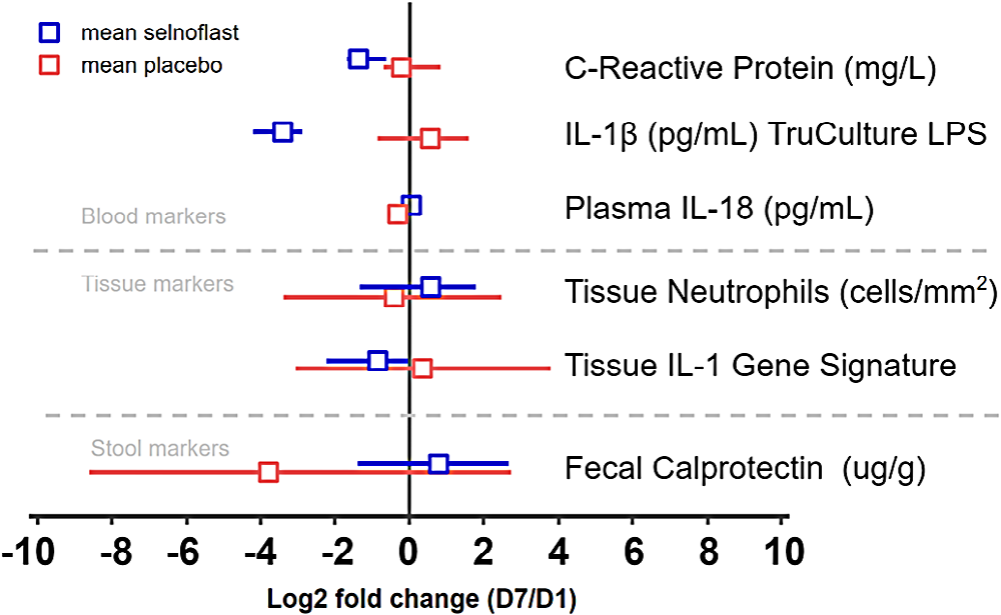
Effect of selnoflast on markers of inflammation in blood, sigmoid colon tissue and stool. The graph depicts the log_2_ fold change from baseline to Day 7 (± 95% confidence interval) in various inflammation markers measured in blood, sigmoid colon tissue and stool.

We also evaluated blood, sigmoid colon tissue and stool in order to understand the effect of selnoflast administration on disease‐relevant biomarkers (Figure 5). Blood C‐Reactive Protein (CRP) levels decreased slightly upon treatment, with a mean (± SD) change from baseline to Day 7 of ‐1.8 (± 3.65) mg/L compared to + 0.51 (± 1.43) mg/L in the placebo arm. However, this decrease was largely due to one outlier and was not considered clinically significant. Plasma levels of IL‐1β were below the limit of quantification. There were no significant changes in plasma IL‐18 levels upon treatment.

Sigmoid colon tissue biopsies were assessed histologically before and after treatment (baseline and Day 7) using the standard disease‐specific histological indices Geboes score and Nancy index. No notable differences were observed in either of the two indices between the study arms (results not shown). Three out of the 13 selnoflast‐treated patients showed a marked reduction from baseline in the level of neutrophils in sigmoid colon tissue, contributing to an overall modest mean (± SD) change from baseline of −13.23 (± 140.76) cells/mm^2^ in the active treatment arm. On average, however, there were no significant reductions in neutrophil numbers in either study arm (results not shown). Caspase‐1 (pro‐ and active forms), directly downstream of NLRP3, did not show marked changes after 7 days’ treatment with selnoflast (data not shown).

Stool calprotectin levels after treatment were higher in the selnoflast arm: on Day 7, the mean (± SD) change from baseline was +4146.6 (± 12321.12) μg/g, compared to a reduction of 7351.70 (± 14111.83) μg/g in the placebo arm. The difference was mainly driven by two individuals, one in each arm. One patient in the placebo arm showed a drop in fecal calprotectin from 29947 μg/g at baseline to 1562 μg/g on Day 7. One patient in the selnoflast arm showed an increase in fecal calprotectin from 1553 μg/g at baseline to 39770 μg/g at Day 7. It should also be noted that a substantial proportion (8/19) of the baseline stool samples were not taken pre‐biopsy as per protocol, rendering the results for this biomarker uninterpretable.

**FIGURE 6:**
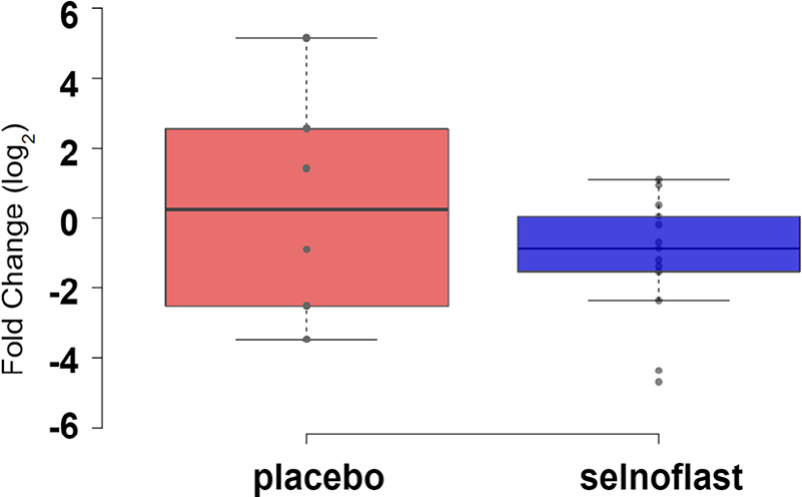
Expression of an IL‐1 gene signature in sigmoid colon tissue. RNA was extracted from sigmoid colon tissue samples that were obtained before treatment and on Day 7 to evaluate the expression of an IL‐1 gene signature by quantitative Polymerase Chain Reaction. The gene signature consisted of a panel of 8 genes: CXCL1, CXCL2, CXCL3, CXCL5, CXCL6, CCL2, IL6 and TNFAIP6. The graph depicts the log_2_ fold change from baseline to Day 7 using a Box and Whisker Plot. Minimum, lower quartile, median, upper quartile and maximum are shown. The dots represent single samples. There was a slight decrease between baseline and Day 7 in the selnoflast arm, compared to the placebo arm.

We assessed the expression of an IL‐1 gene signature (as a downstream transcriptomic marker in lieu of tissue IL‐1β where no validated assay was available to assess the active form) in sigmoid colon tissue by qPCR before and after treatment. Expression levels were normalized to a set of housekeeping genes and the log_2_ fold changes (log_2_FC) from baseline were reported per gene as well as for all 8 genes (median log_2_FC = gene signature). These 8 genes are known to respond to IL‐1β stimulation in various cell types and are differentially expressed in the colon of UC patients versus healthy individuals. The IL‐1 gene signature showed a slight decrease between baseline and the end of treatment (Day 7) in the selnoflast arm compared to placebo (log_2_FC mean [± SD] −1.14 [± 1.8] versus +0.38 [± 3.26], respectively) (Figure 6).

### 3.7 Single‐cell gene expression analysis

Single‐cell RNA sequencing was also performed to assess potential treatment‐induced changes in cellular composition as well as differential gene expression in cells isolated from the sigmoid colon.

**FIGURE 7:**
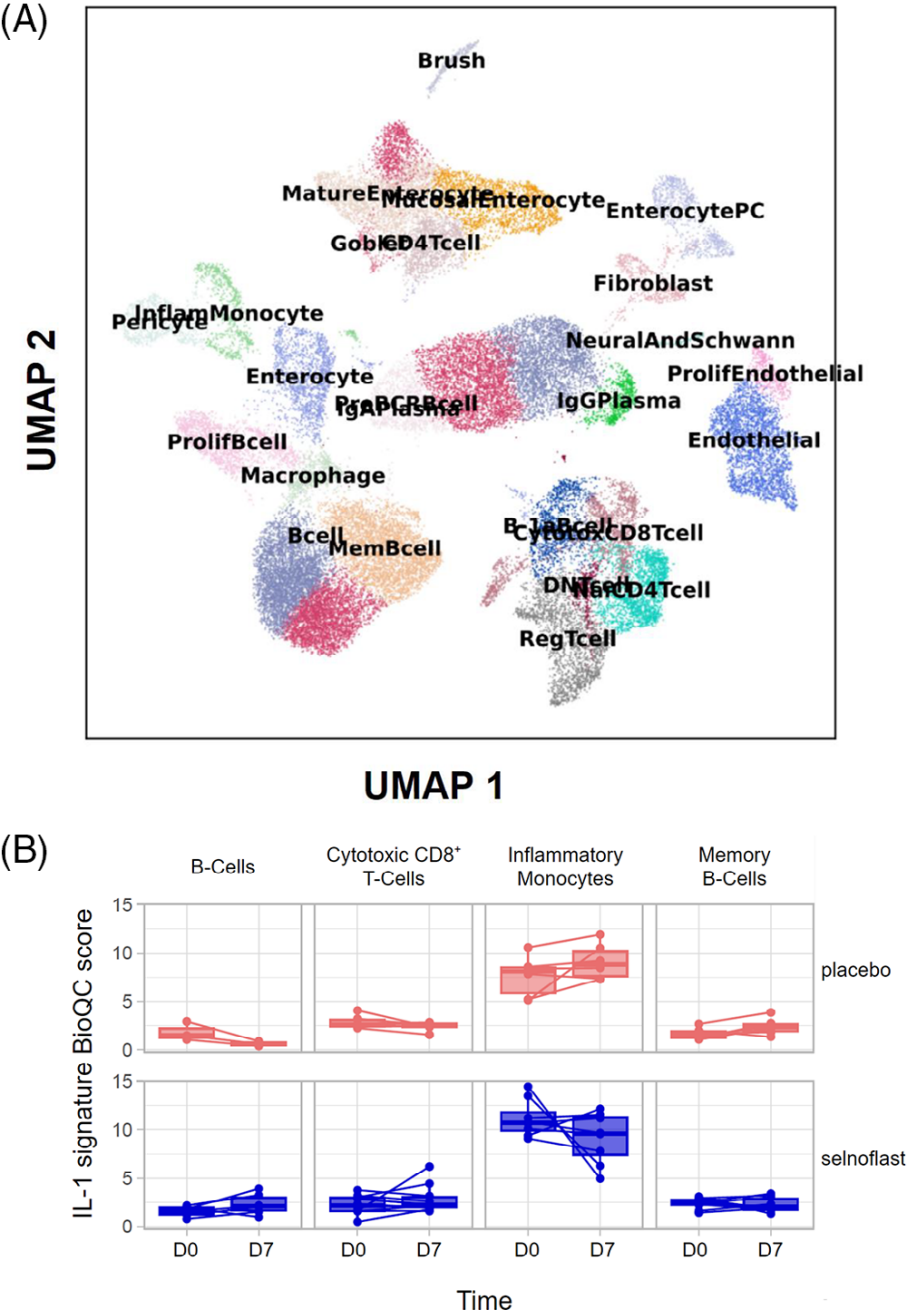
Cell types detected by single cell RNA sequencing and changed expression of IL‐1 signature genes. (A) Uniform manifold approximation and projection (UMAP) plot of single‐cell data. Each dot represents one cell. Cells with similar gene expression profiles are clustered by the Leiden algorithm implemented in the *scanpy* software package.^1^ Cell‐type annotation was performed with gene signatures provided by the *BESCA* software^2^ and with manual curation. (B) Enrichment of genes that are induced by IL‐1 (abbreviated as ‘IL‐1 signature genes’ thereafter), stratified by treatment, cell type, and time points of sampling. The IL‐1 signature genes are curated from previous studies and include well‐established targets of IL‐1 such as *IL6*, *CXCL2*, *CXCL3*, *CLCL5* and *CCL2*. Enrichment is quantified with the BioQC software.^3^ Higher BioQC scores indicate that the IL‐1 signature genes are more positively enriched in the gene expression of the sample. The plot visualizes only cell types in which the difference between placebo and treatment groups is the strongest, with unadjusted *p*‐values of Student's *t*‐test less than 0.10. ^1^Wolf FA, Angerer P, Theis FJ. SCANPY: large‐scale single‐cell gene expression data analysis. Genome Biology 2018;19(1):15. ^2^ Mädler SC, Julien‐Laferriere A, Wyss L, et al. Besca, a single‐cell transcriptomics analysis toolkit to accelerate translational research. NAR Genomics and Bioinformatics 2021;3(4):https://doi.org/10.1093/nargab/lqab102. ^3^Zhang JD, Hatje K, Sturm G, et al. Detect tissue heterogeneity in gene expression data with BioQC. BMC Genomics 2017;18(1):277.

A total of 112,392 cells were collected from all patients at two time points. After rigorous filtering, 40897 high‐quality cells were retained from both time points from all but two patients. The cells were annotated with genes preferentially expressed in colonic cell types, and almost all the expected cell types in sigmoid colon tissue were recovered (Figure 7A).

Cell‐type abundance analysis as well as differential gene expression analysis using the pseudobulk method was also performed. No significant differences in cellular composition or gene expression were observed between the two treatment arms. Using BioQC, we identified a few cell types that showed changed expression of IL‐1 signature genes, especially inflammatory monocytes, which is in line with the expected mode of action of selnoflast (Figure 7B).

In summary, although the effects of selnoflast on the IL‐1 gene signature showed a trend in the right direction, the effect was rather modest compared with that of other therapeutic agents such as anti‐TNF or anti‐IL‐6 antibodies.^28^, ^29^

## 4 DISCUSSION

This study evaluated the safety, PK and PD of the NLRP3 inflammasome inhibitor selnoflast in patients with moderate to severe active UC.

Selnoflast was safe and well‐tolerated in patients with UC. The reported AEs were non‐serious, and the majority were of mild intensity. None led to dose interruption or treatment discontinuation. There were no deaths or SAEs.

The PK findings were consistent with those from a previous Entry‐into‐Humans study in healthy subjects (IZD334‐001; ClinicalTrials.gov NCT04086602), which tested doses from 20 to 600 mg under fasted conditions (unpublished results). After single administration in healthy subjects, there was a rapid increase in selnoflast plasma concentrations with a median T_max_ of 0.5–6.0 h (1.0–2.0 h in UC patients in the present study), followed by a mono‐exponential decrease. Systemic exposure increased in an approximately dose‐proportional manner; exposure and clearance following multiple administration were generally comparable to those observed after single administration (unpublished results). Steady‐state was achieved on Day 2 in both healthy subjects and UC patients. At steady state, there were quantifiable levels of selnoflast in sigmoid colon tissue of all UC patients in the active treatment arm. Taken together, these results suggest that once daily dosing with 450 mg selnoflast was sufficient to achieve the plasma and tissue exposure predicted to maintain the targeted level of inhibition (i.e., IC_90_) of the NLRP3 inflammasome over the dosing interval.

In line with this, there was a rapid and sustained inhibition of mature IL‐1β release ex vivo in blood upon LPS stimulation after the first oral administration of selnoflast. These findings suggest target engagement with the NLRP3 inflammasome. On the other hand, there were only modest differences between the treatment arms in terms of biomarker response in the sigmoid colon tissue—reflecting only minor reduction of tissue IL‐1β. The contrast between the robust *ex vivo* response to selnoflast treatment (inhibition of IL‐1β release in LPS‐stimulated whole blood) against the relative lack of response in the sigmoid colon suggests that mechanisms other than the NLRP3 inflammasome play a more prominent role in IL‐1β maturation in sigmoid colon tissue from UC patients. Despite evidence supporting the role of the NLRP3 inflammasome in UC pathogenesis, findings from recent studies have revealed a more complex picture.^14^ Preclinical data using various animal models of IBD indicate a dual, stage‐dependent role of the NLRP3 inflammasome^30^ suggesting that in the early stages of the disease, its activation could promote repair and regeneration of the intestinal mucosa.^14^ Similar findings have been uncovered in other disease areas where the NLRP3 inflammasome is a potential therapeutic target.^31^, ^32^ In addition to differences in experimental techniques, it is likely that other factors, such as intestinal microbiome composition and genetic background, may tip the balance between the ability of the NLRP3 inflammasome to protect or aggravate the intestinal milieu.^33^ A recent study reported that adding anakinra to standard‐of‐care therapy in patients with acute severe UC did not improve the clinical outcome,^34^ adding to the evidence that blockade of IL‐1β is not a silver bullet for UC.

There was a marked reduction from baseline in neutrophils in the sigmoid colon tissue of three selnoflast‐treated patients, but no significant changes were observed in the active treatment arm as a whole. There were no clinical or histological effects observed, i.e. in the Geboes Score or Nancy Index, as expected for a study of only 1‐week duration.

In our study, we used single‐cell RNA sequencing as an investigational tool for evaluating the effects of selnoflast on changes in cell composition and an IL‐1 gene signature in sigmoid colon tissue. None revealed major changes as would be expected if NLRP3 was a major driver of inflammation in active UC. Most published single‐cell RNA‐sequencing data from UC patients are derived from observational studies.^26^, ^35^, ^36^ The experimental nature of the dataset generated in this randomized, controlled, and double‐blind study makes it a valuable asset for understanding both the etiology and progression of UC in its natural course as well in the presence of NLRP3‐targeting therapy.

The study was an innovative Phase 1b / proof of mechanism study, with the aim of reaching a decisive outcome using a rapid and lean design. This lean design naturally led to the limitations of our study, which are the small sample size and the short treatment duration. In lieu of validated assays for PD markers in tissue (e.g., mature IL‐1β), we analyzed an IL‐1 gene signature relevant in the context of IBD. Therefore, another limitation of this study is that we could only indirectly assess the effect of selnoflast on IL‐1β in colon tissue. Despite these limitations, the results from this study enabled us to reach the decision to terminate the development of selnoflast in UC within a rapid timeframe.

## 5 CONCLUSIONS

Selnoflast 450 mg QD administered over 7 consecutive days was well‐tolerated and achieved the plasma and tissue exposure predicted to maintain the targeted level of NLRP3 inflammasome inhibition. However, histological analysis, expression of an IL‐1 gene signature in sigmoid colon tissue and additional biomarker analyses showed no robust differences between treatment arms, suggesting that no major therapeutic effects are to be expected in UC.

## CONFLICT OF INTEREST STATEMENT

BK, LP, AN, SN, AB, SD, PG, PS, JDZ, CFC and AC were employees of F. Hoffmann‐La Roche AG Switzerland, and LB was an employee of Roche Products Ltd., UK, during the conduct of the study. JH and SB were consultants.

## FUNDING INFORMATION

F. Hoffmann‐LaRoche AG

## Supporting information

Supporting Information

Supporting Information

## Data Availability

Clinical study documentation can be requested via the following link: https://www.roche.com/research_and_development/who_we_are_how_we_work/clinical_trials/our_commitment_to_data_sharing/clinical_study_documents_request_form.htm
