# Supplementary material for: A randomized, double‐blind phase 1b study evaluating the safety, tolerability, pharmacokinetics and pharmacodynamics of the NLRP3 inhibitor selnoflast in patients with moderate to severe active ulcerative colitis
Source: Clin Transl Med. 2023 Nov 14;13(11):e1471. doi: 10.1002/ctm2.1471 (PMC10644327; doi:10.1002/ctm2.1471)
Supplement: Supplementary file 1 — Supporting Information [file CTM2-13-e1471-s001.docx]

**SUPPLEMENTARY INFORMATION**

Manuscript title: A randomized, double-blind Phase 1b study evaluating the safety, tolerability, pharmacokinetics and pharmacodynamics of the NLRP3 inhibitor selnoflast in patients with moderate to severe active ulcerative colitis

Authors: Barbara Klughammer1, Luca Piali1, Alexandra Nica1, Sandra Nagel1, Lorna Bailey2, Christoph Jochum3, Stanislav Ignatenko4, Angela Bläuer1, Sabrina Danilin1, Pratiksha Gulati1, Joanne Hayward5, Petar Scepanovic1, Jitao David Zhang1, Satish Bhosale6, Chui Fung Chong1*, Andreas Christ1*

Affiliations:

1 F. Hoffmann-La Roche AG, Basel, Switzerland

2 Roche Products Limited, Welwyn Garden City, United Kingdom

3 Charité - Universitätsmedizin Berlin, Berlin, Germany

4 Charité Research Organisation GmbH, Berlin, Germany

5 A4P Bio, Sandwich, United Kingdom

6 IQVIA RDS (India) Pvt Ltd, Thane, India

* equal contributors

Corresponding author:

Dr. Barbara Klughammer,

Roche Pharma Research and Early Development, Roche Innovation Center Basel,

F. Hoffmann-La Roche AG, Grenzacherstrasse 124,

4070 Basel, Switzerland.

Email: [barbara.klughammer@roche.com](mailto:barbara.klughammer@roche.com)

**PROTOCOL**

**TITLE: RANDOMIZED, DOUBLE-BLIND, SPONSOR- OPEN, PHASE 1B STUDY TO ASSESS THE SAFETY, PHARMACOKINETICS AND TO EXPLORE THE PHARMACODYNAMICS OF RO7486967 IN PATIENTS WITH MODERATE TO SEVERE ACTIVE ULCERATIVE COLITIS**

**PROTOCOL NUMBER:** BP43099

**VERSION:** 3

**EUDRACT NUMBER:** 2021-000557-85

**IND NUMBER:** NA

**TEST PRODUCT:** RO7486967

**SPONSOR:** F. Hoffmann-La Roche Ltd

**DATE FINAL:** See electronic date stamp below

**FINAL PROTOCOL APPROVAL**

**Date and Time (UTC)**

01-Oct-2021 13:22:26

**Title**

Company Signatory

**Approver's Name**

Junker, Uwe (junkeru1)

**CONFIDENTIAL**

The information contained in this document, especially any unpublished data, is the property of F. Hoffmann-La Roche Ltd (or under its control) and therefore, is provided to you in confidence as an Investigator, potential Investigator, or consultant, for review by you, your staff, and an applicable Ethics Committee or Institutional Review Board. It is understood that this information will not be disclosed to others without written authorization from Roche except to the extent necessary to obtain informed consent from persons to whom the drug may be administered.

RO7486967 **—F. Hoffmann-La Roche Ltd**

Protocol BP43099, Version 3

**PROTOCOL ACCEPTANCE FORM**

**TITLE: RANDOMIZED, DOUBLE-BLIND, SPONSOR- OPEN, PHASE 1B STUDY TO ASSESS THE SAFETY, PHARMACOKINETICS AND TO EXPLORE THE PHARMACODYNAMICS OF RO7486967 IN PATIENTS WITH MODERATE TO SEVERE ACTIVE ULCERATIVE COLITIS**

**PROTOCOL NUMBER:** BP43099

**VERSION NUMBER:** 3

**EUDRACT NUMBER:** 2021-000557-85

**IND NUMBER:** NA

**TEST PRODUCT:** RO7486967

**SPONSOR:** F. Hoffmann-La Roche Ltd

**I agree to conduct the study in accordance with the current protocol.**

Principal Investigator’s Name (print)

Principal Investigator’s Signature Date

Please keep the signed original form in your study files, and return a copy to your local

Site Monitor.

**PROTOCOL AMENDMENT, VERSION 3**

**RATIONALE**

Protocol BP43099 has been amended to clarify the reporting period of the Investigator to Sponsor in case of serious undesirable effects (SUE)/ serious adverse events (SAE) as per Health Authority request. The Investigator must report such events to the Sponsor immediately (i.e., without undue delay).

New information appears in *Book Antiqua* italics.

**TABLE OF CONTENTS**

PROTOCOL ACCEPTANCE FORM .................................................................... 2

LIST OF ABBREVIATIONS AND DEFINITIONS OF TERMS............................... 9

| 1. | PR | OTOCOL SUMMARY............................................................................. 11 |
| --- | --- | --- |
|  | 1.1 | Synopsis ................................................................................ 11 |
|  | 1.2 | Schematic of Study Design.................................................... 17 |
|  | 1.3 | Schedule of Activities ............................................................ 17 |

2. INTRODUCTION ........................................................................................ 20

2.1 Study Rationale ..................................................................... 20

2.2 Background ........................................................................... 20

2.2.1 Ulcerative Colitis .................................................................... 20

2.2.2 RO7486967 ........................................................................... 21

2.3 Benefit/Risk Assessment ....................................................... 21

3. OBJECTIVES AND ENDPOINTS ............................................................... 24

4. STUDY DESIGN ......................................................................................... 25

4.1 Overall Design ....................................................................... 25

4.1.1 Length of the Study ............................................................... 25

4.1.2 Stopping Rules Criteria.......................................................... 25

4.1.3 Individual Stopping Criteria.................................................... 26

4.1.4 Communication Strategy ....................................................... 26

4.2 Scientific Rationale for Study Design..................................... 26

4.2.1 Rationale for Study Population .............................................. 26

4.2.2 Rationale for Hospitalization .................................................. 27

4.2.3 Rationale for Control Group................................................... 27

4.2.4 Rationale for Biomarker Assessments................................... 27

4.3 Justification for Dose ............................................................. 28

4.4 End of Study Definition .......................................................... 28

5. STUDY POPULATION................................................................................ 29

5.1 Inclusion Criteria .................................................................... 29

5.2 Exclusion Criteria................................................................... 30

5.3 Lifestyle Considerations......................................................... 32

5.3.1 Meals and Dietary Restrictions .............................................. 32

5.3.2 Alcohol and Tobacco ............................................................. 33

5.3.3 Activity ................................................................................... 33

5.4 Screen Failures ..................................................................... 33

6. TREATMENTS............................................................................................ 34

6.1 Treatments Administered....................................................... 34

6.2 Preparation/Handling/Storage/Accountability ........................ 34

6.3 Measures to Minimize Bias: Randomization and

Blinding.................................................................................. 36

6.3.1 Method of Treatment Assignment.......................................... 36

6.3.2 Blinding.................................................................................. 36

6.4 Treatment Compliance .......................................................... 37

6.5 Concomitant Therapy ............................................................ 37

6.5.1 Permitted Therapy ................................................................. 37

6.5.2 Prohibited Therapy ................................................................ 38

6.6 Dose Modification .................................................................. 39

6.7 Treatment after the End of the Study..................................... 39

7. DISCONTINUATION OF STUDY, STUDY TREATMENT AND

PARTICIPANT DISCONTINUATION/WITHDRAWAL................................. 39

7.1 Discontinuation of Study Treatment....................................... 39

7.2 Participant Discontinuation/Withdrawal from the

Study ..................................................................................... 40

7.3 Lost to Follow-Up................................................................... 40

8. STUDY ASSESSMENTS AND PROCEDURES ......................................... 41

8.1 Safety Assessments .............................................................. 41

8.1.1 Physical Examinations........................................................... 41

8.1.2 Vital Signs.............................................................................. 42

8.1.3 Electrocardiograms................................................................ 42

8.1.4 Clinical Safety Laboratory Assessments................................ 43

8.1.5 Safety Biomarker Assessments ............................................. 44

8.1.6 Medical History and Demographic Data ................................ 44

8.2 Adverse Events and Serious Adverse Events ....................... 45

8.2.1 Time Period and Frequency for Collecting Adverse Event and Serious Adverse Event Information............................................................................. 45

8.2.2 Method of Detecting Adverse Events and Serious

Adverse Events ..................................................................... 46

8.2.3 Follow-Up of Adverse Events and Serious

Adverse Events ..................................................................... 46

8.2.3.1 Investigator Follow-Up ........................................................... 46

8.2.3.2 Sponsor Follow-Up ................................................................ 46

8.2.4 Regulatory Reporting Requirements for Serious

Adverse Events ..................................................................... 47

8.2.4.1 Emergency Medical Contacts ................................................ 47

8.2.5 Pregnancy ............................................................................. 47

8.2.6 Non-Serious Adverse Events of Special Interest ................... 48

8.2.7 Disease-Related Events and/or Disease-Related

Outcomes Not Qualifying as Adverse Events or

Serious Adverse Events ........................................................ 48

8.2.8 Management of Specific Adverse Events .............................. 48

8.3 Treatment of Overdose.......................................................... 50

8.4 Pharmacokinetics .................................................................. 51

8.4.1 Gut Tissue Concentration ...................................................... 51

8.5 Pharmacodynamics and Biomarkers Analyses...................... 52

8.5.1 Genetic and Genomic Analyses ............................................ 52

8.5.1.1 Clinical Genotyping................................................................ 52

8.6 Pharmacodynamics and Biomarker Samples ........................ 52

8.6.1 Mandatory Samples............................................................... 53

8.6.1.1 Blood Sampling ..................................................................... 53

8.6.1.2 Stool Sampling ...................................................................... 53

8.6.1.3 Tissue Sampling .................................................................... 54

8.7 Samples for Research Biosample Repository ....................... 54

8.7.1 Overview of the Research Biosample Repository.................. 54

8.7.2 Sample Collection.................................................................. 55

8.8 Timing of Study Assessments ............................................... 56

8.8.1 Screening and Pre-treatment Assessments .......................... 56

8.8.2 Assessments during Treatment ............................................. 57

8.8.3 Assessments at Study Completion/Early

Withdrawal Visit ..................................................................... 57

8.8.4 Follow-Up Assessments ........................................................ 57

8.8.5 Assessments at Unscheduled Visits ...................................... 57

9. STATISTICAL CONSIDERATIONS ............................................................ 58

9.1 Statistical Hypotheses ........................................................... 58

9.2 Sample Size Determination ................................................... 58

9.3 Populations for Analyses ....................................................... 58

9.4 Statistical Analyses................................................................ 58

9.4.1 Demographics and Baseline Characteristics ......................... 58

9.4.2 Efficacy Analyses .................................................................. 59

9.4.3 Safety Analyses ..................................................................... 59

9.4.4 Pharmacokinetic Analyses..................................................... 59

9.4.5 Pharmacodynamic Analyses ................................................. 60

9.4.6 Pharmacokinetic/Pharmacodynamic

Relationships ......................................................................... 61

9.5 Summaries of Conduct of Study ............................................ 61

10. REFERENCES ........................................................................................... 62

11. SUPPORTING DOCUMENTATION AND OPERATIONAL CONSIDERATIONS.................................................................................... 63

**LIST OF TABLES**

Table 1 Schedule of Activities .................................................................. 18

Table 2 Schedule of Activities - Detailed Table........................................ 19

Table 3 Objectives and Endpoints ........................................................... 24

Table 4 Summary of Treatments Administered........................................ 34

Table 5 Guidelines for Managing Liver Function Test Abnormalities ....... 49

Table 6 Guidelines for Managing Infections ............................................. 50

Table 7 Analysis Populations ................................................................... 58

Table 8 Safety Statistical Analysis Methods ............................................ 59

**LIST OF FIGURES**

Figure 1 Overview of Study Design........................................................... 17

**LIST OF APPENDICES**

Appendix 1 Regulatory, Ethical, and Study Oversight Considerations........... 64

Appendix 2 Adverse Events: Definitions and Procedures for Evaluating,

Follow-up, and Reporting ............................................................ 72

Appendix 3 Procedures for Recording Adverse Events ................................. 81

Appendix 4 Clinical Laboratory Tests ............................................................ 86

Appendix 5 Contraceptive and Barrier Guidance ........................................... 89

**LIST OF ABBREVIATIONS AND DEFINITIONS OF TERMS**

**Abbreviation Definition**

**AE** adverse event

**ALP** alkaline phosphatase

**ALT** alanine aminotransferase **AST** aspartate aminotransferase **AUC** area under the curve

**BCRP** Breast cancer resistance protein

**BMI** body mass index **BP** blood pressure **CL** clearance

**CL/F** apparent clearance

**Cmax** maximum concentration

**COVID-19** Coronavirus disease-19

**CRP** c-reactive protein

**CSR** clinical study report

**C-SSRS** Columbia-Suicide Severity Rating Scale

**EC** Ethics Committee

**ECG** electrocardiogram

**eCRF** electronic case report form

**EDC** electronic data capture

**EU** European Union

**FDA** Food and Drug Administration **FSH** follicle-stimulating hormone **GCP** Good Clinical Practice

**GLP** Good Laboratory Practice **HBsAg** Hepatitis B surface antigen **HBcAb** total hepatitis B core antibody **HBV** Hepatitis B

**HCV** Hepatitis C

**HIV** human immunodeficiency virus **HRT** hormonal replacement therapy **IB** Investigator’s Brochure

**ICF** Informed Consent Form

**ICH** International Council on Harmonisation

**IL** interleukin

**IMP** investigational medicinal product

**INR** international normalized ratio

**IRB** Institutional Review Board

**IxRS** interactive (voice/web) response system

**LDH** lactate dehydrogenase **LH** luteinizing hormone **LPS** lipopolysaccharide **LPLV** last participant last visit

**MAD** multiple-ascending doses

**NCI CTCAE** National Cancer Institute Common Terminology Criteria for Adverse Events

**NLRP** Nucleotide-binding oligomerization domain-Like

Receptor family Pyrin

**NOAEL** no-observed-adverse-effect level

**NSAESI** non-serious adverse event of special interest

**OTC** over-the-counter

**P-gp** P-glycoprotein

**PD** pharmacodynamic **PK** pharmacokinetic **PT** prothrombin time **QD** once daily

**RBC** red blood cell

**RBR** Research Biosample Repository

**RNA** Ribonucleic acid

**SAD** single-ascending dose

**SAE** serious adverse event

**SARS-CoV-2** Severe acute respiratory syndrome coronavirus 2

**SoA** schedule of activities

**SOP** standard operating procedure

**TBL** total bilirubin

**TNF** tumor necrosis factor

**Tmax** time of maximum concentration observed

**UC** Ulcerative Colitis **ULN** upper limit of normal **US** United States

**V/F** apparent volume of distribution

**WBC** white blood cell

**WES** whole exome sequencing **WGS** whole genome sequencing **WOCBP** women of childbearing potential

**WONCBP** women of non-childbearing potential

**1. PROTOCOL SUMMARY**

**1.1 SYNOPSIS**

**PROTOCOL TITLE: RANDOMIZED, DOUBLE-BLIND, SPONSOR-OPEN, PHASE 1B STUDY TO ASSESS THE SAFETY, PHARMACOKINETICS AND TO EXPLORE THE PHARMACODYNAMICS OF RO7486967 IN PATIENTS WITH MODERATE TO SEVERE ACTIVE ULCERATIVE COLITIS**

**SHORT TITLE Phase 1b to assess Safety, Pharmacokinetics and Exploratory**

**Pharmacodynamics in Patients with Ulcerative Colitis**

**PROTOCOL NUMBER:** BP43099

**VERSION:** 3

**TEST PRODUCT:** RO7486967

**PHASE:** Ib

**RATIONALE**

Inflammasomes are large multimeric protein complexes that actively regulate immunity and homeostasis through sensing and responding to microbial or other danger signals. The nucleotide-binding oligomerization domain-like receptor family pyrin 3 (NLRP3) inflammasome is expressed in immune cells of the gut and promotes inflammation through

caspase-1-dependent cleavage and activation of immature pro-inflammatory cytokines interleukin (IL)-1β and IL-18, as well as by induction of pyroptotic cell death. Locally, IL-1β can induce cytokine production, enhance T-cell activation and antigen recognition, and direct neutrophils to the site of injury. NLRP3 and IL-1β are both upregulated in active ulcerative colitis (UC). Several animal colitis model studies reported that inhibition of IL-1β function by methods that operate downstream of the NLRP3 inflammasome, resulting in reduced gut inflammation.

RO7486967 is a selective and reversible small molecule NLRP3 inflammasome inhibitor that has been shown to inhibit IL-1β release and cell death in vitro.

This study will assess the safety, tolerability, pharmacokinetics (PK) of RO7486967 and pharmacodynamic markers of inflammation in patients with moderate to severe active UC.

**OBJECTIVES AND ENDPOINTS**

| **Objectives Endpoints** |
| --- |
| **Primary** |
| • To assess the safety and tolerability • Incidence and severity of adverse of orally administered RO7486967 in events.  participants with active UC.  • Changes in vital signs, ECG parameters,  and clinical laboratory safety parameters.  • To investigate the PK of orally • Pharmacokinetic parameters of administered RO7486967 in blood of RO7486967 (and its metabolites, as participants with active UC. applicable) in blood. |
| **Secondary** |
| • To evaluate the effects on • Changes in C-reactive protein (CRP) in inflammation markers. blood and calprotectin in stool. |

**OVERALL DESIGN Study Design**

Study BP43099 is a randomized, placebo-controlled, Investigator- and patient-blind,

Sponsor-open study to assess safety, tolerability, and PK of RO7486967 in patients with active,

moderate to severe UC. The study will utilize two treatment arms: placebo versus 450 mg

RO7486967 administered orally once daily (QD).

All participants in the study will have been diagnosed with UC previously and be in an active stage of their disease.

After the screening period, participants will be randomized to receive either placebo or

RO7486967 orally (3 capsules of 150 mg QD). Randomization (treatment assignment) will be

2:1 (RO7486967 to placebo) and occur on Day −1. Treatment duration will be 7 days. Participants will have biopsies taken from the rectum (rectoscopy) between Day −3 and Day −1

and on Day 7 predose (see Figure 1).

Participants will be hospitalized from Day −1 to Day 7. They will return to the clinic for the follow- up visit on Day 14.

Dose adjustments are not foreseen during the study.

**Treatment Groups and Duration**

The investigational medicinal products for this study are RO7486967 and placebo.

**Length of Study**

The study length will be approximately 6 weeks (from screening through to follow-up), for each enrolled participant, as follows:

Screening: Up to 28 days. Treatment period: Days 1 to 7.

Follow-up: 7 (± 3) days after last study treatment administration.

**End of Study**

The end of the study is defined as the date when the last participant last visit (LPLV) occurs. LPLV is expected to occur approximately 2 weeks after the last participant is randomized.

**PARTICIPANT POPULATION**

Participants of this study are male and female patients of 18 to 75 years of age (inclusive), with active, moderate to severe UC.

**INCLUSION/EXCLUSION CRITERIA INCLUSION CRITERIA**

Participants are eligible to be included in the study only if all of the following criteria apply.

**Informed Consent**

1. Able and willing to provide written informed consent and to comply with the study protocol according to International Conference on Harmonisation and local regulations. A signed Informed Consent Form must be available from the participant before starting any

study-specific assessments.

**Age**

2. Between 18 to 75 years of age (inclusive), at the time of signing the informed consent.

**Type of Participants and Disease Characteristics**

3. Diagnosis of UC at least 12 weeks prior to screening.

4. Patients with active moderate to severe UC as measured by partial MCS ≥ 3 and ≤ 8, with stool frequency subscore ≥ 1, rectal bleeding subscore ≥ 1, and fecal calprotectin ≥ 150

μg/g.

5. Most recent colonoscopy within the preceding 3 years at the time of screening.

6. Screening colonoscopy for colorectal cancer conducted within the prior two years if:

• History of pancolitis and disease duration ≥ 8 years, or

• History of left-sided colitis and disease duration ≥ 12 years.

**Weight**

7. Body mass index within the range of 18-35 kg/m2 (inclusive).

**Sex and Contraceptive/Barrier Replacement**

8. Male and female participants

The reliability of sexual abstinence for female enrollment eligibility needs to be evaluated in relation to the duration of the clinical study and the preferred and usual lifestyle of the participant. Periodic abstinence (e.g., calendar, ovulation, symptothermal, or post-ovulation methods) and withdrawal are not acceptable methods of preventing drug exposure.

a) Female participants

A female participant is eligible to participate if she is not pregnant, not breastfeeding, and at least one of the following conditions applies:

• Women of non-childbearing potential (WONCBP—i.e., post-menopausal;

pre-menarchal; or pre-menopausal with documented hysterectomy, bilateral

salpingectomy, or bilateral oophorectomy).

• WOCBP (a woman is considered fertile following menarche and until becoming post-menopausal unless permanently sterile), who:

o Agree to remain abstinent (refrain from heterosexual intercourse) or use at least 1 acceptable contraceptive methods for at least 1 month before dosing, during the treatment period and for at least 7 days after the final dose of RO7486967/placebo.

The following are acceptable contraceptive methods: bilateral tubal occlusion, male sterilization, established proper use of hormonal contraceptives that inhibit ovulation, hormone-releasing intrauterine devices and copper intrauterine devices, male or female condom with or without spermicide; and cap, diaphragm, or sponge with spermicide.

b) Male participants

No contraceptive requirements for male participants.

**EXCLUSION CRITERIA:**

Participants are excluded from the study if any of the following criteria apply:

**Medical Conditions**

1. Diagnosis of fulminant UC, Crohn’s disease, indeterminate colitis, microscopic colitis, segmental colitis associated with diverticulosis, ischemic colitis, or radiation-induced colitis based on medical history, endoscopy, and/or histological findings.

2. Active infections requiring systemic therapy with antibiotic, antiviral, or antifungal medication or febrile illness within 7 days before Day −1.

3. History of primary or acquired immunodeficiency.

4. History of chronic pulmonary disease with resultant clinically significant abnormal pulmonary function.

5. History of clinically significant cardiac or cardiovascular disease or uncontrolled hypertension.

6. Presence of chronic liver disease.

7. Evidence of colonic dysplasia that cannot be completely removed.

8. Women: Pregnant or lactating.

9. Any condition or disease detected during the medical interview/physical examination that would render the patient unsuitable for the study, place the patient at undue risk, or interfere with the ability of the patient to complete the study in the opinion of the Investigator.

10. History of tuberculosis or a positive Quantiferon® Gold test.

11. History of clinically significant severe drug allergies, multiple drug allergies, or allergy to any constituent of the investigational medicinal product.

12. Lymphoma, leukemia, or any malignancy within the past 10 years, except for basal cell or squamous epithelial carcinomas of the skin that have been resected with no evidence of metastatic disease for 3 years and in situ carcinoma of the cervix that was completely removed surgically.

13. History or presence of clinically significant ECG abnormalities before study treatment administration (e.g., PQ/PR interval ≥ 220 ms, QT corrected for heart rate using Fridericia’s correction factor (QTcF), < 350 or ≥ 450 ms) or clinically significant cardiovascular disease (e.g., cardiac insufficiency, coronary artery disease, cardiomyopathy, congestive heart failure, family history of congenital long QT interval syndrome, family history of sudden death).

14. Fecal microbiota transplant, defined as receipt of any product derived from the feces of another human and administered per oral, per nasogastric or nasoduodenal, or per rectum within the last 6 months.

15. Bowel surgery within 12 months prior to study start.

16. History of colectomy or partial colectomy.

17. History of known bleeding disorder or diseases with an increased risk of bleeding tendency.

**Prior/Concomitant Therapy**

18. Use of calcineurin inhibitors (e.g., tacrolimus, cyclosporine), vedolizumab, ustekinumab, anti-TNFα therapeutic or any other immune system−targeted therapy within 12 weeks or

5 half-lives, whichever is longer, prior to screening.

19. Rectal therapy with 5-ASA or corticosteroids within 2 weeks of screening.

20. Leukocyte apheresis within 12 weeks of screening.

21. Vaccine(s) within four weeks prior to first dose, or plans to receive vaccines during the study or within 28 days of the last dose, with the exception of SARS-CoV-2 vaccination, which could be given 14 days after last dose.

**Prior/Concurrent Clinical Study Experience**

22. Donation of blood or blood products in excess of 500 mL within 3 months.

23. Exposure to more than 4 investigational treatments within 12 months prior to Day 1.

24. Use of any investigational drug or any other type of interventional medical research within

12 weeks of screening.

**Diagnostic Assessments**

25. Abnormal hematologic values:

• Anemia (hemoglobin < 10 g/dL)

• Leukocytosis (white blood cells ≥ 2 × ULN)

• Neutropenia < 1000/μL

• Thrombocytopenia (platelet count < 100,000/μL)

• Thrombocytosis (platelet count ≥ 2 × ULN)

• Eosinophilia (eosinophil count ≥ 2 × ULN)

26. Renal function: creatinine clearance < 60 mL/minute.

27. Abnormal hepatic enzyme or hepatic function values:

• ALT, AST, alkaline phosphatase (ALP), total bilirubin or gamma-glutamyl transferase

(GGT) ≥ 1.5 × ULN (unless total bilirubin ≥ 1.5 × ULN is caused by Gilbert’s disease).

• International normalized ratio (INR) > ULN

• Albumin < 3 g/dL

28. Cardiac troponin I outside the normal range.

29. Positive HIV antibody test.

30. Presence of hepatitis B surface antigen (HBsAg). If the patient has positive total hepatitis B core antibody (HBcAb), a negative PCR test for hepatitis B DNA (< 20 IU/mL) is required for the patient to be eligible.

31. Positive hepatitis C by PCR test result at screening or within the 3 months prior to starting study treatment.

**Other Exclusions**

32. History of regular alcohol consumption within 2 months of screening defined as: An average weekly intake of > 14 drinks for men or > 7 drinks for women. One drink is equivalent to 12 g of alcohol: 12 ounces (360 mL) of beer, 5 ounces (150 mL) of wine, or 1.5 ounces (45 mL) of

80 proof distilled spirits.

33. Any suspicion or history of alcohol abuse and/or suspicion of regular consumption of drug of abuse.

34. Patients under judicial supervision, guardianship, or curatorship.

35. Patients dependent on the Sponsor, the participating clinic(s) or the Investigator(s).

**NUMBER OF PARTICIPANTS**

The study is expected to enroll 18 patients in total, with 6 in the placebo arm and 12 in the treatment arm. Sample size is based on feasibility.

**CONCOMITANT MEDICATIONS**

All medications (prescription and over-the counter) taken within 4 weeks of study screening and throughout the duration of the study will be recorded on the appropriate electronic Case Report Form.

**Permitted Therapy**

Use of the following therapies will be permitted, as specified below:

• Paracetamol is allowed up to a maximum dose of 2 g/day.

• Participants who use the following therapies prior to screening and are on a stable regimen should continue their use:

o Oral contraceptives, hormone-replacement therapy, or other maintenance therapy.

• Participants who are taking non-biologic standard of care (SOC) therapy for UC during the study will remain on their SOC therapy. Specific instructions for each SOC is provided below:

o Stable therapy for at least 2 weeks prior to screening with:

– oral 5-aminosalicylic acid, and/or

– oral glucocorticoid (not to exceed 20 mg/day prednisone equivalent).

o Stable therapy for at least 8 weeks with:

– azathioprine, or

– 6-mercaptopurine, or

– methotrexate (and folic acid)

**Prohibited Therapy**

As a general rule, no concomitant medication will be permitted, with the exception of medications to treat AEs and those specified as permitted therapies, unless the rationale for exception is discussed and clearly documented between the Investigator and the Sponsor.

Vaccines: See exclusion criterion 21; with the exception of SARS-CoV-2 vaccines which would be permitted at least 14 days after the last dose of RO7486967/placebo.

Use of the following therapies will be prohibited during the study and for at least 14 days or at least 5 half-lives prior to initiation of study treatment, whichever is longer unless otherwise specified below:

• Any rectal therapy.

• Doses of glucocorticoids exceeding 20 mg/day of prednisone or equivalent.

• Non-steroidal anti-inflammatory medications (e.g., ibuprofen, naproxen) due to a propensity for these treatments to worsen inflammation in UC.

• Calcineurin inhibitors (e.g., tacrolimus, cyclosporine) and any immune system targeted therapy within 12 weeks or 5 half-lives, whichever is longer, before the start of screening.

• Antibiotics, antifungals, antivirals throughout treatment duration.

Strong P-glycoprotein (P-gp) inhibitors (e.g., amiodarone, carvedilol, clarithromycin), will be prohibited 7 days prior to study treatment initiation and throughout the study.

**1.2 SCHEMATIC OF STUDY DESIGN**

An overview of the study design is provided in Figure 1.

**Figure 1 Overview of Study Design**


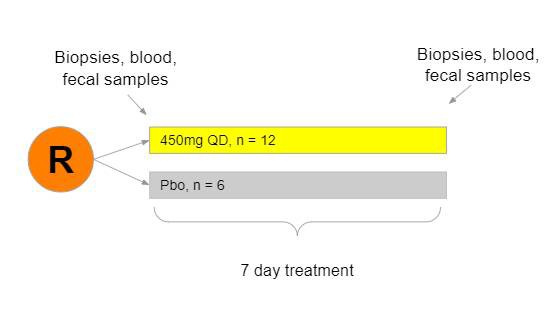


R = Randomization

**1.3 SCHEDULE OF ACTIVITIES**

The schedules of activities (SoAs) are provided in Table 1 and Table 2.

**Table 1 Schedule of Activities**

| **Version 2021-09-06 Visit Name / Day** | **Screening day -29 to -2** | **-1** | **1** | **2** | **3** | **4** | **5** | **6** | **7** | **Unscheduled**  **Visit** | **Early**  **Withdrawal** | **Follow Up Visit day 14**  **or early w ithdraw al +7** |
| --- | --- | --- | --- | --- | --- | --- | --- | --- | --- | --- | --- | --- |
| **Location / Visit Window / assessment specification** |  | **in clinic** | **in clinic** | **in clinic** | **in clinic** | **in clinic** | **in clinic** | **in clinic** | **in clinic** | **Assessments as needed** | **Assessments as deemed feasible** | **±3 days** |
| **Informed Consent** | **x** |  |  |  |  |  |  |  |  |  |  |  |
| **Demography** | **x** |  |  |  |  |  |  |  |  |  |  |  |
| **Medical History** | **x** |  |  |  |  |  |  |  |  |  |  |  |
| **Tuberculosis Screening Test** | **x** |  |  |  |  |  |  |  |  |  |  |  |
| **Testing for Clostridium difficile infection** | **x** |  |  |  |  |  |  |  |  |  |  |  |
| **Testing for SARS-CoV-2 (if not vaccinated or recovered)** | **x** | **x** |  |  |  |  |  |  |  |  | **x** | **x** |
| **Drugs of Abuse + Alcohol Test** | **x** |  |  |  |  |  |  |  |  |  |  |  |
| **Viral testing: HBV, HCV, HIV,** | **x** |  |  |  |  |  |  |  |  |  |  | **x a** |
| **Hormones (females only)** | **x** |  |  |  |  |  |  |  |  |  |  |  |
| **Previous and Concomitant Treatments** | **x** | **any time** | | | | | | | | | | |
| **Diary to assess MSC** | **x** |  | | | | | | | | | | |
| **Treatment assignment** |  | **x** |  |  |  |  |  |  |  |  |  |  |
| **Administration of Study Medication** |  |  | **x** | **x** | **x** | **x** | **x** | **x** | **x** |  |  |  |
| **Anthropometric Measurements (incl. Weight,** | **x** | **x** | **x** | **x** | **x** | **x** | **x** | **x** | **x** | **x** | **x** | **x** |
| **Vital Signs** | **x** | **x** | **7 b** | **x** | **x** | **x** | **7 b** | **x** | **x** | **x** | **6 c** | **x** |
| **Full physical examination** | **x** | **x** |  | **x** |  |  |  |  | **x** | **x** | **x** | **x** |
| **ECG-12 lead** | **x** |  | **7 b** |  |  |  | **7 b** |  | **x** | **x** | **6 c** | **x** |
| **Hematology** | **x** | **x** |  | **x** |  |  | **x** |  | **x** | **x** | **x** | **x** |
| **Coagulation** | **x** | **x** |  |  |  |  | **x** |  | **x** | **x** | **x** | **x** |
| **Blood Chemistry** | **x** | **x** |  | **x** |  |  | **x** |  | **x** | **x** | **x** | **x** |
| **Urinalysis** | **x** | **x** |  |  |  |  | **x** |  | **x** | **x** | **x** | **x** |
| **Pregnancy Test (WOCBP only)** | **x** | **x** |  |  |  |  |  |  |  |  |  | **x** |
| **Rectal biopsy** |  | **x d** |  |  |  |  |  |  | **x** |  | **x** |  |
| **Stool** | **x** | **x e** | **x f** | **x f** | **x f** | **x f** | **x f** | **x f** | **x f** |  | **x f** | **x** |
| **Clinical Genotyping** |  | **x g** |  |  |  |  |  |  |  |  |  |  |
| **PK Sample** |  |  | **7 b** | **x h** | **x** | **x** | **7 b** | **x h** | **x** | **x** | **6 c** | **x** |
| **ex-vivo cytokine release** |  |  | **7 b** | **x h** |  |  | **7 b** | **x h** | **x** |  | **6 c** | **x** |
| **Soluble PD biomarkers (serum / plasma)** |  | **x** | **x i** |  |  |  | **x i** |  | **x i** |  | **x i** | **x** |
| **Research Biosample Repository (RBR) Consent** | **Optional: If patients agrees to donate left over samples: preferred at day 1 but can be collected any tim e during the study** | | | | | | | | | | |  |
| **Exploratory Safety Sample** |  | **x** | **In case of Liver Function Test Abnormalities (see section 8.2.8)** | | | | | | | | |  |
| **Adverse Events or Intercurrent Illness** |  | **any time** | | | | | | | | | | |

a) In case of positive hepatitis B virus (HBV) serology (Anti-HBcAb positive) and negative PCR test at baseline (BL), the PCR test must be repeated at follow-up to exclude reactivation of HBV.

b) Predose, 30 min, 1 hr, 2 hr, 4 hr, 6 hr, 10 hr.

c) Predose, 30min, 1hr, 2hr, 4hr, 6hr. If the visit is an “Unscheduled

Visit” the post-dose sample needs to be taken only if the study treatment was taken.

d) Rectal biopsy up to Day −3 is acceptable. e) Prior to biopsy.

f) If available.

g) If missed on Day −1, the sample can be taken any day.

h) Pre-dose (.e.g., 24 hrs after the first study treatment administration or on Day 6).

i) 6 hr post-dose. If visit is an “Unscheduled Visit” the post-dose sample needs to be taken only if the study treatment was taken.

RO7486967**—F. Hoffmann-La Roche Ltd**

18/Protocol BP43099, Version 3

**Table 2 Schedule of Activities - Detailed Table**

| **Day**  Vers ion  2021-09-06 | **Schedule**  **Time (h)** | **ECG** | **Vital signs** | **Stool** | **Rectal biopsy** | **Clinical**  **Genotyping** | **PK Sample** | **ex-vivo cytokine release** | **Soluble PD biomarkers (serum / plasma)** | **Exploratory Safety Sample** |
| --- | --- | --- | --- | --- | --- | --- | --- | --- | --- | --- |
| **Screening** |  | x | x | x |  |  |  |  |  |  |
| **Day -1** |  |  | x | x | x | x |  |  | x | x |
| **Day 1** | **any time** |  |  | x |  |  |  |  |  | Any Time in Case of Liver Function Test Abnormalities (see section 8.2.8) |
| **Day 1** | **predose** | x | x |  |  |  | x | x |  |  |
| **Day 1** | **30 min ± 5 min** | x | x |  |  |  | x | x |  |  |
| **Day 1** | **1 hr ± 5 min** | x | x |  |  |  | x | x |  |  |
| **Day 1** | **2 hr ± 10 min** | x | x |  |  |  | x | x |  |  |
| **Day 1** | **4 hr ± 10 min** | x | x |  |  |  | x | x |  |  |
| **Day 1** | **6 hr ± 10** | x | x |  |  |  | x | x | x |  |
| **Day 1** | **10 hr ± 30 min** | x | x |  |  |  | x | x |  |  |
| **Day 2** | **any time** |  |  | x |  |  |  |  |  |  |
| **Day 2** | **predose** |  | x |  |  |  | x | x |  |  |
| **Day 2** | **6 hr ± 10** |  |  |  |  |  |  |  |  |  |
| **Day 3** | **any time** |  |  | x |  |  |  |  |  |  |
| **Day 3** | **predose** |  | x |  |  |  | x |  |  |  |
| **Day 4** | **any time** |  |  | x |  |  |  |  |  |  |
| **Day 4** | **predose** |  | x |  |  |  | x |  |  |  |
| **Day 5** | **any time** |  |  | x |  |  |  |  |  |  |
| **Day 5** | **predose** | x | x |  |  |  | x | x |  |  |
| **Day 5** | **30 min ± 5 min** | x | x |  |  |  | x | x |  |  |
| **Day 5** | **1 hr ± 5 min** | x | x |  |  |  | x | x |  |  |
| **Day 5** | **2 hr ± 10 min** | x | x |  |  |  | x | x |  |  |
| **Day 5** | **4 hr ± 10 min** | x | x |  |  |  | x | x |  |  |
| **Day 5** | **6 hr ± 10** | x | x |  |  |  | x | x | x |  |
| **Day 5** | **10 hr ± 30 min** | x | x |  |  |  | x | x |  |  |
| **Day 6** | **any time** |  |  | x |  |  |  |  |  |  |
| **Day 6** | **predose** |  | x |  |  |  | x | x |  |  |
| **Day 7** | **predose** | x | x | x | x |  | x | x |  |  |
| **Day 7** | **6 hr ± 10** |  |  |  |  |  |  |  | x |  |
| **Unscheduled visit** |  | x | x | x |  |  | x | x | x |  |
| **Early Withdrawal** Assessments as deemed feasible |  | x | x | x | x |  | x | x | x |  |
| **FU** |  | x | x | x |  |  | x | x | x |  |

Note: The sequence of assessments is: 1. ECG, 2. vital signs, 3. blood draws, 4. biopsies (for details see Section 8.1.2).

**2. INTRODUCTION**

**2.1 STUDY RATIONALE**

Inflammasomes are large multimeric protein complexes that actively regulate immunity and homeostasis through sensing and responding to microbial or other danger signals. The nucleotide-binding oligomerization domain-like receptor family pyrin 3 (NLRP3) inflammasome is expressed in immune cells of the gut and promotes inflammation through caspase-1-dependent cleavage and activation of immature pro-inflammatory cytokines interleukin (IL)-1β and IL-18 as well as by induction of pyroptotic cell death (de Zoete et al. 2014). Locally, IL-1β can induce cytokine production, enhance T-cell

activation and antigen recognition, and direct neutrophils to the site of injury. NLRP3 and IL-1β are both upregulated in active ulcerative colitis (UC; Tourkochristou et al. 2019). Several animal colitis model studies reported that inhibition of IL-1β function by methods that operate downstream of the NLRP3 inflammasome, resulting in reduced gut inflammation (Perera et al. 2018; Neudecker et al. 2017).

RO7486967 is a selective and reversible small molecule NLRP3 inflammasome inhibitor that has been shown to inhibit IL-1β release and cell death in vitro (see the RO7486967

Investigator Brochure [IB]).

This study will assess the safety, tolerability, and pharmacokinetics (PK) of RO7486967 and pharmacodynamic (PD) markers of inflammation in patients with moderate to severe active UC. The rationale for the study design is provided in Section 4.2.

**2.2 BACKGROUND**

**2.2.1 Ulcerative Colitis**

Ulcerative colitis is a chronic, relapsing disease characterized by diffuse mucosal inflammation of the colon. Moderate to severe UC is a debilitating disease that can result in hospitalization for disease flares, potential bowel perforation or hemorrhage, and increased risk of colorectal cancer. Current treatment goals include induction and maintenance of remission, improved quality of life, reduction in the need for long-term corticosteroids, and minimization of cancer risk. Management of UC includes initial use

of aminosalicylates (e.g., sulfasalazine or mesalamine) and/or corticosteroids to induce remission, or the use of biologic agents (e.g., tumor necrosis factor [TNF] inhibitors or integrin inhibitors) in patients with severe disease or in whom induction cannot be achieved with steroids. Once induction of remission has been achieved, maintenance therapy such as aminosalicylates, thiopurines (e.g., azathioprine or 6-mercaptopurine), or biologics (e.g., anti-TNF or anti-integrins monoclonal antibodies) are given with the aim of maintaining disease control and avoiding long-term steroid use. Patients with severe acute disease or severe refractory disease may be considered for surgery (e.g., colectomy).

Among multiple new molecular entities tested in patients with UC over the past two

anti-IL-12p40 (ustekinumab), and a pan-Jak inhibitor (tofacitinib) have demonstrated efficacy in patients with moderate to severe UC (Chang and Hudesman 2020).

Despite these recent additions to the treatment arsenal, over 70% of patients with moderate to severe UC still lack an effective, long-term therapy (Neurath 2017). Therefore, there is an urgent need for new treatments for UC in patients who do not respond to current treatment options.

**2.2.2 RO7486967**

The results of the entry-into-human (EIH) Phase 1 study IZD334-001 showed that RO7486967 appeared to be safe and well tolerated when administered to healthy male and female adult volunteers at single-ascending doses of 20 mg, 60 mg (fasted and fed),

180 mg, 450 mg, and 600 mg; and multiple-ascending doses (MAD) of 180 mg once daily (QD), 180 mg twice daily, and 450 mg QD for 7 days. The PK results showed rapid absorption of RO7486967, a half-life of approximately 7 hours, and no indication of a change in the exposure on repeat dosing for 7 days. The PD results showed that RO7486967 achieved the desired inhibition of IL-1β ex vivo whole blood lipopolysaccharide (LPS) and nigericin-induced IL-1β release assay.

A detailed description of the chemistry, pharmacology, and safety of RO7486967 is provided in the IB.

**2.3 BENEFIT/RISK ASSESSMENT**

Study BP43099 is the first study involving dosing of RO7486967 in participants with active UC to assess safety, the PK and PD effects at both the blood and gut tissue levels. A potential therapeutic effect on active UC will be limited as there is only 1 week of treatment duration. Safety data in healthy volunteers (HVs) from the EIH Study IZD334-001 are available and are summarized in the IB. Potential risks that had been identified based on clinical experience in HVs and non-clinical pharmacology and toxicology data in the relevant animal species will be closely monitored in this study.

The eligibility criteria, design and procedures adopted are considered to be appropriate for the safe conduct of the planned study. Participants will be closely monitored for safety (consistent with standard practices) and under close medical observation during the study.

The dose tested (i.e., 450 mg QD for 7 days) was the highest dose assessed in

Study IZD334-001 and was well tolerated.

Potential risks and plans for mitigation are detailed below.

• Hepatotoxicity: A battery of in vitro tests was conducted to assess the hepatotoxicity potential of RO7486967 and did not show mitochondrial toxicity,

on these in vitro assessments, the potential of RO7486967 to cause direct hepatotoxicity is considered low at therapeutically relevant concentrations. Consistently, no signs of hepatotoxicity were observed in the 26-week GLP study in the rat and 39-week GLP study in the monkey. During the MAD part of Study IZD334-001, 4 out of 18 healthy volunteers who received RO7486967 experienced a mild transaminase increase however there was no clear dose dependency and these abnormalities deemed not clinically significant by the Investigator. As a precautionary measure participants with impaired liver function will be excluded from enrollment, index of liver or biliary injury will be closely monitored during study. The management rules for abnormalities in liver function tests are detailed in the protocol (see Section 8.2.8).

• Infection: The NLRP3 inflammasome is a key component of the innate immune system that induces IL-1β production and cell death. NLRP3 is activated by a wide variety of signals (Coll et al. 2016). Blocking NLRP3 by RO7486967 will inhibit a single pathway among the several IL-1β production regulating mechanisms. Therefore, IL-1β production could still occur during infection when NLRP3 is inhibited. Consequently, blocking NLRP3 is expected to have a minimal immunomodulatory effect during infections. No evidence of bacterial, viral, fungal, and other opportunistic infections was found in rats and monkeys included in the nonclinical toxicology studies and in HVs in the Phase I clinical

study with RO7486967. As precautionary measures**,** patients with a known active

infection will be excluded from the study, and new/emerging infections will be closely monitored (see Section 8.2.8).

• Impaired immunization to vaccinations: many synthetic adjuvants activate NLRP3 in myeloid cells of the innate immune system, which promotes robust Th1 and

B-cell responses to vaccine antigens. Therefore, the inhibition of NLRP3 may affect the adaptive immune response produced by defined combination vaccine adjuvants (Seydoux et al. 2018). Therefore, patients who require vaccinations should complete any immunizations at least 4 weeks before first dose.

• The risks due to blood collection and rectal biopsy are regarded as minimal.

Participants might experience minor discomfort during the rectoscopy. Very rarely, rectal bleeding, pain, or infection after rectoscopy might occur.

An assessment was conducted to determine whether there is any impact of the

COVID-19 pandemic on the benefit/risk assessment of this study protocol, including (but not limited to) the patient population under study and study treatment being evaluated. On the basis of literature data (Shah A. 2020), SARS-CoV-2 viroporin protein 3a activates the NLRP3 inflammasome leading to the production of inflammatory cytokines resulting in tissue inflammation during respiratory illness caused by SARS-CoV-2 infection. NLRP3 inhibition may be beneficial during SARS-CoV-2 infection; however, there are no data available to support this hypothesis.

Considering the early stage of development of this study and the limited number of participants, a conservative approach has been adopted. Patients with symptomatic infections (including COVID-19) at screening will be excluded. Participants enrolled in this study will be closely monitored for new/emerging infections. Specific guidelines are provided in Section 8.2.8. In addition, vaccinations (including SARS-CoV-2/ COVID-19 vaccination if available) would need to be completed at least 4 weeks before first dose (Tartey and Thirumala-Devi 2019).

More detailed information about the known and expected benefits in the context of potential risks and reasonably expected adverse events (AEs) of RO7486967 is provided in the IB. Due to the short treatment duration and the nature of UC, there is no expected therapeutic benefit to the participants of Study BP43099.

**3. OBJECTIVES AND ENDPOINTS**

The objectives and corresponding endpoints are provided in Table 3.

**Table 3 Objectives and Endpoints**

|  | **Objectives** | **Endpoints** |
| --- | --- | --- |
| **Primary** | • To assess the safety and tolerability of orally administered RO7486967 in participants with active UC.  • To investigate the PK of orally administered RO7486967 in blood of participants with active UC. | • Incidence and severity of adverse events.  • Changes in vital signs, ECG parameters, and clinical laboratory safety parameters.  • Pharmacokinetic parameters of RO7486967 (and its metabolites, as applicable) in  blood by NCA and/or population  PK analysis. |
| **Secondary** | • To evaluate the effects on inflammation markers. | • Changes in CRP in blood and calprotectin in stool. |
| **Exploratory** | • To assess changes in PD markers in tissue and in stool in response to RO7486967.  • To assess changes in cellular compositions and gene expression in rectal tissue.  • To assess the inhibition of cytokine release after lipopolysaccharide (LPS) stimulation ex vivo in blood of patients treated with RO7486967.  • To explore the association of the microbiome composition with PD and safety of RO7486967.  • To assess the concentration of RO7486967 and its metabolites, as applicable, administered orally in rectal tissue of participants with active UC. | • **Rectal Tissue**: PK and PD biomarker such as mature IL-1β, cleaved caspase-1, cleaved gasdermin D, and cellular differences and function of individual cells.  • **Stool**: PD biomarkers such as mature IL-1β, microbiome composition.  • **Blood**: Cytokines (e.g., IL-1β and IL-18), ex vivo cytokine release (e.g., IL-1β). |

**4. STUDY DESIGN**

**4.1 OVERALL DESIGN**

Study BP43099 is a randomized, placebo-controlled, Investigator- and patient-blind, Sponsor-open study to assess safety, tolerability, and PK of RO7486967 in patients with active, moderate to severe UC. The study will utilize two treatment arms: placebo versus

450 mg RO7486967 administered orally QD.

A total of 18 participants are expected to be recruited, with approximately 12 receiving

450 mg RO7486967 and approximately 6 receiving placebo.

All participants in the study will have been diagnosed with UC previously and be in an active stage of their disease.

After the screening period, participants will be randomized to receive either placebo or RO7486967 orally (3 capsules of 150 mg). Randomization will be 2:1 (RO7486967 to placebo) and occur on Day −1. Treatment duration will be 7 days. Participants will have biopsies taken from the rectum (rectoscopy) between Day −3 and Day −1 and on Day 7 pre-dose (see Figure 1).

Participants will be hospitalized from Day −1 to Day 7. They will return to the clinic for the follow-up visit on Day 14 ± 3 days.

Dose adjustments are not foreseen during the study.

**4.1.1 Length of the Study**

The study length will be approximately 6 weeks (from screening through to follow-up), for each enrolled participant, as follows:

Screening: Up to 28 days. Treatment period: Days 1 to 7.

Follow-up: 7 (± 3) days after last study treatment administration.

**4.1.2 Stopping Rules Criteria**

The study will be put on hold pending a full safety review by the Sponsor, if any of the following occurs:

• A minimum of three Grade 3 serious AEs (SAEs) in three participants (at least one in each) considered to be related to RO7486967.

• At least one SAE ≥ Grade 4 (see NCI CTCAE v5.0), considered to be related to

RO7486967.

• Other clinically significant events (e.g., AEs or laboratory abnormalities) that in the opinion of the Investigator and the Sponsor preclude continued dosing.

• Decision by the Sponsor.

If moderate or severe AEs/SAEs are consistently observed across participants, or in case of unacceptable pharmacological effects that are reasonably attributable in the opinion of the Investigator to study treatment, then the dosing could be temporarily halted, with no further participants dosed until completion of a full safety review of the study. Relevant reporting and discussion with the Medical Monitor, relevant site personnel, and the Institutional Review Boards (IRB)/Independent Ethics Committees (IEC) would take place before dosing resumed. In the event of such an AE, every effort will be made to obtain a contemporaneous blood sample for PK analysis.

**4.1.3 Individual Stopping Criteria**

Dosing will be discontinued in an individual participant in case of:

• AST or ALT elevation of ≥ Grade 2 considered to be related to RO7486967.

• Serious and/ or severe, medically significant RO7486967-related AEs.

• Infection with SARS-CoV-2.

**4.1.4 Communication Strategy**

The Sponsor and Investigators will be in regular contact throughout the study by email/telephone/fax, as per normal interactions during the conduct of a clinical study. The Sponsor will arrange regular teleconferences and meetings to discuss study status.

The Sponsor will be available 24 hours per day to discuss any medical or study-related issues that may arise during the conduct of this study.

**4.2 SCIENTIFIC RATIONALE FOR STUDY DESIGN**

The study rationale is provided in Section 2.1.

**4.2.1 Rationale for Study Population**

Ulcerative colitis is a chronic disease. Despite the introduction of novel therapies and treatment strategies, many patients develop acute, severe episodes with high unmet medical need (Sedano et al. 2019). RO7486967 targets a new mechanism of action, which could be beneficial in the treatment of patients with UC (see also Section 2.1).

This study will recruit male and female participants who have active, moderate to severe

UC according to standard criteria and based on guidelines from Health Authorities.

UC patients presenting a mild renal impairment defined as creatinine clearance > 60 mL/minute are eligible to enter the study, supported by PBPK predictions: the impact on

systemic exposure is expected to be minimal, with an estimated increase of 10 % in the

AUC and no effect on Cmax.

**4.2.2 Rationale for Hospitalization**

PK behavior of RO7486967 in patients with UC is not known. In order to achieve a more optimized PK profile under controlled conditions, e.g., fasting conditions, participants will be hospitalized for the duration of the study.

**4.2.3 Rationale for Control Group**

RO7486967 will be compared against a placebo-concurrent control, with participants randomized in a 2:1 ratio to QD, oral administrations of RO7486967 or placebo, respectively. A placebo arm will be employed in order to derive a preliminary

assessment of the safety risk of RO7486967 in participants with UC and for equipoise in discriminating drug-relatedness of AEs. A placebo group will similarly inform the analysis of key biomarkers, including histopathologic changes in colonic biopsy samples and biomarkers used to establish PD effect.

**4.2.4 Rationale for Biomarker Assessments**

The main objectives of the mandatory biomarker strategy are to:

• Correlate drug exposure to rectal tissue target engagement (such as but not limited to caspase-1 cleavage) and downstream PD markers (such as, but not limited to, mature IL-1β, which may also be assessed in stool).

• Determine the impact of RO7486967 on inflammation markers (such as, but not limited to, C-reactive protein [CRP] in the blood and calprotectin in stool).

• Confirm and further investigate the level of inhibition of IL-1β production (through ex vivo cytokine release after LPS stimulation) in blood of patients.

Additional objectives include the assessment of changes in cellular composition (such as, but not limited to, neutrophils and inflammatory monocytes) and gene expression (such as, but not limited to, TNF, IL12B, CXCL10 inflammatory cytokines) upon RO7486967 treatment in rectal biopsies. Furthermore, the association of the microbiome composition (which may be assessed in stool or in the tissue) with PD and safety of RO7486967 will be explored. These data are of potential value for guidance in future patient stratification purposes or for treatment monitoring.

Serum and plasma samples will be collected to measure cytokines or other soluble biomarkers mechanistically linked to the mode of action of RO7486967. The impact of RO7486967 on these readouts will be explored.

In addition, safety exploratory biomarkers will be analyzed only in case signs of hepatotoxicity (such as transaminase increase) emerge during study.

Genotyping data may be used to explore whether the genotype may influence the target engagement or PD effects, or may affect the safety of RO7486967.

**4.3 JUSTIFICATION FOR DOSE**

The dose of 450 mg RO7486967 administered QD has been selected for this study based on PK, PD, and safety data from administration of single and multiple doses of RO7486967 in the completed SAD and MAD study in HVs. The dose of 450 mg was well tolerated when administered QD under fasted conditions for up to 7 consecutive days in study IZD334-001. This dose produced a robust PD effect by reducing the production of IL-1β in ex vivo LPS/nigericin stimulated whole blood by 99% up to 10 hours postdose and 90% over the dosing interval (24 hours), as compared with those of baseline. Therefore, this dose level was chosen for Study BP43099, as it is expected that it will provide a detectable PD readout also at the gut tissue level.

Further details are provided in the IB.

**4.4 END OF STUDY DEFINITION**

A participant is considered to have completed the study when the last scheduled procedure shown in the schedule of assessments has been completed.

The end of the study is defined as the date when the last participant last visit (LPLV) occurs. LPLV is expected to occur approximately 2 weeks after the last participant is randomized.

**5. STUDY POPULATION**

The study population rationale is provided in Section 4.2.1.

Participants of this study are male and female patients of 18 to 75 years of age

(inclusive), with active, moderate to severe UC.

**5.1 INCLUSION CRITERIA**

Participants are eligible to be included in the study only if all of the following criteria apply.

**Informed Consent**

1. Able and willing to provide written informed consent and to comply with the study protocol according to International Conference on Harmonisation (ICH) and local regulations. A signed Informed Consent Form must be available from the participant

before starting any study-specific assessments.

**Age**

2. Between 18 to 75 years of age (inclusive), at the time of signing the informed consent.

**Type of Participants and Disease Characteristics**

3. Diagnosis of UC at least 12 weeks prior to screening.

4. Patients with active moderate to severe UC as measured by partial MCS ≥ 3 and ≤

8, with stool frequency subscore ≥ 1, rectal bleeding subscore ≥ 1, and fecal calprotectin ≥ 150 μg/g.

5. Most recent colonoscopy within the preceding 3 years at the time of screening.

6. Screening colonoscopy for colorectal cancer conducted within the prior two years if:

• History of pancolitis and disease duration ≥ 8 years, or

• History of left-sided colitis and disease duration ≥ 12 years.

**Weight**

7. Body mass index (BMI) within the range of 18-35 kg/m2 (inclusive).

**Sex and Contraceptive/Barrier Replacement**

8. Male and female participants

The reliability of sexual abstinence for female enrollment eligibility needs to be evaluated in relation to the duration of the clinical study and the preferred and usual lifestyle of the participant. Periodic abstinence (e.g., calendar, ovulation, symptothermal, or post-ovulation methods) and withdrawal are not acceptable methods of preventing drug exposure.

a) Female participants

A female participant is eligible to participate if she is not pregnant, not breastfeeding, and at least one of the following conditions applies:

• Women of non-childbearing potential (WONCBP—i.e., post-menopausal;

pre-menarchal; or pre-menopausal with documented hysterectomy, bilateral salpingectomy, or bilateral oophorectomy).

• WOCBP (a woman is considered fertile following menarche and until becoming post-menopausal unless permanently sterile), who:

o Agree to remain abstinent (refrain from heterosexual intercourse) or use at least 1 acceptable contraceptive methods (see Appendix 5) for at least

1 month before dosing, during the treatment period and for at least 7 days after the final dose of RO7486967/placebo.

The following are acceptable contraceptive methods: bilateral tubal occlusion, male sterilization, established proper use of hormonal contraceptives that inhibit ovulation, hormone-releasing intrauterine devices and copper intrauterine devices, male or female condom with or without spermicide; and cap, diaphragm, or sponge with spermicide (see Appendix 5).

b) Male participants

No contraceptive requirements for male participants.

**5.2 EXCLUSION CRITERIA**

Participants are excluded from the study if any of the following criteria apply:

**Medical Conditions**

1. Diagnosis of fulminant UC, Crohn’s disease*,* indeterminate colitis, microscopic colitis, segmental colitis associated with diverticulosis, ischemic colitis, or radiation- induced colitis based on medical history, endoscopy, and/or histological findings.

2. Active infections requiring systemic therapy with antibiotic, antiviral, or antifungal medication or febrile illness within 7 days before Day −1.

3. History of primary or acquired immunodeficiency.

4. History of chronic pulmonary disease with resultant clinically significant abnormal pulmonary function.

5. History of clinically significant cardiac or cardiovascular disease or uncontrolled hypertension.

6. Presence of chronic liver disease.

7. Evidence of colonic dysplasia that cannot be completely removed.

8. Women: Pregnant or lactating.

9. Any condition or disease detected during the medical interview/physical examination that would render the patient unsuitable for the study, place the patient at undue

risk, or interfere with the ability of the patient to complete the study in the opinion of

the Investigator.

10. History of tuberculosis or a positive Quantiferon® Gold test.

11. History of clinically significant severe drug allergies, multiple drug allergies, or allergy to any constituent of the investigational medicinal product (IMP).

12. Lymphoma, leukemia, or any malignancy within the past 10 years, except for basal cell or squamous epithelial carcinomas of the skin that have been resected with no evidence of metastatic disease for 3 years and in situ carcinoma of the cervix that was completely removed surgically.

13. History or presence of clinically significant ECG abnormalities before study drug administration (e.g., PQ/PR interval ≥ 220 ms, QT corrected for heart rate using Fridericia’s correction factor (QTcF), < 350 or ≥ 450 ms) or clinically significant cardiovascular disease (e.g., cardiac insufficiency, coronary artery disease, cardiomyopathy, congestive heart failure, family history of congenital long QT interval syndrome, family history of sudden death).

14. Fecal microbiota transplant, defined as receipt of any product derived from the feces of another human and administered per oral, per nasogastric or nasoduodenal, or per rectum within the last 6 months.

15. Bowel surgery within 12 months prior to study start.

16. History of colectomy or partial colectomy.

17. History of known bleeding disorder or diseases with an increased risk of bleeding tendency.

**Prior/Concomitant Therapy**

18. Use of calcineurin inhibitors (e.g., tacrolimus, cyclosporine), vedolizumab, ustekinumab, anti-TNFα therapeutic or any other immune system−targeted therapy within 12 weeks or 5 half-lives, whichever is longer, prior to screening.

19. Rectal therapy with 5- aminosalicylic acid (ASA) or corticosteroids within 2 weeks of screening.

20. Leukocyte apheresis within 12 weeks of screening.

21. Vaccine(s) within four weeks prior to first dose, or plans to receive vaccines during the study or within 28 days of the last dose, with the exception of SARS-CoV-2 vaccination, which could be given 14 days after last dose.

**Prior/Concurrent Clinical Study Experience**

22. Donation of blood or blood products in excess of 500 mL within 3 months.

23. Exposure to more than 4 investigational treatments within 12 months prior to Day 1.

24. Use of any investigational drug or any other type of interventional medical research within 12 weeks of screening.

**Diagnostic Assessments**

25. Abnormal hematologic values:

• Anemia (hemoglobin < 10 g/dL)

• Leukocytosis (white blood cells ≥ 2 × ULN)

• Neutropenia < 1000/μL

• Thrombocytopenia (platelet count < 100,000/μL)

• Thrombocytosis (platelet count ≥ 2 × ULN)

• Eosinophilia (eosinophil count ≥ 2 × ULN)

26. Renal function: creatinine clearance < 60 mL/minute.

27. Abnormal hepatic enzyme or hepatic function values:

• ALT, AST, alkaline phosphatase (ALP), total bilirubin or gamma-glutamyl transferase (GGT) ≥ 1.5 × ULN (unless total bilirubin ≥ 1.5 × ULN is caused by Gilbert’s disease).

• International normalized ratio (INR) > ULN.

• Albumin < 3 g/dL.

28. Cardiac troponin I outside the normal range.

29. Positive HIV antibody test.

30. Presence of hepatitis B surface antigen (HBsAg). If the patient has positive total hepatitis B core antibody (HBcAb), a negative PCR test for hepatitis B DNA

(< 20 IU/mL) is required for the patient to be eligible.

31. Positive hepatitis C by PCR test result at screening or within the 3 months prior to starting study treatment.

**Other Exclusions**

32. History of regular alcohol consumption within 2 months of screening defined as: An average weekly intake of > 14 drinks for men or > 7 drinks for women. One drink is equivalent to 12 g of alcohol: 12 ounces (360 mL) of beer, 5 ounces (150 mL) of wine, or 1.5 ounces (45 mL) of 80 proof distilled spirits.

33. Any suspicion or history of alcohol abuse and/or suspicion of regular consumption of drug of abuse.

34. Patients under judicial supervision, guardianship, or curatorship.

35. Patients dependent on the Sponsor, the participating clinic(s) or the Investigator(s).

**5.3 LIFESTYLE CONSIDERATIONS**

**5.3.1 Meals and Dietary Restrictions**

Participants will receive standard meals while in the clinic.

Participants will be administered the study treatment once a day in the morning after an overnight fast of at least 8 hours prior to dosing. Water should be restricted for 1 hour predose and 1 hour postdose, and no food is allowed until at least 4 hours postdose.

**5.3.2 Alcohol and Tobacco**

Consumption of alcohol is not allowed 48 hours before the screening visit, prior to the admission at the clinic and the follow-up visit. It is not permitted during the study treatment period at the clinic. During the period when the participant is not at the study center (i.e., between Day 8 and 12) alcohol consumption must be no more than an maximum of 2 units per day (1 unit is equivalent to 330mL of beer, 125 mL of wine or 25 mL of spirits).

The use of tobacco products will not be permitted during the periods of study center residency.

**5.3.3 Activity**

Participants must refrain from strenuous exercise throughout the study.

**5.4 SCREEN FAILURES**

Screen failures are defined as participants who consent to participate in the study but are not subsequently entered in the study. Screen failures may be tracked separately.

The Investigator will maintain a screening log to record details of all participants screened and to confirm eligibility or record reasons for screening failure.

Individuals who do not meet the criteria for participation in this study (screen failure) may be re-screened a total of 3 times if the reason for failure is considered to be transient.

Re-screened participants should be assigned the same participant number as for the initial screening.

For re-screened participants, the following specific criteria must be met:

• Testing for HBV, HIV, HCV, and latent tuberculosis infection need not be repeated if these were already done within 6 months of first screening and the results were negative.

• WOCBP must repeat pregnancy testing.

• Screening laboratory evaluations beyond those itemized above must be completed within 28 days of the first dosing.

Samples of screen failures will not be retained for further analysis.

**6. TREATMENTS**

Study treatment is defined as any IMP (including placebo) or marketed product intended to be administered to a study participant according to the study protocol.

The IMPs for this study are RO7486967 and placebo. All IMPs required for completion of this study will be provided by the Sponsor. Study treatment administration will be at the study center under supervision of site staff.

Cases of overdose, medication error, drug abuse, or drug misuse, along with any associated AEs, should be reported as described in Appendix 2, Section 5.2.

**6.1 TREATMENTS ADMINISTERED**

Table 4 summarizes the treatments administered. Guidelines for treatment discontinuation are provided in Section 7.

Please see the IB for more details.

**Table 4 Summary of Treatments Administered**

| **Study Treatment**  **Name:** | RO7486967 | Placebo |
| --- | --- | --- |
| **IMP and NIMP** | IMP | IMP |
| **Dose Formulation:** | Capsule | Capsule |
| **Unit Dose Strength** | 150 mg | N/A |
| **Dose:** | 450 mg | N/A |
| **Route of**  **Administration:** | Oral | |
| **Sourcing:** | Provided centrally by the Sponsor | |
| **Packaging and**  **Labeling:** | Study treatment will be provided in a container. Each container will be labeled as required per country requirement. | |

**6.2 PREPARATION/HANDLING/STORAGE/ACCOUNTABILITY**

Study drug packaging will be overseen by the Sponsor’s clinical study supplies department and bear a label with the identification required by local law, the protocol number, drug identification, and dosage.

The packaging and labeling of the study medication will be in accordance with the

Sponsor’s standard and local regulations.

The study site should follow all instructions included with each shipment of IMP. The investigational site will acknowledge receipt of IMPs and confirm the shipment condition and content. Any damaged shipments will be replaced. The Investigator or designee

must confirm that appropriate temperature conditions have been maintained during transit for all IMPs received and that any discrepancies have been reported and resolved before use of the IMPs. All IMPs must be stored in a secure, environmentally controlled, and monitored (manual or automated) area in accordance with the labeled storage conditions, with access limited to the Investigator and authorized staff.

Only participants enrolled in the study may receive IMPs, and only authorized staff may supply or administer IMPs.

The study site (i.e., Investigator or other authorized personnel [e.g., pharmacist]) is responsible for maintaining records of IMP delivery to the site, IMP inventory at the site, IMP use by each participant, and disposition or return of unused IMP, thus enabling reconciliation of all IMP received, and for ensuring that participants are provided with doses specified by the protocol. Upon arrival of the IMPs at the site, site personnel will complete the following:

• Check the IMPs for damage.

• Verify proper identity, quantity, integrity of seals, and temperature conditions.

• Report any deviations or product complaints to the Study Monitor upon discovery.

The Investigator or delegate will hand out the correct medication to the participants each day to be taken under supervision. Treatment should be taken every day at approximately the same time.

The Investigator or delegate must confirm appropriate temperature conditions have been maintained during transit for all study treatment received and any discrepancies are reported and resolved before use of the study treatment.

All study treatments must be stored in a secure, environmentally controlled, and monitored (manual or automated) area in accordance with the labeled storage conditions with access limited to the Investigator and authorized site staff.

The Investigator is responsible for study treatment accountability, reconciliation, and record maintenance (i.e., receipt, reconciliation and final disposition records).

IMPs will either be disposed of at the study site according to the study site’s institutional standard operating procedure (SOP) or returned to the Sponsor with the appropriate documentation. The site's method of IMP destruction must be agreed upon by the Sponsor. Local or institutional regulations may require immediate destruction of used IMP for safety reasons. The site must obtain written authorization from the Sponsor before any IMP is destroyed, and IMP destruction must be documented on the appropriate form. Accurate records of all IMPs received at, dispensed from, returned to, and disposed of by the study site should be recorded on the drug accountability log.

Refer to the IB for information on IMP formulation, IMP handling (including preparation and storage), and accountability.

**6.3 MEASURES TO MINIMIZE BIAS: RANDOMIZATION AND BLINDING**

**6.3.1 Method of Treatment Assignment**

Randomization will occur once all eligibility criteria have been satisfied. No stratification will be applied.

All participants will be centrally assigned to randomized study treatment using an interactive (voice/web) response system (IxRS) system. Before the study is initiated, the telephone number and call-in directions for the IxRS and/or the login information and directions for the IxRS will be provided to each site.

The randomization numbers will be generated by the Sponsor or its designee. The randomization list will be made available to the individual responsible for PK/PD sample bioanalysis, and to statisticians or programmers at Roche. PK/PD data can be received and cleaned on an ongoing basis.

Study treatment will be administered at the study visits indicated in the SoA (Section 1.3).

**6.3.2 Blinding**

BP43099 will be a randomized, placebo-controlled, Investigator- and patient-blind, Sponsor-open study.

If unblinding is necessary for participant management (in the case of an SAE), the Investigator will be able to break the treatment code by contacting the IxRS. Treatment codes should not be broken except in emergencies. If the Investigator wishes to know the identity of the study treatment for any other reason, he/she should contact the Medical Monitor directly. The Investigator should document and provide an explanation for any premature unblinding (e.g., accidental unblinding, unblinding due to an SAE).

This is an Investigator- and patient-blind but Sponsor-open study. The randomization schedule will not be made available to members of the core study team. The randomization schedule may be made available to some members of the study team on the basis of operational need—e.g., pharmacometrician. The names and

responsibilities of all those to whom the randomization schedule is released will be documented in the electronic trial master file, along with the date of release. As per Health Authority reporting requirements, the Sponsor will break the treatment code for all unexpected SAEs that are considered by the Investigator to be related to study treatment.

**6.4 TREATMENT COMPLIANCE**

The qualified individual responsible for dispensing the study treatment will prepare the correct dose according to the randomization schedule. This individual will write the date dispensed and participant number on the study treatment container label and on the Drug Accountability Record. This individual will also record the study treatment number received by each participant during the study and record the returned number of capsules on the Drug Accountability Record.

**6.5 CONCOMITANT THERAPY**

Any medication or vaccine (including over-the-counter [OTC] or prescription medicines, approved dietary and herbal supplements, nutritional supplements) used by a participant from 4 weeks prior to screening until the follow-up visit must be recorded along with reason for use, dates of administration (including start and end dates) and dosage information (including dose and frequency).

The Medical Monitor should be contacted if there are any questions regarding concomitant or prior therapy.

All concomitant medications should be reported to the Investigator and recorded on the

Concomitant Medications electronic Case Report Form (eCRF).

All medication administered to manage AEs should be recorded on the Adverse Event eCRF.

**6.5.1 Permitted Therapy**

All concomitant medications throughout the duration of the study should be recorded in the eCRF. Use of the following therapies will be permitted, as specified below:

• Paracetamol is allowed up to a maximum dose of 2 g/day.

• Participants who use the following therapies prior to screening and are on a stable regimen should continue their use:

o Oral contraceptives, hormone-replacement therapy, or other maintenance therapy.

• Participants who are taking non-biologic standard of care (SOC) therapy for UC during the study will remain on their SOC therapy. Specific instructions for each SOC is provided below:

o Stable therapy for at least 2 weeks prior to screening with:

– oral 5-ASA, and/or

– oral glucocorticoid (not to exceed 20 mg/day prednisone equivalent).

o Stable therapy for at least 8 weeks with:

– azathioprine, or

– 6-mercaptopurine, or

– methotrexate (and folic acid)

**6.5.2 Prohibited Therapy**

All medications (prescription and OTC) taken within 4 weeks of study screening will be recorded on the appropriate eCRF.

As a general rule, no concomitant medication will be permitted, with the exception of medications to treat AEs and those specified as permitted therapies, unless the rationale for exception is discussed and clearly documented between the Investigator and the Sponsor.

Vaccines within four weeks prior to first dose, or plans to receive vaccines during the study, or within 28 days of the last dose, will not be permitted with the exception of SARS-CoV-2 vaccines which would be permitted at least 14 days after the last dose of RO7486967/placebo.

Use of the following therapies will be prohibited during the study and for at least 14 days or at least 5 half-lives prior to initiation of study treatment, whichever is longer unless otherwise specified below:

• Any rectal therapy.

• Doses of glucocorticoids exceeding 20 mg/day of prednisone or equivalent.

• Non-steroidal anti-inflammatory medications (e.g., ibuprofen, naproxen) due to a propensity for these treatments to worsen inflammation in UC.

• Calcineurin inhibitors (e.g., tacrolimus, cyclosporine) and any immune system targeted therapy within 12 weeks or 5 half-lives, whichever is longer, before the start of screening.

• Antibiotics, antifungals, antivirals throughout treatment duration.

Additionally, in vitro studies have shown that RO7486967 is a potential substrate for P- glycoprotein (P-gP) and Breast Cancer Resistance Protein (BCRP) transporters. P- glycoprotein (P-gp) inhibitors such as amiodarone, carvedilol, clarithromycin, dronedarone, itraconazole, lapatinib, lopinavir and ritonavir, propafenone, quinidine, ranolazine, ritonavir, saquinavir and ritonavir, telaprevir, tipranavir and ritonavir, verapamil, and BCRP inhibitors like cyclosporine A will be prohibited 7 days prior to study treatment initiation and throughout the study.

P-gp inducers such as rifampin, carbamazepine, herbal medicine like St John’s wort and food product like green tea and curcumin, will not be allowed from 14 days prior to study treatment initiation and throughout the study.

**6.6 DOSE MODIFICATION**

No dose modification is foreseen during the study.

**6.7 TREATMENT AFTER THE END OF THE STUDY**

The Sponsor does not intend to provide RO7486967 or other study interventions to participants after conclusion of the study or any earlier participant withdrawal.

**7. DISCONTINUATION OF STUDY, STUDY TREATMENT AND PARTICIPANT DISCONTINUATION/WITHDRAWAL**

An excessive rate of withdrawals (either participants discontinuing study treatment or withdrawing from the study) can render the study non-interpretable. Therefore, unnecessary withdrawal of participants should be avoided and efforts should be taken to motivate participants to comply with all the study-specific procedures as outlined in this protocol.

Details on study and site closures are provided in Appendix 1 Regulatory, Ethical, and

Study Oversight Considerations.

**7.1 DISCONTINUATION OF STUDY TREATMENT**

For data to be collected at the time of treatment discontinuation and follow-up and for any further evaluations that need to be completed see the SoA (Section 1.3).

Reasons for discontinuation of study treatment (or withdrawal from the study) may include, but are not limited to, the following:

• Participant withdrawal of consent at any time.

• Any medical condition that the Investigator or Sponsor determines may jeopardize the participant’s safety if he or she continues in the study.

• Investigator or Sponsor determination that treatment discontinuation is in the best interest of the participant.

• Pregnancy.

• Any event that meets stopping criteria as defined in Section 4.1.3.

If a clinically significant finding is identified (including, but not limited to, changes from baseline in QT interval corrected using Fridericia’s formula [QTcF]) after enrollment, the Investigator or qualified designee will determine if the participant can continue in the study and if any change in participant management is needed. The review of the ECG printed at the time of collection must be documented. Any new clinically relevant finding should be reported as an AE.

Every effort should be made to obtain information on participants who withdraw from the study but have not withdrawn consent. Participants who discontinue study treatment prematurely will have a study completion/early withdrawal visit (see Section 8.8.3),

unless the participant withdrew consent. The primary reason for premature study treatment discontinuation should be documented on the appropriate eCRF. Participants who discontinue study treatment prematurely may be replaced (see Section 7.2).

**7.2 PARTICIPANT DISCONTINUATION/WITHDRAWAL FROM THE STUDY**

Participants have the right to voluntarily withdraw from the study at any time for any reason.

If possible, information on reason for withdrawal from the study should be obtained. The primary reason for withdrawal from the study should be documented on the appropriate eCRF. Participants will not be followed for any reason after consent has been withdrawn. This includes the follow-up assessments.

When a participant voluntarily withdraws from the study, or is withdrawn by the Investigator, samples collected until the date of withdrawal will be analyzed, unless the participant specifically requests for these to be discarded or local laws require their immediate destruction. However, if samples have been tested prior to withdrawal, results from those tests will be used as part of the overall research data. A participant's withdrawal from this study does not, by itself, constitute withdrawal of samples donated

to the Research Biosample Repository (RBR).

Participants who withdraw from the study for safety reasons will not be replaced. Participants who withdraw from the study for other reasons and have not received any dose may be replaced. Patients withdrawing during the study for non-safety reasons will be replaced.

For data to be collected at the time of study discontinuation and for any further evaluations that need to be completed, see the SoAs (Section 1.3).

**7.3 LOST TO FOLLOW-UP**

A participant will be considered lost to follow-up if the participant repeatedly fails to return for scheduled visits and is unable to be contacted by the study site.

The following actions must be taken if a participant fails to return to the clinic for a required study visit:

• The site must attempt to contact the participant and reschedule the missed visit as soon as possible, counsel the participant on the importance of maintaining the assigned visit schedule, and ascertain whether or not the participant wishes to (and/or should) continue in the study.

• Before a participant is deemed lost to follow-up, the Investigator or designee must make every effort to regain contact with the participant. These contact attempts should be documented in the participant’s medical record.

• Should the participant continue to be unreachable, he/she will be considered to have withdrawn from the study.

Discontinuation of sites or of study as a whole are handled as part of Appendix 1.

**8. STUDY ASSESSMENTS AND PROCEDURES**

Study procedures and their timepoints are summarized in the SoAs (Section 1.3). Protocol waivers or exemptions are not allowed.

Immediate safety concerns should be discussed with the Sponsor immediately upon occurrence or awareness to determine if the participant should continue or discontinue study treatment.

Procedures conducted as part of the participant’s routine clinical management

(e.g., blood count) and obtained before signing of the Informed Consent Form (ICF) may be utilized for screening or baseline purposes provided the procedure met the

protocol-specified criteria and was performed within the time-frame defined in the SoAs.

Samples for laboratory tests will be sent to the local laboratory, to one or several central laboratories, or to the Sponsor for analysis depending on sample type and anticipated analysis. Instruction manuals and supply kits will be provided for all central laboratory assessments.

Repeat or unscheduled samples may be taken for safety reasons or for technical issues with the samples.

On the basis of continuous analysis of the data in this study, any assessment, sample type, or biomarker evaluation not considered to be critical for safety may be stopped at any time if the data from the assessments or samples collected do not produce useful information.

**8.1 SAFETY ASSESSMENTS**

Planned timepoints for all safety assessments are provided in the SoAs (Section 1.3).

Safety assessments will consist of monitoring and recording AEs, including SAEs and non-serious adverse events of special interest (NSAESI); measurement of

protocol-specified safety laboratory assessments; measurement of protocol-specified vital signs, ECGs; and other protocol-specified tests that are deemed critical to the safety evaluation of the study.

**8.1.1 Physical Examinations**

A complete physical examination will include, at a minimum, assessments of the cardiovascular, respiratory, gastrointestinal, dermatological, neurological, and musculoskeletal systems; in addition to head; eyes; ears; nose; throat; neck; and lymph

node systems. Height and weight will also be measured and recorded. Further examination of other body systems may be performed at the Investigator’s discretion.

A complete physical examination will be performed at the timepoints specified in the SoA (Section 1.3) by trained medical personnel at the study center.

The BMI will be calculated. Height will be recorded at screening only. Body weight will be recorded at screening and at all subsequent physical examinations and as clinically indicated.

Any abnormality identified at or before baseline should be recorded on the General

Medical History and Baseline Conditions eCRF.

Changes from baseline abnormalities should be recorded in participant’s notes. New or worsened clinically significant abnormalities should be recorded as AEs on the Adverse Event eCRF.

**8.1.2 Vital Signs**

Blood pressure (BP), pulse rate, body temperature (tympanic or oral), and respiratory rate will be assessed at the timepoints specified in the SoA (Section 1.3).

BP and pulse measurements will be assessed in a supine position with a completely automated device. Manual techniques will be used only if an automated device is not available. When possible, the same arm should be used for all BP measurements.

BP and pulse measurements should be preceded by at least 5 minutes of rest for the participant in a quiet setting without distractions (e.g., television, cell phones).

Three readings of BP and pulse will be taken. The first reading should be rejected. The second and third readings should be averaged to give the measurement to be recorded in the eCRF.

Single measurements of the other vital signs will be taken before blood collection for laboratory tests but after ECG collection when scheduled at the same timepoint. Biopsies should be taken last.

The timings of assessments may be amended or the number of assessments increased during study conduct on the basis of emerging data in order to allow for optimal characterization of the effect profile.

**8.1.3 Electrocardiograms**

Triplicate 12-lead ECG will be obtained as outlined in the SoA (see Section 1.3) using an ECG machine. Measurements of PR, QRS complex (QRS), QT, and QTc intervals may be performed by the instrument or calculated using an external tool.

At each timepoint at which triplicate ECGs are required, three individual ECG tracings should be obtained as closely as possible in succession but no more than 5 minutes apart. The full set of triplicates should be completed in less than 10 minutes.

To minimize variability, it is important that participants be in a resting position for

≥ 10 minutes prior to each ECG evaluation. Body position should be consistently maintained for each ECG evaluation to prevent changes in heart rate. Environmental distractions (e.g., television, radio, conversation) should be avoided during the pre-ECG resting period and during ECG recording. ECGs should be performed prior to any scheduled vital sign measurements and blood draws. In some cases, it may be appropriate to repeat abnormal ECGs to rule out improper lead placement as contributing to the ECG abnormality.

For safety monitoring purposes, the Investigator or designee must review, sign, and date all ECG tracings. Paper or electronic copies will be kept as part of the participant’s permanent study file at the site. If considered appropriate by Roche, ECGs may be analyzed retrospectively at a central laboratory.

ECG characteristics (including heart rate, QRS duration, and PR, and QT intervals) will be recorded on the eCRF. QTcF (Fridericia’s correction) and RR will be calculated and

recorded on the eCRF. Changes in T‑wave and U‑wave morphology and overall ECG

interpretation will be documented on the eCRF. T-wave information will be captured as normal or abnormal, U-wave information will be captured in two categories: absent/normal or abnormal.

**8.1.4 Clinical Safety Laboratory Assessments**

Normal ranges for the study laboratory parameters must be supplied to the Sponsor before the study starts. A list of clinical laboratory tests to be performed is provided in Appendix 4, and these assessments must be conducted in accordance with the local laboratories process. If analysis is required to be done centrally, the samples and assays should be clearly identified and instructions may be provided by the central laboratory manual according to the requirements from the SoAs (Section 1.3).

The Investigator must review the laboratory report, document this review, and record any clinically relevant changes occurring during the study in the AE section of the eCRF. The laboratory reports must be filed with the source documents. Clinically significant

abnormal laboratory findings are those which are not associated with the underlying disease, unless judged by the Investigator to be more severe than expected for the participant's condition.

• In the event of unexplained abnormal clinically significant laboratory test values, the tests should be repeated immediately and followed up until they have returned to

the normal range and/or an adequate explanation of the abnormality is found.

• If such values do not return to normal/baseline within a period judged reasonable by the Investigator, the etiology should be identified and the Sponsor notified.

• If laboratory values from non-protocol-specified laboratory assessments performed at the local laboratory require a change in participant management or are considered clinically significant by the Investigator (e.g., SAE or AE or

dose-modification) then, the results must be recorded in the eCRF/Clinical Trial

Adverse Event/Special Situations Form.

Results of clinical laboratory testing will be recorded on the eCRF or be received as electronically produced laboratory reports submitted directly from the local or central laboratory.

Additional blood or urine samples may be taken at the discretion of the Investigator if the results of any test fall outside the reference ranges, or clinical symptoms necessitate additional testing to monitor participant safety.

Where the clinical significance of abnormal laboratory results at screening is considered uncertain, screening lab tests may be repeated before randomization to confirm eligibility.

If there is an alternative explanation for a positive urine or blood test for drugs of abuse (e.g., previous occasional intake of a medication or food containing, for example, codeine, benzodiazepines, or opiates), the test could be repeated to confirm washout.

Based on continuous analysis of the data in this study and other studies, any sample type not considered to be critical for safety may be stopped at any time if the data from the samples collected do not produce useful information.

**8.1.5 Safety Biomarker Assessments**

Safety exploratory biomarkers such as GLDH, osteopontin, MCSFR-1 and CK18 (including fragmented CK18 and full length CK18) will be analyzed only in case signs of hepatotoxicity (such as transaminase increase) emerge during the study. GLDH may provide supporting or confirmatory evidence of hepatotoxicity. Biomarkers such as osteopontin and M-CSFR-1 may reflect immune mediated mechanisms while CK18 may shed light on mechanism of cell death.

**8.1.6 Medical History and Demographic Data**

Medical history includes clinically significant diseases (including UC, prior therapies for UC and respective treatment outcome and procedures), surgeries, cancer history (including prior cancer therapies and procedures), reproductive status, smoking history, use of alcohol and drugs of abuse, and all medications (e.g., prescription drugs, OTC drugs, herbal or homeopathic remedies, nutritional supplements) used by the participant within 4 weeks prior to the screening visit. All treatment history for UC preceding the study should be captured as completely as possible.

Demographic data will include age, sex, and self‑reported race/ethnicity.

**8.2 ADVERSE EVENTS AND SERIOUS ADVERSE EVENTS**

The definitions of an AE or SAE can be found in Appendix 2. The NSAESI and

disease-related events and/or disease-related outcomes not qualifying as AEs or SAEs are discussed in Sections 8.2.6 and 8.2.7.

The Investigator and any qualified designees are responsible for ensuring that all AEs (including assessment of seriousness, severity and causality; see Appendix 2) are recorded on the Adverse Event eCRF and reported to the Sponsor in accordance with instructions provided in this section and in Appendix 2.

Procedures used for recording AEs are provided in Appendix 3:

• Diagnosis versus signs and symptoms:

**–** Other AEs

• AEs occurring secondary to other events

• Persistent or recurrent AEs

• Abnormal laboratory values

• Abnormal vital sign values

• Abnormal liver function tests

• Deaths

• Preexisting medical conditions

• Lack of efficacy or worsening of the condition being studied

• Hospitalization or prolonged hospitalization

• Patient-reported outcome data

**8.2.1 Time Period and Frequency for Collecting Adverse Event and**

**Serious Adverse Event Information**

The method of recording, evaluating, and assessing causality of AEs and SAEs and the procedures for completing and transmitting SAE reports are provided in Appendix 2.

Investigators will seek information on AEs at each participant’s contact. All AEs, whether reported by the participant or noted by study personnel, will be recorded in the participant’s medical record and on the Adverse Event eCRF as follows:

**After informed consent** has been obtained **but prior to initiation of study treatment**, only SAEs caused by a protocol-mandated intervention should be reported (e.g., SAEs related to invasive procedures such as biopsies). Any other AE should not be reported.

**After initiation of study treatment**, all AEs, regardless of relationship to study treatment, will be reported until 7 days after the final dose of study treatment.

**Post-study AEs and SAEs:** The Investigator is not required to actively monitor participants for AEs after the end of the AE reporting period, defined as 7 days after the final dose of study treatment or until the start of another treatment, whichever comes first.

However, if the Investigator learns of any SAE (including a death) or other AEs of concern that are believed to be related to prior treatment with study treatment, at any time after a participant has been discharged from the study, and the Investigator considers the event to be reasonably related to the study treatment or study participation, the Investigator must promptly notify the Sponsor. For the procedure of reporting, see Appendix 2.

**8.2.2 Method of Detecting Adverse Events and Serious Adverse**

**Events**

Care will be taken not to introduce bias when detecting AEs and/or SAEs. Open-ended and non-leading verbal questioning of the participant is the preferred method to inquire about AE occurrence.

A consistent methodology of non-directive questioning should be adopted for eliciting

Adverse Event information at all participant-evaluation timepoints.

**8.2.3 Follow-Up of Adverse Events and Serious Adverse Events**

**8.2.3.1 Investigator Follow-Up**

The Investigator should follow each AE until the event has resolved to baseline grade or better, the event is assessed as stable by the Investigator, the event is otherwise explained, the participant is lost to follow-up (Section 7.3), or the participant withdraws consent. Every effort should be made to follow all SAEs considered to be related to study treatment or study-related procedures until a final outcome can be reported.

During the study period, resolution of AEs (with dates) should be documented on the Adverse Event eCRF and in the participant’s medical record to facilitate source data verification. If, after follow-up, return to baseline status or stabilization cannot be established, an explanation should be recorded on the Adverse Event eCRF.

All pregnancies reported during the study should be followed until pregnancy outcome and reported according to the instructions provided in Section 8.2.5.

**8.2.3.2 Sponsor Follow-Up**

For SAEs, NSAESIs, and pregnancies, the Sponsor or a designee may follow up by telephone, fax, electronic mail, and/or a monitoring visit to obtain additional event details and outcome information (e.g., from hospital discharge summaries, consultant reports,

autopsy reports) in order to perform an independent medical assessment of the reported event.

**8.2.4 Regulatory Reporting Requirements for Serious Adverse**

**Events**

Prompt notification by the Investigator to the Sponsor of an SAE regardless of relationship to study drug is essential so that legal obligations and ethical responsibilities towards the safety of participants and the safety of a study treatment under clinical investigation are met.

The Sponsor has a legal responsibility to notify both the local regulatory authority and other regulatory agencies about the safety of a study treatment under clinical investigation as per local requirements. The Sponsor will comply with country-specific regulatory requirements relating to safety reporting to the regulatory authority, IRB/IEC, and Investigators.

Investigator safety reports must be prepared for suspected unexpected serious adverse reactions according to local regulatory requirements and Sponsor policy and forwarded to Investigators as necessary.

An Investigator who receives an Investigator safety report describing an SAE or other specific safety information (e.g., summary or listing of SAEs) from the Sponsor will review and then, file it along with the IB and will notify the IRB/IEC, if appropriate according to local requirements.

For immediate (i.e., without undue delay) and expedited reporting requirements from Investigator to Sponsor and from Sponsor to Health Authority, Investigators, IRB and EC, see Appendix 2, Section 5.

**8.2.4.1 Emergency Medical Contacts**

To ensure the safety of study participants, access to the Medical Monitors is available

24 hours a day, 7 days a week. Details will be available separately.

**8.2.5 Pregnancy**

Female participants of childbearing potential will be instructed to immediately inform the Investigator if they become pregnant during the study or within 7 days after the final dose of study treatment.

If a pregnancy is reported, the Investigator should inform the Sponsor within 24 hours of learning of the pregnancy and should follow the pregnancy reporting process as detailed in Appendix 5. All pregnancies reported during the study should be followed up until pregnancy outcome. Follow-up information on the infant will be collected according to procedures outlined in Appendix 5.

Abnormal pregnancy outcomes (e.g., spontaneous abortion, fetal death, stillbirth, congenital anomalies, and/or ectopic pregnancy) are considered SAEs (Appendix 5).

**8.2.6 Non-Serious Adverse Events of Special Interest**

NSAESI are required to be reported by the Investigator to the Sponsor immediately (i.e.,

*without undue delay*; see Appendix 2 for reporting instructions). NSAESI for this study include the following:

• Cases of an elevated ALT or AST in combination with either an elevated bilirubin or

clinical jaundice, as defined in Table 5 and Appendix 3.

• Suspected transmission of an infectious agent by the study treatment, as defined below:

Any organism, virus, or infectious particle (e.g., prion protein transmitting transmissible spongiform encephalopathy), pathogenic or non-pathogenic, is considered an infectious agent. A transmission of an infectious agent may be suspected from clinical symptoms or laboratory findings that indicate an infection in a participant exposed to a medicinal product. This term applies only when a contamination of the study treatment is suspected.

**8.2.7 Disease-Related Events and/or Disease-Related Outcomes Not**

**Qualifying as Adverse Events or Serious Adverse Events**

Disease-related events and outcomes are not applicable for this study.

**8.2.8 Management of Specific Adverse Events**

Guidelines for management of specific adverse events are provided in Table 5, and

Table 6.

**Table 5 Guidelines for Managing Liver Function Test Abnormalities**

| **Abnormality* Action to Be Taken** |
| --- |
| Grade 1 AST or ALT • Continue study treatment  elevation or • Collect a sample for exploratory safety biomarkers  AST/ALT > ULN to  ≤ 3 × ULN • Monitor LFT (i.e., AST, ALT, ALP, TBL) every 3 days until  values resolve to normal or baseline value. |
| Grade 2 AST or ALT • Discontinue study treatment  elevation or • Monitor LFT (i.e., AST, ALT, ALP, TBL) every 48-72 hours  AST/ALT > 3 to ≤ 5 × ULN until values resolve to normal or baseline value.  • Collect a sample for exploratory safety biomarkers  • Events > 5 days duration:  o Obtain hepatology consultation; evaluate etiology. |
| Grade 3 AST or ALT • Discontinue study treatment  elevation or • Monitor LFT (i.e., AST, ALT, ALP, TBL) every 24-48 hours  AST/ALT > 5 to ≤ 20 × ULN until decreasing and then follow every 48-72 hours  • Collect a sample for exploratory safety biomarkers  • Events > 5 days duration:  o Obtain hepatology consultation; evaluate etiology. |
| Grade 4 AST or ALT • Discontinue study treatment elevation or  AST/ALT > 20 × ULN • Follow management guidelines as described for Grade 3  event. |
| AST or ALT ≥ 3 × ULN in • Consider event an AE of special interest (potential Hy’s law combination with total case) and report *immediately, without undue delay* (see bilirubin ≥ 2 × ULN or Appendix 3, Section 6).  clinical jaundice as defined  by Hy’s law • Collect a sample for exploratory safety biomarkers  • Permanently discontinue study treatment.  • Repeat LFT (i.e., AST, ALT, ALP, TBL) every 24-48 hours to confirm the abnormalities and to determine if they are increasing or decreasing and then follow every 48-72 hours.  • Monitor participant closely for clinical signs and symptoms  • Events > 5 days duration:  o Obtain hepatology consultation; evaluate etiology. |

AE = adverse event; ALP = alkaline phosphatase; ALT = alanine transaminase; AST = aspartate transaminase; LFT = liver function test; NCI CTCAE = National Cancer Institute Common Terminology Criteria for Adverse Events; TBL = total bilirubin; ULN = upper limit of normal.

*AE grading based on NCI CTCAE (v5.0).

**Table 6 Guidelines for Managing Infections**

| **Event Action to Be Taken** |
| --- |
| Grade 1 infection • Continue study treatment.  • Closely monitor the evolution of the event until resolution.  • Treatment as per local guidelines. |
| Grade 2 infection • Confirm infection:  oObtain complete blood count with differential.  o Obtain necessary tests or cultures and identify pathogen (if possible).  o Additional work-ups should be based on clinical suspicion and local standard of care.  • Discontinue study treatment.  • Closely monitor the evolution of the event until resolution.  • Oral intervention indicated (e.g., antibiotic, antifungal, antiviral)  according to local treatment guidelines. |
| Grade 3 and • Confirm infection:  Grade 4 infection o Obtain complete blood count with differential.  o Obtain necessary tests or cultures and identify pathogen (if possible).  o Additional work-ups should be based on clinical suspicion and local standard of care.  • Discontinue study treatment.  • Close monitor the evolution of the event until resolution.  • Intravenous intervention indicated (e.g., antibiotic, antifungal, antiviral) according the local treatment guidelines. |
| Confirmed SARS- • Discontinue study treatment.  CoV-2 infection • Closely monitor the evolution of the event until resolution. (any grade) • Treatment as per local guidelines, depending on severity. |

**8.3 TREATMENT OF OVERDOSE**

Study treatment overdose is the accidental administration of a drug in a quantity that is higher than the assigned dose. An overdose or incorrect administration of study treatment is not an AE unless it results in untoward medical effects (see Sections 5 and

5.2 of Appendix 2 for further details).

Decisions regarding dose-interruptions or modifications (if applicable) will be made by the Investigator in consultation with the Medical Monitor based on the clinical evaluation of the participant.

In the event of an overdose, the Investigator should:

1. Contact the Sponsor’s Medical Monitor immediately.

2. Closely monitor the participant for AE/SAE and laboratory abnormalities until resolved.

3. Obtain an additional blood sample for PK analysis if requested by the Medical

Monitor (determined on a case-by-case basis).

4. Document the quantity of the excess dose, as well as the duration of the overdose, in the eCRF.

Any dose of RO7486967 greater than 450 mg within a day will be considered an overdose.

There is no specific treatment available in case of an overdose.

**8.4 PHARMACOKINETICS**

Blood samples will be collected to determine plasma concentration of RO7486967 and its metabolites, as appropriate. Samples will be collected at the timepoints specified in the SoAs (Section 1.3).

The timings of sample collection may be amended during study conduct on the basis of emerging data in order to allow optimal characterization of the PK profile.

Plasma concentrations of RO7486967 (and its metabolites as applicable) will be measured by a specific and validated LC-MS/MS method. Leftover samples may be used for exploratory analyses and may also be used for assay development/validation experiments.

Samples collected from placebo-treated participants will not be analyzed in the first instance, but retained for subsequent analysis if appropriate.

Samples will be destroyed within 2 years after the date of final clinical study report

(CSR).

**8.4.1 Gut Tissue Concentration**

Gut tissue for the measurement of the concentration of RO7486967 and its metabolites as applicable will be collected from rectal biopsies at timepoints specified in the SoAs (Section 1.3).

Concentrations of RO7486967 and its metabolites as applicable will be measured by a specific and qualified LC-MS/MS method. One biopsy per timepoint will be used for the assessment (see Section 8.6.1.3).

**8.5 PHARMACODYNAMICS AND BIOMARKERS ANALYSES**

**8.5.1 Genetic and Genomic Analyses**

**8.5.1.1 Clinical Genotyping**

The DNA may be used for the analysis of, but is not limited to:

• Genetic variants of the NLRP3 gene and genes encoding proteins involved in

NLRP3 inflammasome pathway.

• Genes associated with the metabolism of RO7486967.

• Genetic variants for which genetic polymorphisms have been previously associated with autoimmune disease, e.g., but not limited to inflammatory bowel disease.

To identify these variants and polymorphisms, genome-wide methods may be used. Given the complexity and exploratory nature of genome-wide analyses, genomics data

and analyses will not be shared with Investigators or study participants unless required

by law. Participants will not be identified by name or any other personally identifying information. Data arising from all biosamples, including samples for analyses of inherited human DNA, will be subject to the confidentiality standards described in the sample documentation and in in Section 1.4, Appendix 1.

**Transcriptome Analysis**

Rectal tissue biopsy will be collected as outlined in the SoAs (Section 1.3) for RNA

extraction and subsequent gene expression profiling to enable:

• Identification of PD biomarkers.

• Assessment of treatment response (PD).

**Metagenomics Analysis**

Rectal tissue biopsy and stool will be collected as specified in the SoA (Section 1.3). RNA or DNA may be extracted for subsequent metagenomic analysis.

**8.6 PHARMACODYNAMICS AND BIOMARKER SAMPLES**

Additionally, samples may be used for research purposes to identify biomarkers useful for predicting and monitoring response to RO7486967, identifying biomarkers useful for predicting and monitoring RO7486967 safety, assessing PD effects of RO7486967, and investigating mechanism of therapy resistance. Additional markers may be measured in the case that a strong scientific rationale develops.

Samples should be collected as specified in the SoA (Section 1.3).

Based on continuous analysis of the data in this study and other studies, any sample type and/or analysis not considered to be critical for safety may be stopped at any time if the data from the samples collected does not produce useful information.

Unless otherwise specified below, samples (including blood, tissue, stool, extracts, derivatives, etc.) will be destroyed no later than 5 years after the date of final CSR. For participants who consent to RBR, leftover samples will be transferred to RBR

(Section 8.7).

Any remaining blood, serum, plasma, stool, tissue, and/or the isolated derivatives from samples after the specified analyses may also be used for additional exploratory biomarker profiling or/and (assay) validation experiments. Samples may be used for research to develop methods, assays, prognostics, and/or companion diagnostics related to NLRP3 pathway, mode of action of RO7486967, and disease understanding for UC.

Details on processes for collection and shipment of these samples can be found in separate sample documentation.

**8.6.1 Mandatory Samples**

The samples detailed below for PD and biomarker research are required and will be collected from all participants.

**8.6.1.1 Blood Sampling**

Blood samples will be collected for determining inhibition of IL-1β cytokine release following ex vivo LPS stimulation, cytokines levels (such as but not limited to IL-1β), and CRP level.

A mandatory whole blood sample will be taken for DNA extraction and clinical genotyping from every participant. If the sample is missed on Day 1, it can be collected at any other scheduled visit*.*

Finally, blood samples will be collected for safety exploratory biomarker assessment at baseline and In case of liver function test abnormalities (see Section 8.2.8).

**8.6.1.2 Stool Sampling**

Stool samples will be collected for determining the levels of calprotectin and may be used to assess microbiome composition and levels of cytokines or PD biomarkers (such as but not limited to IL-1β).

Volatile biomarkers may be assessed from the stool samples as well.

**8.6.1.3 Tissue Sampling**

**Rectal Biopsies**

In addition to the biopsy mentioned in Section 8.4.1 for PK assessment in tissue, additional biopsies will be collected for PD assessments as indicated in the SoAs (see Table 1).

In order to have the highest number of samples available for the most important analyses, the sequence of analysis will correspond to:

1. PD biomarker (e.g., mature IL-1β, cleaved caspase-1, etc.),

2. Hematoxylin and eosin, and immunohistochemistry,

3. Tissue PK,

4. PD biomarker (e.g., cleaved IL-18 and cleaved gasdermin D),

5. Assessment of cellular differences and function of individual cells,

6. Exploratory and/or disease biomarkers.

Biopsies should be taken from the rectal mucosa up to 15 cm from the anal verge. Rectal biopsies will be collected for the following assessments:

• PD biomarkers such as cleaved caspase-1 and mature IL-1β. Additional PD

biomarkers (such as, but not limited to, cleaved IL-18 and cleaved gasdermin D)

may be assessed.

• To characterize cell types such as but not limited to neutrophils and inflammatory monocytes. Cell types of interest may be identified through various methods such as but not limited to histology (hematoxylin and eosin staining), immunohistochemistry (specific protein staining) and fluorescence in situ hybridization (detection of gene transcripts expression).

• RNA extraction on tissue colon biopsy samples may be performed in order to undertake whole or targeted transcriptome gene expression analysis. Analysis may be performed at the single cell level.

• Bacterial DNA or RNA may be extracted for subsequent mucosa-associated metagenomic analysis*.*

**8.7 SAMPLES FOR RESEARCH BIOSAMPLE REPOSITORY**

**8.7.1 Overview of the Research Biosample Repository**

The Roche RBR is a centrally administered group of facilities for the long-term storage of human biologic samples, including body fluids, solid tissues, and derivatives thereof

(e.g., DNA, RNA, proteins, peptides). The collection, storage, and analysis of the RBR

samples will facilitate the rational design of new pharmaceutical agents and the development of diagnostic tests, which may allow for individualized drug therapy for patients in the future.

Samples for the RBR will be collected from participants who give specific consent to participate in this optional RBR. Collected RBR samples will be used to achieve the following objectives:

• To study the association of biomarkers with efficacy or progressive disease.

• To identify safety biomarkers that are associated with susceptibility to developing

AEs or can lead to improved AE monitoring or investigation.

• To increase knowledge and understanding of disease biology and drug safety.

• To study treatment response, including drug effects and the processes of drug absorption and disposition.

• To develop biomarker or diagnostic assays and establish the performance characteristics of these assays.

**8.7.2 Sample Collection**

The following samples will be stored in the RBR and used for research purposes, including, but not limited to, research on biomarkers related to RO7486967, diseases, or drug safety:

• Leftover plasma samples

• Leftover serum samples

• Leftover tissue samples and the isolated derivatives (e.g., DNA)

• Leftover blood samples and the isolated derivatives (e.g., DNA)

• Left over stool samples and isolated derivatives (e.g., DNA)

Samples may be sent to one or more laboratories for analysis of germline or somatic mutations via whole genome sequencing (WGS)/whole exome sequencing (WES), or other genomic analysis methods. Genomics is increasingly informing researchers’ understanding of disease pathobiology. WGS and WES provide a comprehensive characterization of the genome and exome, respectively, and, along with clinical data collected in this study, may increase the opportunity for developing new therapeutic approaches or new methods for monitoring efficacy and safety or predicting which patients are more likely to respond to a drug or develop AEs.

Participants will not be identified by name or any other personally identifying information. Data generated from RBR samples will be analyzed in the context of this study but may also be explored in aggregate with data from other studies. The availability of a larger dataset will assist in identification and characterization of important biomarkers and pathways to support future drug development.

If allowed by local laws, the participant may request access to the non-interpreted genomics data (from blood samples). Such a request must be conveyed to the Sponsor by the Investigator, using the email address: [global.return-genomics-](mailto:global.return-genomics-results@roche.com)[results@roche.com.](mailto:results@roche.com) The information, if available at the time of the request, would be shared with the Investigator in the form of a data file of raw genomic sequencing data. The Sponsor will not provide any interpretation of this raw genomic sequencing data. The interpretation of these data will require expertise in genomic and genetic analysis and an understanding

of the variability of the technology used for data generation.

For all samples, dates of consent should be recorded on the associated RBR page of the eCRF. Details on processes for collection and shipment of these samples can be found in separate sample documentation.

RBR samples will be stored and used until no longer needed or until they are depleted. The RBR storage period will be in accordance with the IRB/EC‑approved Informed

Consent Form and applicable laws (e.g., Health Authority requirements).

The repository samples will be subject to the confidentiality standards (as described under Confidentiality and in Appendix 1).

**8.8 TIMING OF STUDY ASSESSMENTS**

**8.8.1 Screening and Pre-treatment Assessments**

Written informed consent for participation in the study must be obtained before performing any study-specific screening tests or evaluations. ICFs for enrolled participant and for participants who are not subsequently enrolled will be maintained at the study site.

All screening, and all pre-treatment assessments (related to entry criteria), must be completed and reviewed by the Investigator to confirm that participants meet all eligibility criteria. The Investigator will maintain a screening log to record details of all participants screened and to confirm eligibility or record reasons for screening failure.

An Eligibility Screening Form documenting the Investigator’s assessment of each screened participant with regard to the protocol’s inclusion and exclusion criteria is to be completed by the Investigator and kept at the investigational site.

Screening and pre-treatment assessments will be performed within 28 days prior to

Day 1, unless otherwise specified. Results of SOC tests or examinations performed prior to obtaining informed consent and within 28 days prior to Day 1 may be used and do not need to be repeated for screening.

In order to assess MCS patients will be asked to keep a diary for three consecutive days to record stool frequency and rectal bleeding.

**8.8.2 Assessments during Treatment**

Under no circumstances will participants who enroll in this study and have completed treatment as specified be permitted to be allocated a new randomization number and re-enroll in the study.

All assessments must be performed as per the SoAs (Section 1.3). Assessments scheduled on the day of study treatment administration should be performed prior to administration of study treatment, unless otherwise noted in the SoAs.

**8.8.3 Assessments at Study Completion/Early Withdrawal Visit**

Participants who complete the study (defined as completing the Day 7 visit) will be asked to return to the clinic for a follow-up visit at Day 14 (± 3 days). Participants who discontinue early will have an early withdrawal visit at the time of the withdrawal, and the follow-up visit 7 days (± 3 days) later.

**8.8.4 Follow-Up Assessments**

After the study completion/early withdrawal visit, AEs should be followed as outlined in

Sections 8.2.1 and 8.2.3.

**8.8.5 Assessments at Unscheduled Visits**

For activities that are required to be performed in case of an unscheduled visit, refer to the SoAs (Section 1.3).

**9. STATISTICAL CONSIDERATIONS**

**9.1 STATISTICAL HYPOTHESES**

No statistical hypotheses are to be tested in this study.

**9.2 SAMPLE SIZE DETERMINATION**

The study is expected to enroll approximately 18 patients in total, with 6 in the placebo arm and 12 in the treatment arm.

No formal statistical sample size calculations were performed; sample size is based on feasibility. No formal statistical analyses will be undertaken. Data will be summarized and listed as appropriate.

**9.3 POPULATIONS FOR ANALYSES**

For purposes of analysis, the following populations are defined in Table 7.

**Table 7 Analysis Populations**

**Population Description**

Intent-to-treat All randomized participants will be included in the intent-to-treat population.

Safety All participants randomized to study treatment and who received at least one dose of the study treatment, whether prematurely withdrawn from the study or not, will be included in the safety analysis.

Pharmacokinetic All participants who have received at least one dose of study treatment and who have data from at least one postdose sample will be included in the PK analysis population. Participants will be excluded from the PK analysis population if they significantly violate the inclusion or exclusion criteria, deviate significantly from the protocol, or if data are

unavailable or incomplete which may influence the PK

analysis. Excluded cases will be documented together with the reason for exclusion. All decisions on exclusions from the analysis will be made prior to database closure.

**9.4 STATISTICAL ANALYSES**

**9.4.1 Demographics and Baseline Characteristics**

Summaries for categorical baseline variables will include counts and frequencies. Summaries for continuous baseline variables will include counts, means, and standard deviations. Participants in the intent-to-treat analysis set will contribute to baseline summaries.

For each variable, the “baseline value” will be the last non-missing value obtained prior to the first administration of study treatment.

**9.4.2 Efficacy Analyses**

No efficacy analyses are planned.

**9.4.3 Safety Analyses**

All safety analyses (see Table 8) will be based on the safety analysis population grouped according to the treatment assigned at randomization.

**Table 8 Safety Statistical Analysis Methods**

**Endpoint Statistical Analysis Methods**

Adverse events The original terms recorded on the eCRF by the Investigator for AEs will be coded by the Sponsor.

AEs will be summarized by mapped term and appropriate thesaurus level. Incidence, nature and severity of AEs, number of participants with SAEs, treatment-related SAEs, SAEs leading to discontinuation, treatment-related AEs, or AEs leading to discontinuation will be tabulated and/or listed, as appropriate.

Clinical laboratory tests

All clinical laboratory data will be stored on the database in the units in which they were reported. Laboratory test values will be presented in International System of Units (SI units; Système International d’Unités) by individual outputs with flagging of abnormal results. In addition, tabular summaries will be used, as appropriate. Laboratory data not reported in SI units will be converted to SI units before processing.

Vital signs Vital signs data will be presented with flagging of values outside the normal ranges and flagging of abnormalities. In addition, tabular summaries will be used, as appropriate.

ECG data analysis ECG data will be tabulated and/or listed, as appropriate.

Concomitant medications

The original terms recorded on the participants’ eCRF by the Investigator

for concomitant medications will be standardized by the Sponsor by utilizing a mapped term and appropriate drug dictionary level.

Concomitant medications will be presented using appropriate outputs.

AE = adverse event; ECG = electrocardiogram; eCRF = electronic case report form; SAE =

serious adverse event.

**9.4.4 Pharmacokinetic Analyses**

Pharmacokinetic parameters will be read directly from the plasma concentration-time profiles or calculated by using standard non-compartmental methods. The following PK parameters will be computed for RO7486967 and its metabolites as applicable. However, other PK parameters might be computed in addition as appropriate:

• Time to maximum concentration (Tmax) after the dose on Day 1, and at steady-state.

• Maximum concentration (Cmax) measured post the dose on Day 1, and at steady- state.

• Concentration measured at the end of the dosing interval prior to the next study treatment administration (Ctrough), when applicable.

• Area under the serum concentration-time curve after the first dose administration extrapolated to infinity (AUCinf) and over the dosing interval (AUCtau) at steady-state (where tau=24 hours). If deemed useful, a partial AUC0-t may be used instead of AUCinf or AUCtau should extrapolation to infinity or up to tau not be considered reliable.

• Apparent clearance after the first dose administration (CL/F = dose/AUCinf) as well as at steady-state (CLss/F = dose/AUCtau).

• Apparent volume of distribution (V/F) after the first dose administration.

• Estimates of T1/2 after the first dose administration.

To evaluate drug accumulation, individual as well as mean (standard deviation) of apparent peak and trough (predose) after each dose administration will be plotted graphically over the course of assessments. In addition, AUCtau post the dose on Day 1, or alternatively a partial AUC0-t , will be tested statistically against AUCtau (or alternatively AUC0-t ) post the dose on the day of the final dose using an analysis of variance with terms in the model for participants and day. A natural log transform of the data will be done prior to the analysis.

Individual and mean plasma and gut tissue concentrations at each sampling time point for RO7486967 will be presented by listings and descriptive summary statistics including arithmetic means, geometric means, ranges, standard deviations, and coefficients of variation. Individual and mean plasma concentration versus time will be plotted on linear or semi-logarithmic scales as appropriate.

All PK parameters will be presented by individual listings and summary statistics including arithmetic means, geometric means, medians, ranges, standard deviations, and coefficients of variation.

In addition to the non-compartmental method, a population PK analysis using non-linear mixed effects modeling (NONMEM, Version 7.4 or higher) will be performed including pooled data from multiple studies to analyze the plasma concentration-time data of RO7486967 following oral administration. The analysis methods and results of the population PK modeling will be reported separately.

**9.4.5 Pharmacodynamic Analyses**

All PD parameters including but not limited to cleaved caspase-1 and mature IL-1β in the tissue, and IL-1β production inhibition following ex vivo LPS stimulation will be presented by listings and descriptive summary statistics separately, by visit and treatment group.

**9.4.6 Pharmacokinetic/Pharmacodynamic Relationships**

The PK/PD relationship between RO7486967 plasma concentrations and PD markers (including but not limited to IL-1β) may be characterized by exploratory analyses, including pooled data from other studies where RO7486967 is administered. The analysis methods and modeling results will be reported separately.

**9.5 SUMMARIES OF CONDUCT OF STUDY**

All protocol deviations will be listed. Data for study treatment administration and concomitant medication will be listed. The number of participants who were randomized, discontinued, and completed the study will be summarized and listed.

**10. REFERENCES**

Chang S, Hudesman D. First-line biologics or small molecules in inflammatory bowel disease: a practical guide for the clinician. Curr Gastroenterol Rep 2020;22:7.

Coll RC, O’Neill LAJ, Schroder K. Questions and controversies in innate immune research: what is the physiological role of NLRP3? Cell Death Discov

2016;2:16019.

de Zoete MR, Palm NW, Zhu S, et al. Inflammasomes. Cold Spring Harb Perspect Biol

2014;6:a016287.

Investigator’s Brochure RO7486967.

Neudecker V, Haneklaus M, Jensen O, et al. Myeloid-derived miR-223 regulates intestinal inflammation via repression of the NLRP3 inflammasome. J Exp Med

2017;214:1737-52.

Neurath MF. Current and emerging therapeutic targets for IBD. Nat Rev Gastroenterol

Hepatol 2017;14:269-78.

Perera AP, Fernando R, Shinde T, et al. MCC950, a specific small molecule inhibitor of NLRP3 inflammasome attenuates colonic inflammation in spontaneous colitis mice. Sci Rep 2018;8:8618.

Sedano R, Quera R, Simian D, et al. An approach to acute severe ulcerative colitis.

Expert Rev Gastroenterol Hepatol. 2019;13:943-55.

Seydoux E, Liang H, Dubois Cauwelaert N, et al. Effective combination adjuvants engage both TLR and inflammasome pathways to promote potent adaptive immune responses. J Immunol 2018;201:98-112.

Shah A. Novel Coronavirus-induced NLRP3 inflammasome activation: a potential drug target in the treatment of COVID-19. Front Immunol 2020;11:1021.

Tartey S, Thirumala-Devi K. Differential role of the NLRP3 inflammasome in infection and tumorigenesis. Immunology 2019;156:329–38.

Tourkochristou E, Aggeletopoulou I, Konstantakis C, et al. Role of NLRP3 inflammasome in inflammatory bowel diseases. World J Gastroenterol

2019;25:4796-804.

**11. SUPPORTING DOCUMENTATION AND OPERATIONAL CONSIDERATIONS**

• **Appendix 1** (Regulatory, Ethical, and Study Oversight Considerations),

• **Appendix 2** (AE: Definitions, Reporting; Procedures for Evaluating, Follow-up, and

Reporting)

• **Appendix 3** (Procedures of Recording Adverse Events),

• **Appendix 4** (Clinical Laboratory Tests),

• **Appendix 5** (Contraceptive Guidance and Collection of Pregnancy Information).

**Appendix 1**

**Regulatory, Ethical, and Study Oversight Considerations**

**1. REGULATORY AND ETHICAL CONSIDERATIONS**

**1.1. COMPLIANCE WITH LAWS AND REGULATIONS**

This study will be conducted in full conformance with the ICH E6 guideline for Good Clinical Practice and the principles of the Declaration of Helsinki, or the laws and regulations of the country in which the research is conducted, whichever affords the greater protection to the individual. The study will comply with the requirements of the ICH E2A guideline (Clinical Safety Data Management: Definitions and Standards for Expedited Reporting). Studies conducted in the United States or under a U.S. Investigational New Drug (IND) application will comply with U.S. FDA regulations and applicable local, state, and federal laws. Studies conducted in the EU/EEA will comply with the EU Clinical Trial Directive (2001/20/EC).

**1.2. INSTITUTIONAL REVIEW BOARD OR ETHICS COMMITTEE**

This protocol, the ICFs, any information to be given to the participant (e.g., advertisements, diaries, etc.), and relevant supporting information must be submitted to the IRB/EC by the Principal Investigator and reviewed and approved by the IRB/EC before the study is initiated.

The Principal Investigator is responsible for providing written summaries of the status of the study to the IRB/EC annually or more frequently in accordance with the

requirements, policies, and procedures established by the IRB/EC. Investigators are also responsible for promptly informing the IRB/EC of any protocol amendments

(Section 2.3.1, Appendix 1).

The Investigator should follow the requirements for reporting all s to the Sponsor. Investigators may receive written IND safety reports or other safety‑related

communications from the Sponsor. Investigators are responsible for ensuring that such reports are reviewed and processed in accordance with Health Authority requirements and the policies and procedures established by their IRB/EC, and archived in the site’s study file.

**1.3. INFORMED CONSENT**

The Sponsor’s Master Informed Consent Form (and ancillary sample ICFs such as a Child’s Assent or Caregiver's Informed Consent Form, if applicable) will be provided to each site. If applicable, it will be provided in a certified translation of the local language. Participants must be informed that their participation is voluntary. Participants will be required to sign a statement of informed consent that meets the requirements of 21 CFR

50, local regulations, ICH guidelines, Health Insurance Portability and Accountability Act requirements, where applicable, and the IRB/IEC or study center. The Sponsor or its designee must review and approve any proposed deviations from the Sponsor's sample

ICFs or any alternate consent forms proposed by the site (collectively, the “Consent

Forms”) before IRB/EC submission. The final IRB/EC‑approved Consent Forms must be

provided to the Sponsor for Health Authority submission purposes according to local requirements. Participants must be re-consented to the most current version of the ICF(s) during their participation in the study. A copy of the ICF(s) signed by all parties must be provided to the participant.

The ICFs must be signed and dated by the participant before his or her participation in the study. The case history or clinical records for each participant shall document the informed consent process and that written informed consent was obtained prior to participation in the study.

The ICFs should be revised whenever there are changes to study procedures or when new information becomes available that may affect the willingness of the participant to take part. The final revised IRB/EC-approved ICFs must be provided to the Sponsor for Health Authority submission purposes if required as per local regulations.

If the ICFs are revised (through an amendment or an addendum) while a participant is participating in the study, the participant may be re-consented by signing the most current version of the ICFs or the addendum, in accordance with applicable laws and IRB/EC policy. For any updated or revised ICFs, the case history or clinical records for each participant shall document the informed consent process and that written informed consent was obtained using the updated/revised ICFs for continued participation in the study. The study team will provide guidance for which participants need to re-consent in the event of an update to the ICF.

A copy of each signed ICF must be provided to the participant. All signed and dated ICFs must remain in each participant’s study file or in the site file and must be available for verification by study monitors at any time.

A participant who is re-screened is not required to sign another ICF if the re-screening occurs within 60 days from the previous ICF signature date.

**Consent to Participate in the Research Biosample Repository**

The ICF will contain a separate section that addresses participation in the RBR. The Investigator or authorized designee will explain to each participant the objectives, methods, and potential hazards of participation in the RBR. Participants will be told that they are free to refuse to participate and may withdraw their samples at any time and for any reason during the storage period. A separate, specific signature will be required to document a participant’s agreement to provide optional RBR samples. Participants who decline to participate will not provide a separate signature.

The Investigator should document whether or not the participant has given consent to participate by completing the RBR Sample Informed Consent eCRF.

In the event of death or loss of competence of a participant who is participating in the research, the participant's samples and data will continue to be used as part of the RBR.

**Approval by the Institutional Review Board or Ethics Committee** Collection, storage, and analysis of RBR samples is contingent upon the review and approval of the exploratory research and the RBR portion of the ICF by each site's IRB/EC and, if applicable, an appropriate regulatory body. If a site has not been granted approval for RBR sampling, this section of the protocol will not be applicable at that site

**Withdrawal from the Research Biosample Repository**

Participants who give consent to provide samples for the RBR have the right to withdraw their samples at any time for any reason. If a participant wishes to withdraw consent to the testing of his or her samples, the Investigator must inform the Medical Monitor and Site Monitor in writing of the participant’s wishes using the RBR Withdrawal Form and, if the study is ongoing, must enter the date of withdrawal on the RBR Withdrawal of Informed Consent eCRF. The participant will be provided with instructions on how to withdraw consent after the study is closed. A participant's withdrawal from Study BP43099 does not, by itself, constitute withdrawal of samples from the RBR. Likewise, a participant’s withdrawal from the RBR does not constitute withdrawal from Study BP43099. Data already generated before time of withdrawal of consent to RBR will still be used.

**1.4. CONFIDENTIALITY**

Participants will be assigned a unique identifier by the Sponsor. Any participant records or datasets that are transferred to the Sponsor will contain the identifier only; participant names, or any information which would make the participant identifiable, will not be transferred.

The participant must be informed that his/her personal study-related data will be used by the Sponsor in accordance with local data protection law. The level of disclosure must also be explained to the participant.

Medical information may be given to a participant’s personal physician or other appropriate medical personnel responsible for the participant’s welfare, for treatment purposes.

The participant must be informed that his/her medical records may be examined by Clinical Quality Assurance auditors or other authorized personnel appointed by the Sponsor, by appropriate IRB/IEC members, and by inspectors from regulatory authorities.

**Confidentiality for Research Biosample Repository**

Data generated from RBR samples must be available for inspection upon request by representatives of national and local Health Authorities, and Roche monitors, representatives, and collaborators, as appropriate.

Participant medical information associated with RBR samples is confidential and may only be disclosed to third parties as permitted by the ICF (or separate authorization for use and disclosure of personal health information) signed by the participant, unless permitted or required by law.

Data derived from RBR sample analysis on individual participants will generally not be provided to study Investigators unless a request for research use is granted. The aggregate results of any conducted research will be available in accordance with the effective Roche policy on study data publication.

Genetic research data and associated clinical data may be shared with researchers who are not participating in the study or submitted to government or other health research databases for broad sharing with other researchers. Participants will not be identified by name or any other personally identifying information. Given the complexity and exploratory nature of these analyses, genetic data and analyses will not be shared with Investigators or participants unless required by law.

Any inventions and resulting patents, improvements, and/or know-how originating from the use of the RBR sample data will become and remain the exclusive and unburdened property of the Sponsor, except where agreed otherwise.

**Monitoring and Oversight Research Biosample Repository**

Samples collected for the RBR will be tracked in a manner consistent with Good Clinical Practice by a quality-controlled, auditable, and appropriately validated laboratory information management system, to ensure compliance with data confidentiality as well as adherence to authorized use of samples as specified in this protocol and in the ICF. The Sponsor’s monitors and auditors will have direct access to appropriate parts of records relating to participant participation in RBR for the purposes of verifying the data provided to the Sponsor. The site will permit monitoring, audits, IRB/EC review, and Health Authority inspections by providing direct access to source data and documents related to the samples.

**1.5. FINANCIAL DISCLOSURE**

Investigators will provide the Sponsor with sufficient, accurate financial information in accordance with local regulations to allow the Sponsor to submit complete and accurate financial certification or disclosure statements to the appropriate Health Authorities. Investigators are responsible for providing information on financial interests during the course of the study and for one year after completion of the study (i.e., LPLV).

**2. DATA HANDLING AND RECORD**

**2.1. DATA COLLECTION AND MANAGEMENT RESPONSIBILITIES**

**2.1.1. Data Quality Assurance**

All participant data relating to the study will be recorded on printed or electronic CRF unless transmitted to the Sponsor or designee electronically (e.g., laboratory data). The Investigator is responsible for verifying that data entries are accurate and correct by physically or electronically signing the CRF.

The Investigator must maintain accurate documentation (source data) that supports the information entered in the CRF.

The Investigator must permit study-related monitoring, audits, IRB/IEC review, and regulatory agency inspections and provide direct access to source data documents.

The Sponsor or designee is responsible for the data management of this study including quality checking of the data.

Study monitors will perform ongoing source data verification to confirm that data entered into the CRF by authorized site personnel are accurate, complete, and verifiable from source documents; that the safety and rights of participants are being protected; and that the study is being conducted in accordance with the currently approved protocol and any other study agreements, ICH Good Clinical Practice (GCP), and all applicable regulatory requirements.

**2.1.2. Source Data Records**

Source documents (paper or electronic) are those in which participant data are recorded and documented for the first time. They include, but are not limited to, hospital records, clinical and office charts, laboratory notes, memoranda, COAs (paper or electronic), evaluation checklists, pharmacy dispensing records, recorded data from automated instruments, copies of transcriptions that are certified after verification as being accurate and complete, microfiche, photographic negatives, microfilm or magnetic media, x-rays,

participant files, and records kept at pharmacies, laboratories, and medico‑technical

departments involved in a clinical study.

Before study initiation, data to be entered directly into the eCRFs (i.e., no prior written or electronic record of the data) and considered source data must be defined in the Trial Monitoring Plan.

Source documents that are required to verify the validity and completeness of data entered into the eCRFs must not be obliterated or destroyed and must be retained per the policy for retention of records described below.

To facilitate source data verification, the Investigators and institutions must provide the

Sponsor direct access to applicable source documents and reports for trial‑related

monitoring, Sponsor audits, and IRB/EC review. The investigational site must also allow inspection by applicable Health Authorities.

**2.1.3. Use of Computerized Systems**

When clinical observations are entered directly into an investigational site’s computerized medical record system (i.e., in lieu of original hardcopy records), the electronic record can serve as the source document if the system has been validated in accordance with Health Authority requirements pertaining to computerized systems used in clinical research. An acceptable computerized data collection system allows preservation of the original entry of data. If original data are modified, the system should maintain a viewable audit trail that shows the original data as well as the reason for the change, name of the person making the change, and date of the change.

**2.1.4. Safety Biomarker Data**

Adverse event reports will not be derived from safety biomarker data by the Sponsor, and safety biomarker data will not be included in the formal safety analyses for this study. In addition, safety biomarker data will not inform decisions on participant management.

**2.2. RETENTION OF RECORDS**

Records and documents, including signed ICF, pertaining to the conduct of this study must be retained by the Investigator for at least 15 years after study completion or discontinuation of the study, or for the length of time required by relevant national or local Health Authorities, whichever is longer. After that period of time, the documents may be destroyed, subject to local regulations. No records may be destroyed during the retention period without the written approval of the Sponsor. No records may be transferred to another location or party without written notification to the Sponsor.

The Sponsor will retain study data for 25 years after the final study results have been reported or for the length of time required by relevant national or local Health Authorities.

**2.3. STUDY RECORDS**

The Investigator must maintain adequate and accurate records to enable the conduct of the study to be fully reconstructed, including but not limited to the protocol, protocol amendments, ICFs, and documentation of IRB/EC and governmental approval.

Roche shall also submit an Annual Safety Report once a year to the IEC and CAs according to local regulatory requirements and timelines of each country participating in the study.

**2.3.1. Protocol Amendments**

Any substantial protocol amendments will be prepared by the Sponsor. Substantial protocol amendments will be submitted to the IRB/EC and to regulatory authorities in accordance with local regulatory requirements.

Approval must be obtained from the IRB/EC and regulatory authorities (as locally required) before implementation of any changes, except for changes necessary to eliminate an immediate hazard to participants or any non-substantial changes, as defined by regulatory requirements.

**2.3.2. Publication Policy**

The results of this study may be published or presented at scientific meetings. If this is foreseen, the Investigator agrees to submit all manuscripts or abstracts to the Sponsor for approval prior to submission. This allows the Sponsor to protect proprietary information and to provide comments based on information from other studies that may not yet be available to the Investigator.

The Sponsor will comply with the requirements for publication of study results. In accordance with standard editorial and ethical practice, the Sponsor will generally support publication of multicenter trials only in their entirety and not as individual center data. In this case, a coordinating Investigator will be designated by mutual agreement.

Any formal publication of the study in which contribution of Sponsor personnel exceeded that of conventional monitoring will be considered as a joint publication by the Investigator and the appropriate Sponsor personnel.

Authorship will be determined by mutual agreement and in line with International

Committee of Medical Journal Editors authorship requirements.

Any inventions and resulting patents, improvements, and/or know-how originating from the use of data from this study will become and remain the exclusive and unburdened property of the Sponsor, except where agreed otherwise.

**2.3.3. Dissemination of Clinical Study Data**

A clinical study report containing the results of this trial will be made available to anyone who requests a copy.

**2.3.4. Management of Study Quality**

The Sponsor will implement a system to manage the quality of the study, focusing on processes and data that are essential to ensuring subject safety and data integrity. Prior to first subject entry into the study, the Sponsor will identify and evaluate potential risks associated with critical trial processes and data and will implement controls for

the communication, review and reporting of these risks. Details regarding the applied

approach for the study will be provided in the integrated Risk Based Quality

Management Plan.

**2.3.5. Site Inspections**

Site visits will be conducted by the Sponsor or an authorized representative for inspection of study data, participants’ medical records, and eCRFs. The Investigator will permit national and local Health Authorities, Sponsor monitors, representatives, and collaborators, and the IRBs/ECs to inspect facilities and records relevant to this study.

**3. STUDY AND SITE CLOSURE**

The Sponsor (or designee) has the right to close the study site or terminate this study at any time. Reasons for terminating the study may include, but are not limited to, the following:

• The incidence or severity of adverse events in this or other studies indicates a potential health hazard to participants.

• Participant enrollment is unsatisfactory.

The Sponsor will notify the Investigator and Health Authorities if the study is placed on hold, or if the Sponsor decides to discontinue the study or development program.

Study sites will be closed upon study completion. A study site is considered closed when all required documents and study supplies have been collected and a study-site closure visit has been performed.

The Investigator may initiate study-site closure at any time, provided there is reasonable cause and sufficient notice is given in advance of the intended termination.

The study will be closed in case of approval withdrawal for the study conduct either by the EC or the HA.

The study will be closed in case the maximum sum insured cannot be adapted according to § 40 Abs. 1 S. 3 Nr. 8, Abs. 3 S. 2 AMG.

Reasons for the early closure of a study site by the Sponsor or Investigator may include but are not limited to:

• Failure of the Investigator to comply with the protocol, the requirements of the

IRB/IEC or local Health Authorities, the Sponsor's procedures, or GCP guidelines.

• Inadequate recruitment of participants by the Investigator.

• Discontinuation of further study treatment development.

**Appendix 2**

**Adverse Events: Definitions and Procedures for Evaluating, Follow-up, and Reporting**

**1. DEFINITION OF ADVERSE EVENTS**

According to the E2A ICH guideline for Good Clinical Practice, an **adverse event** is any untoward medical occurrence in a participant or clinical investigation participant administered a pharmaceutical product and which does not necessarily have to have a causal relationship with this treatment.

An adverse event can therefore be:

• Any unfavorable and unintended sign (including an abnormal laboratory finding), symptom, or disease temporally associated with the use of a medicinal product, whether or not considered related to the medicinal product.

**Events Meeting the AE Definition:**

• Deterioration in a laboratory value (hematology, clinical chemistry, or urinalysis) or other clinical test (e.g., ECG, x-ray) that is associated with symptoms or leads to a

change in study treatment or concomitant treatment or discontinuation from study treatment (see Appendix 3, Section 4).

• Exacerbation of a chronic or intermittent preexisting condition, including either an increase in frequency and/or intensity of the condition.

• New conditions detected or diagnosed after study treatment administration even though it may have been present before the start of the study.

• Adverse events that are related to a protocol-mandated intervention, including those that occur prior to assignment of study treatment (e.g., screening invasive procedures such as biopsies).

• "Lack of efficacy" or "failure of expected pharmacological action" per se will not be reported as an AE or SAE unless the progression is unexpectedly accelerated and not in line with the natural history of the disease. If the “Lack of efficacy” would not require safety reporting such instances will be captured in the efficacy assessments. However, the signs, symptoms, and/or clinical sequelae resulting from lack of efficacy will be reported as AE or SAE if they fulfill the definition of an AE or SAE.

**Events NOT Meeting the AE Definition:**

• Any clinically significant abnormal laboratory findings or other abnormal safety assessments which are associated with the underlying disease, unless judged by the Investigator to be more severe than expected for the participant’s condition.

• The disease/disorder being studied or expected progression, signs, or symptoms of the disease/disorder being studied, unless more severe than expected for the participant’s condition.

• Medical or surgical procedure (e.g., endoscopy, appendectomy): the condition that leads to the procedure is an AE.

• Situations where an untoward medical occurrence did not occur (social and/or convenience admission to a hospital).

• Anticipated day-to-day fluctuations of preexisting disease(s) or condition(s) present or detected at the start of the study that do not worsen.

**2. DEFINITION OF SERIOUS ADVERSE EVENTS**

If an event is not an AE per definition above, then it cannot be a serious adverse event (SAE) even if serious conditions are met (e.g., hospitalization for signs/symptoms of the disease under study, death due to progression of disease).

A serious adverse event is defined as any untoward medical occurrence that at any dose:

~~•~~ **Results in death.**

• **Is life-threatening.**

The term "life-threatening" in the definition of "serious" refers to an event in which the participant was at risk of death at the time of the event; it does not refer to an event which hypothetically might have caused death if it was more severe.

• **Requires inpatient hospitalization or prolongation of existing hospitalization**

(see Appendix 3).

In general, hospitalization signifies that the participant has been detained (usually involving at least an overnight stay) at the hospital or emergency ward for observation and/or treatment that would not have been appropriate in the physician’s office or outpatient setting. Complications that occur during

hospitalization are AEs. If a complication prolongs hospitalization or fulfills any other serious criteria, the event is serious. When in doubt as to whether “hospitalization” occurred or was necessary, the AE should be considered serious.

Hospitalization for elective treatment of a preexisting condition that did not worsen from baseline is not considered an AE.

• **Results in persistent or significant disability/incapacity**

Disability means substantial disruption of the participant’s ability to conduct normal life functions.

This definition is not intended to include experiences of relatively minor medical significance such as uncomplicated headache, nausea, vomiting, diarrhea, influenza, and accidental trauma (e.g., sprained ankle) which may interfere with or prevent everyday life functions but do not constitute a substantial disruption.

• **Is a congenital anomaly/birth defect**.

• **Other significant events**:

Medical or scientific judgment should be exercised in deciding whether SAE reporting is appropriate in other situations such as important medical events that may not be immediately life-threatening or result in death or hospitalization but may jeopardize the participant or may require medical or surgical intervention to prevent one of the other outcomes listed in the above definition. These events should usually be considered serious.

Examples of such events include invasive or malignant cancers, intensive treatment in an emergency room or at home for allergic bronchospasm, blood dyscrasias or convulsions that do not result in hospitalization, or development of drug dependency or drug abuse.

**3. RECORDING OF ADVERSE EVENT AND/OR SERIOUS ADVERSE EVENT**

When an AE/SAE occurs, it is the responsibility of the Investigator to review all documentation (e.g., hospital progress notes, laboratory reports, and diagnostics reports) related to the event.

The Investigator will then record all relevant AE/SAE information in the CRF.

It is **not** acceptable for the Investigator to send photocopies of the participant’s medical records to Medical Monitor in lieu of completion of the eCRF.

There may be instances when copies of medical records for certain cases are requested by Sponsor or its delegate. In this case, all participant identifiers, with the exception of the participant number, will be redacted on the copies of the medical records before submission to Sponsor or its delegate.

The Investigator will attempt to establish a diagnosis of the event based on signs, symptoms, and/or other clinical information. Whenever possible, the diagnosis (not the individual signs/symptoms) will be documented as the AE/SAE.

**3.1. ASSESSMENT OF SEVERITY**

The terms “severe” and “serious” are not synonymous. Severity refers to the intensity of an adverse event rated as mild, moderate, or severe, or according to a predefined grading criteria (e.g., National Cancer Institute Common Terminology Criteria for Adverse Events [NCI CTCAE]); the event itself may be of relatively minor medical significance (such as severe headache without any further findings).

Severity and seriousness need to be independently assessed for each adverse event recorded on the eCRF.

Serious adverse events are required to be reported by the Investigator to the Sponsor immediately (i.e., without undue delay).

The adverse event severity grading scale for the NCI CTCAE (v5.0) will be used for assessing adverse event severity. Table 1 will be used for assessing severity for adverse events that are not specifically listed in the NCI CTCAE.

**Table 1 Adverse Event Severity Grading Scale**

**Grade Severity**

1 Mild; asymptomatic or mild symptoms; clinical or diagnostic observations only; or intervention not indicated

2 Moderate; minimal, local, or non-invasive intervention indicated; or limiting age-appropriate instrumental activities of daily livinga

3 Severe or medically significant, but not immediately life-threatening; hospitalization or prolongation of hospitalization indicated; disabling; or limiting self-care activities of daily livingb,c

4 Life-threatening consequences or urgent intervention indicatedd

5 Death related to adverse eventd

NCI CTCAE = National Cancer Institute Common Terminology Criteria for Adverse Events. Note: Based on the NCI CTCAE (v5.0), which can be found at:

https://ctep.cancer.gov/protocolDevelopment/electronic_applications/docs/CTCAE_v5_Quick_

Reference_8.5x11.pdf

a Instrumental activities of daily living refer to preparing meals, shopping for groceries or clothes, using the telephone, managing money, etc.

b Examples of self-care activities of daily living include bathing, dressing and undressing, feeding one's self, using the toilet, and taking medications, as performed by patients who are not bedridden.

c If an event is assessed as a "significant medical event," it must be reported as a serious adverse event (see Section 6 of this Appendix for reporting instructions), per the definition of serious adverse event in Section 2.

d Grade 4 and 5 events must be reported as serious adverse events (see Section 6 for reporting instructions), per the definition of serious adverse event in Section 2. Grade 4 laboratory abnormalities would only be reported as SAEs if these meets one or more of the conditions outlined in Section 2 (Definition of Serious Adverse Events) of Appendix 2.

**3.2. ASSESSMENT OF CAUSALITY**

Investigators should use their knowledge of the participant, the circumstances surrounding the event, and an evaluation of any potential alternative causes to determine whether an adverse event is considered to be related to the study treatment, indicating "yes" or "no" accordingly. The following guidance should be taken into consideration:

• Temporal relationship of event onset to the initiation of study treatment.

• Known association of the event with the study treatment or with similar treatments.

• Known association of the event with the disease under study.

• Presence of risk factors in the participant or use of concomitant medications known to increase the occurrence of the event.

• Presence of non-treatment-related factors that are known to be associated with the occurrence of the event.

For participant receiving combination therapy, causality will be assessed individually for each protocol-mandated therapy.

**4. FOLLOW-UP OF AES AND SAES**

The Investigator is obligated to perform or arrange for the conduct of supplemental measurements and/or evaluations as medically indicated or as requested by the

Sponsor or its delegate to elucidate the nature and/or causality of the AE or SAE as fully as possible. This may include additional laboratory tests or investigations, histopathological examinations, or consultation with other health care professionals.

If a participant dies during participation in the study or during a recognized follow-up period, when possible the Investigator will provide the Sponsor or its delegate with a copy of any post-mortem findings including histopathology.

New or updated information will be recorded in the originally completed eCRF.

The Investigator will submit any updated SAE data to the Sponsor *immediately (i.e., without undue delay)*.

**5. IMMEDIATE REPORTING REQUIREMENTS FROM INVESTIGATOR TO SPONSOR**

Certain events require immediate reporting to allow the Sponsor to take appropriate measures to address potential new risks in a clinical trial. The Investigator must report such events to the Sponsor immediately *(i.e., without undue delay)*. The following is a list of events that the Investigator must report to the Sponsor *immediately (i.e., without undue delay)*, regardless of relationship to study treatment:

• Serious adverse events

• Non-serious adverse events of special interest (NSAESI)

• Pregnancies (see Section 8.2.5)

The Investigator must report new significant follow-up information for these events to the Sponsor immediately *(i.e., without undue delay)*. New significant information includes the following:

• New signs or symptoms or a change in the diagnosis.

• Significant new diagnostic test results.

• Change in causality based on new information.

• Change in the event’s outcome, including recovery.

• Additional narrative information on the clinical course of the event.

Investigators must also comply with local requirements for reporting serious adverse events to the local Health Authority and IRB/EC.

**5.1 REPORTING REQUIREMENTS OF SERIOUS ADVERSE EVENTS, AND NON-SERIOUS ADVERSE EVENTS OF SPECIAL INTEREST**

**Events that Occur prior to Study Treatment Initiation**

After informed consent has been obtained but prior to initiation of study treatment, only serious adverse events caused by a protocol-mandated intervention should be reported. The Clinical Trial Adverse Event/Special Situations Form provided to Investigators should be completed and submitted to the Serious Adverse Event Responsible immediately *(i.e., without undue delay)*.

**Events that Occur after Study Treatment Initiation**

For reports of serious adverse events and NSAESI (Section 8.2.6) that occur after initiation of study treatment (Section 8.2.1), Investigators should record all case details that can be gathered immediately *(i.e., without undue delay)* on the appropriate Adverse Event of Special Interest/Serious Adverse Event eCRF form and submit the report via the electronic data capture (EDC) system. A report will be generated and sent to the Sponsor’s Safety Risk Management department.

In the event that the EDC system is unavailable, the Clinical Trial Adverse Event/Special Situations Form provided to Investigators should be completed and submitted to the Serious Adverse Event Responsible immediately *(i.e., without undue delay).*

Once the EDC system is available, all information will need to be entered and submitted via the EDC system.

**Reporting of Post-Study Adverse Events and Serious Adverse Events**

If the Investigator becomes aware of any other serious adverse event occurring after the end of the AE reporting period, if the event is believed to be related to prior study treatment the event should be reported directly to the Sponsor or its designee, either by faxing or by scanning and emailing the SAE Reporting Form using the fax number or email address provided to Investigators.

**5.2 REPORTING REQUIREMENTS FOR CASES OF OVERDOSE, MEDICATION ERROR, DRUG ABUSE, OR DRUG MISUSE**

Overdose (accidental or intentional), medication error, drug abuse, and drug misuse

(hereafter collectively referred to as "special situations"), are defined as follows:

• Accidental overdose: accidental administration of a drug in a quantity that is higher than the assigned dose

• Intentional overdose: intentional administration of a drug in a quantity that is higher than the assigned dose

• Medication error: accidental deviation in the administration of a drug

In some cases, a medication error may be intercepted prior to administration of the drug.

• Drug abuse: intentional excessive use of a drug that may lead to addiction or dependence, physical harm, and/or psychological harm}

• Drug misuse: intentional deviation in the administration of a drug that does not qualify as drug abuse

In cases where drug is to be self-administered by the participant, drug misuse could involve the drug being administered to someone other than the participant.

Special situations are not in themselves adverse events, but may result in adverse events. Each adverse event associated with a special situation should be recorded separately on the Adverse Event eCRF. If the associated adverse event fulfills seriousness criteria, the event should be reported to the Sponsor immediately *(i.e., without undue delay)*. For RO7486967/placebo, adverse events associated with special situations should be recorded as described below for each situation:

• Accidental overdose: Enter the adverse event term. Check the "Accidental overdose" and "Medication error" boxes.

• Intentional overdose: Enter the adverse event term. Check the "Intentional overdose" box. If drug abuse is suspected, check the "Drug abuse" box. If drug abuse is not suspected, check the "Drug misuse" box.

• Medication error that does not qualify as an overdose: Enter the adverse event term.

Check the "Medication error" box.

• Medication error that qualifies as an overdose: Enter the adverse event term. Check the "Accidental overdose" and "Medication error" boxes.

• Drug abuse that does not qualify as an overdose: Enter the adverse event term.

Check the "Drug abuse" box.

• Drug abuse that qualifies as an overdose: Enter the adverse event term. Check the

"Intentional overdose" and "Drug abuse" boxes.

• Drug misuse that does not qualify as an overdose: Enter the adverse event term.

Check the "Drug misuse" box.

• Drug misuse that qualifies as an overdose: Enter the adverse event term. Check the

"Intentional overdose" and "Drug misuse" boxes.

In addition, all special situations associated with RO7486967/placebo, regardless of whether they result in an adverse event, should be recorded on the Adverse Event eCRF as described below:

• Accidental overdose: Enter the drug name and "accidental overdose" as the event term. Check the "Accidental overdose" and "Medication error" boxes.

• Intentional overdose: Enter the drug name and "intentional overdose" as the event term. Check the "Intentional overdose" box. If drug abuse is suspected, check the "Drug abuse" box. If drug abuse is not suspected, check the "Drug misuse" box.

• Medication error that does not qualify as an overdose: Enter the name of the drug administered and a description of the error (e.g., wrong dose administered, wrong dosing schedule, incorrect route of administration, wrong drug, expired drug administered) as the event term. Check the "Medication error" box.

• Medication error that qualifies as an overdose: Enter the drug name and "accidental overdose" as the event term. Check the "Accidental overdose" and "Medication error" boxes. Enter a description of the error in the additional case details.

• Intercepted medication error: Enter the drug name and "intercepted medication

error" as the event term. Check the "Medication error" box. Enter a description of the error in the additional case details.

• Drug abuse that does not qualify as an overdose: Enter the drug name and "drug abuse" as the event term. Check the "Drug abuse" box.

• Drug abuse that qualifies as an overdose: Enter the drug name and "intentional overdose" as the event term. Check the "Intentional overdose" and "Drug abuse" boxes.

• Drug misuse that does not qualify as an overdose: Enter the drug name and "drug misuse" as the event term. Check the "Drug misuse" box.

• Drug misuse that qualifies as an overdose: Enter the drug name and "intentional overdose" as the event term. Check the "Intentional overdose" and "Drug misuse" boxes.

• Drug administered to someone other than the participant: Enter the drug name and "patient supplied drug to third party" as the event term. Check the "Drug misuse" box.

As an example, an accidental overdose that resulted in a headache would require the completion of two Adverse Event eCRF pages, one to report the accidental overdose and one to report the headache. The "Accidental overdose" and "Medication error" boxes would need to be checked on both eCRF pages.

**6. EXPEDITED REPORTING TO HEALTH AUTHORITIES, INVESTIGATORS, INSTITUTIONAL REVIEW BOARDS, AND ETHICS COMMITTEES**

The Sponsor will promptly evaluate all serious adverse events and NSAESI against cumulative product experience to identify and expeditiously communicate possible new

safety findings to Investigators, IRBs, ECs, and applicable Health Authorities based on applicable legislation.

To determine reporting requirements for single adverse event cases, the Sponsor will assess the expectedness of these events through use of the reference safety

information in the document listed below:

| Drug | Document |
| --- | --- |
| RO7486967 | RO7486967 Investigator Brochure |

The Sponsor will compare the severity of each event and the cumulative event frequency reported for the study with the severity and frequency reported in the applicable reference document.

Reporting requirements will also be based on the Investigator's assessment of causality and seriousness, with allowance for upgrading by the Sponsor as needed.

**Appendix 3**

**Procedures for Recording Adverse Events**

Investigators should use correct medical terminology/concepts when recording adverse events on the Adverse Event eCRF. Avoid colloquialisms and abbreviations.

Only one adverse event term should be recorded in the event field on the Adverse Event eCRF.

**1. DIAGNOSIS VERSUS SIGNS AND SYMPTOMS**

**1.2. OTHER ADVERSE EVENTS**

A diagnosis (if known) should be recorded on the Adverse Event eCRF rather than individual signs and symptoms (e.g., record only liver failure or hepatitis rather than jaundice, asterixis, and elevated transaminases). However, if a constellation of signs and/or symptoms cannot be medically characterized as a single diagnosis or syndrome at the time of reporting, each individual event should be recorded on the Adverse Event eCRF. If a diagnosis is subsequently established, all previously reported adverse events based on signs and symptoms should be nullified and replaced by one adverse event report based on the single diagnosis, with a starting date that corresponds to the starting date of the first symptom of the eventual diagnosis.

**2. ADVERSE EVENTS OCCURRING SECONDARY TO OTHER EVENTS**

In general, adverse events occurring secondary to other events (e.g., cascade events or clinical sequelae) should be identified by their primary cause, with the exception of severe or serious secondary events. However, medically significant adverse events occurring secondary to an initiating event that are separated in time should be recorded as independent events on the Adverse Event eCRF. For example:

• If vomiting results in mild dehydration with no additional treatment in a healthy adult, only vomiting should be reported on the eCRF.

• If vomiting results in severe dehydration, both events should be reported separately on the eCRF.

• If a severe gastrointestinal hemorrhage leads to renal failure, both events should be reported separately on the eCRF.

• If dizziness leads to a fall and subsequent fracture, all three events should be reported separately on the eCRF.

All adverse events should be recorded separately on the Adverse Event eCRF if it is unclear as to whether the events are associated.

**3. PERSISTENT OR RECURRENT ADVERSE EVENTS**

A persistent adverse event is one that extends continuously, without resolution, between participant-evaluation timepoints. Such events should only be recorded once on the Adverse Event eCRF. The initial severity of the event should be recorded, and the severity should be updated to reflect the most extreme severity any time the event worsens. If the event becomes serious, the Adverse Event eCRF should be updated to reflect this.

A recurrent adverse event is one that resolves between participant-evaluation timepoints and subsequently recurs. Each recurrence of an adverse event should be recorded separately on the Adverse Event eCRF.

**4. ABNORMAL LABORATORY VALUES**

Not every laboratory abnormality qualifies as an adverse event. A laboratory test result should be reported as an adverse event if it meets any of the following criteria:

• Accompanied by clinical symptoms.

• Results in a change in study treatment (e.g., dosage modification, treatment interruption, or treatment discontinuation).

• Results in a medical intervention (e.g., potassium supplementation for hypokalemia)

or a change in concomitant therapy.

• Clinically significant in the Investigator’s judgment.

It is the Investigator’s responsibility to review all laboratory findings. Medical and scientific judgment should be exercised in deciding whether an isolated laboratory abnormality should be classified as an adverse event.

If a clinically significant laboratory abnormality is a sign of a disease or syndrome (e.g., ALP and bilirubin 5 times the upper limit of normal [ULN] associated with cholecystitis), only the diagnosis (i.e., cholecystitis) should be recorded on the Adverse Event eCRF.

If a clinically significant laboratory abnormality is not a sign of a disease or syndrome, the abnormality itself should be recorded on the Adverse Event eCRF, along with a descriptor indicating if the test result is above or below the normal range (e.g., "elevated potassium", as opposed to "abnormal potassium"). If the laboratory abnormality can be characterized by a precise clinical term per standard definitions, the clinical term should be recorded as the adverse event. For example, an elevated serum potassium level of

7.0 mEq/L should be recorded as “hyperkalemia.”

Observations of the same clinically significant laboratory abnormality from visit to visit should not be repeatedly recorded on the Adverse Event eCRF, unless the etiology

changes. The initial severity of the event should be recorded, and the severity or seriousness should be updated any time the event worsens.

**5. ABNORMAL VITAL SIGN VALUES**

Not every vital sign abnormality qualifies as an adverse event. A vital sign result should be reported as an adverse event if it meets any of the following criteria:

• Accompanied by clinical symptoms.

• Results in a change in study treatment (e.g., dosage modification, treatment interruption, or treatment discontinuation).

• Results in a medical intervention or a change in concomitant therapy.

• Clinically significant in the Investigator’s judgment.

It is the Investigator’s responsibility to review all vital sign findings. Medical and scientific judgment should be exercised in deciding whether an isolated vital sign abnormality should be classified as an adverse event.

If a clinically significant vital sign abnormality is a sign of a disease or syndrome

(e.g., high blood pressure), only the diagnosis (i.e., hypertension) should be recorded on the Adverse Event eCRF.

Observations of the same clinically significant vital sign abnormality from visit to visit should not be repeatedly recorded on the Adverse Event eCRF, unless the etiology changes. The initial severity of the event should be recorded, and the severity or seriousness should be updated any time the event worsens.

**6. ABNORMAL LIVER FUNCTION TESTS**

The finding of an elevated ALT or AST (> 3 × ULN) in combination with either an elevated total bilirubin (> 2 × ULN) or clinical jaundice in the absence of cholestasis or other

causes of hyperbilirubinemia is considered to be an indicator of severe liver injury. Therefore, Investigators must report as an adverse event the occurrence of either of the following:

• Treatment-emergent ALT or AST > 3 × ULN in combination with total bilirubin > 2 × ULN.

• Treatment-emergent ALT or AST > 3 × ULN in combination with clinical jaundice. The most appropriate diagnosis or (if a diagnosis cannot be established) the abnormal

laboratory values should be recorded on the Adverse Event eCRF (see Appendix 2) and reported to the Sponsor immediately *(i.e., without undue delay)*, either as a serious adverse event or a non-serious adverse event of special interest (see Section 8.2.6).

**7. DEATHS**

All deaths that occur during the protocol-specified adverse event reporting period (see Section 5 of Appendix 2), regardless of relationship to study treatment, must be recorded on the Adverse Event eCRF and immediately reported to the Sponsor. This includes death attributed to progression of ulcerative colitis.

Death should be considered an outcome and not a distinct event. The event or condition that caused or contributed to the fatal outcome should be recorded as the single medical concept on the Adverse Event eCRF. Generally, only one such event should be

reported. If the cause of death is unknown and cannot be ascertained at the time of reporting, “unexplained death” should be recorded on the Adverse Event eCRF. If the cause of death later becomes available (e.g., after autopsy), “unexplained death” should be replaced by the established cause of death. The term "sudden death" should not be used unless combined with the presumed cause of death (e.g., "sudden cardiac death").

**8. PREEXISTING MEDICAL CONDITIONS**

A preexisting medical condition is one that is present at the screening visit for this study. Such conditions should be recorded on the General Medical History and Baseline Conditions eCRF.

A preexisting medical condition should be recorded as an adverse event only if the frequency, severity, or character of the condition worsens during the study. When recording such events on the Adverse Event eCRF, it is important to convey the concept that the preexisting condition has changed by including applicable descriptors

(e.g., “more frequent headaches”).

**9. HOSPITALIZATION OR PROLONGED HOSPITALIZATION**

Any adverse event that results in hospitalization or prolonged hospitalization should be documented and reported as a serious adverse event (per the definition of serious adverse event in Appendix 2), except as outlined below.

An event that leads to hospitalization under the following circumstances should not be reported as an adverse event or a serious adverse event:

• Hospitalization for respite care.

• Planned hospitalization required by the protocol (e.g., for study treatment administration or insertion of access device for study treatment administration).

• Hospitalization for a preexisting condition, provided that all of the following criteria are met:

The hospitalization was planned prior to the study or was scheduled during the study when elective surgery became necessary because of the expected normal progression of the disease.

The participant has not suffered an adverse event.

An event that leads to hospitalization under the following circumstances is not considered to be a serious adverse event, but should be reported as an adverse event instead:

• Hospitalization for an adverse event that would ordinarily have been treated in an outpatient setting had an outpatient clinic been available.

**Appendix 4**

**Clinical Laboratory Tests**

The tests detailed in Table 1 will be performed by the local laboratory except exploratory safety biomarkers. If the local laboratory results are used, the results must be captured

in source documentation and entered into the eCRF.

The local laboratory results must be captured in source documentation and entered into the eCRF.

Protocol-specific requirements for inclusion or exclusion of participants are detailed in

Sections 5.1 and 5.2, respectively, of the protocol.

Additional tests may be performed at any time during the study as determined necessary by the Investigator or required by local regulations.

**Table 1 Protocol-Required Safety Laboratory Assessments**

**Laboratory Assessments Parameters**

Hematology

Clinical chemistry

Coagulation Tuberculosis SARS-CoV-2

Viral serology and PCR

Lipids Hormone Pregnancy test

Urinalysis

Other screening tests

Safety exploratory biomarkers

• Leucocytes, erythrocytes, hemoglobin, hematocrit, platelets, differential count (neutrophils, eosinophils, basophils, monocytes, lymphocytes).

• Sodium, potassium, chloride, bicarbonate, glucose (fasting), urea, creatinine, creatinine clearance at screening only, protein, albumin, phosphate, calcium, total and direct bilirubin, ALP, ALT, AST,

GGT, urate, LDH, cardiac Troponin I, high sensitivity

CRP

• INR, aPTT, PT.

• Quantiferon ®Gold test

• PCR or antigen test, as applicable

• HIV (specific tests HIV-1 antibody, HIV-1/2 antibody, HIV-2 antibody), hepatitis B surface antigen (HBsAg), total hepatitis B core antibody

(HBcAb), HBV PCR (≤ 20IU/mL) in case positive for anti-HBc to exclude replicating disease, hepatitis C virus (HCV) PCR.

• Cholesterol, LDL cholesterol, HDL cholesterol, triglycerides.

• Estradiol, follicle-stimulating hormone (FSH), luteinizing hormone (LH) in females

~~•~~ All women of childbearing potential (including those who have had a tubal occlusion/ligation) will have a blood pregnancy test at screening. Urine pregnancy tests will be performed at specified subsequent visits. If a urine pregnancy test is positive, it must be confirmed by a blood pregnancy test.

• Specific gravity

• Dipstick: pH, glucose, protein, blood, ketones, bilirubin, urobilinogen, nitrite, leukocyte esterase

• If there is a clinically significant positive result, urine will be sent to the laboratory for microscopy and culture. If there is an explanation for the positive dipstick results (e.g., menses), it should be recorded and there is no need to perform microscopy and culture.

• Microscopic examination (RBCs, WBCs, casts, crystals, epithelial cells, bacteria), if blood or protein is abnormal.

• Alcohol and drug screen (to include at minimum: amphetamines, barbiturates, cocaine, opiates, cannabinoids and benzodiazepines).

• Glutamate Dehydrogenase (GLDH), osteopontin, Macrophage Colony Stimulating Factor Receptor 1 (M-CSFR-1), Cytokeratin-18 (CK-18) (including fragmented CK18 and full length CK18).

The results of each test will be entered into the eCRF.

Investigators must document their review of each laboratory safety report.

Laboratory/analyte results that could unblind the study will not be reported to investigative sites or other blinded personnel until the study has been unblinded.

**Additional Statistical Considerations for Clinical Laboratory Data**

• Standard Reference Ranges and Transformation of Data

Potential analysis considerations for analyzing laboratory data includes the use of standard reference ranges and potential transformation of data for specific laboratory tests.

In this scenario, Roche standard reference ranges, rather than the reference ranges of the Investigator, can be used for specific parameters. For these parameters, the measured laboratory test result will be assessed directly using the Roche standard reference range. Certain laboratory parameters will be transformed to Roche’s standard reference ranges.

A transformation will be performed on certain laboratory tests that lack sufficiently common procedures and have a wide range of Investigator ranges, e.g., enzyme tests that include AST, ALT, and ALP and total bilirubin. Since the standard reference ranges for these parameters have a lower limit of zero, only the upper limits of the ranges will be used in transforming the data.

• Definition of Laboratory Abnormalities

For all laboratory parameters included in the analysis described above, there exists a Roche predefined standard reference range. Laboratory values falling outside this standard reference range will be labeled “H” for high or “L” for low in participant statistical outputs of laboratory data.

In addition to the standard reference range, a marked reference range has been predefined by Roche for these laboratory parameters. The marked reference range is broader than the standard reference range. Values falling outside the marked reference range that also represent a defined change from baseline will be considered marked laboratory abnormalities (i.e., potentially clinically relevant). If a baseline value is not available for a participant, the midpoint of the standard reference range will be used as the participant’s baseline value for the purposes of determining marked laboratory abnormalities. Marked laboratory abnormalities will be labeled in the participant listings as “HH” for very high or “LL” for very low.

**Appendix 5**

**Contraceptive and Barrier Guidance**

**1. DEFINITIONS**

• **Woman of Childbearing Potential (WOCBP)**

A woman is considered fertile following menarche and until becoming post-menopausal unless permanently sterile. The definition of childbearing potential may be adapted for alignment with local guidelines or requirements.

• **Women in the following categories are considered to be Woman of Non- Childbearing Potential (WONCBP)**

a) Pre-menarchal

b) Pre-menopausal with one of the following:

**–** Documented hysterectomy.

**–** Documented bilateral salpingectomy.

**–** Documented bilateral oophorectomy.

Note: Documentation can come from the site personnel’s: review of participant’s medical records, medical examination, or medical history interview.

c) Post-menopausal

**–** A post-menopausal state is defined as no menses for ≥ 12 months without an alternative medical cause other than menopause. A high follicle-stimulating hormone (FSH) level in the post-menopausal range may be used to confirm a post-menopausal state in women not using hormonal contraception or hormonal

replacement therapy (HRT). However, in the absence of 12 months of amenorrhea, a single FSH measurement is insufficient.

**–** Female participants on HRT and whose menopausal status is in doubt will be required to use one of the non-hormonal highly effective contraception methods if they wish to continue their HRT during the study.

**–** Only discontinue HRT to allow confirmation of post-menopausal status before study enrollment.

**2. CONTRACEPTION GUIDANCE**

• **Female Participants**

Female participants of childbearing potential are eligible to participate if they agree to use highly effective method of contraception consistently and correctly as described in the inclusion criteria (see Section 5.1) and in Table 1 below.

Per ICH M3(R2), highly effective methods of birth control are defined as those, alone or in combination, that result in a low failure rate (i.e. less than 1% per year) when used consistently and correctly as described in Table 1 below.

**Table 1 Contraceptive Methods by Effectiveness**

| **Highly Effective Contraceptive Methods That Are User-Dependenta**  (Failure rate of < 1% per year when used consistently and correctly) |
| --- |
| • Combined (estrogen- and progestogen-containing) hormonal contraception associated with inhibition of ovulation:  o Oral  o Intravaginal  o Transdermal  • Progestogen-only hormonal contraception associated with inhibition of ovulation:  o Oral  o Injectable |
| **Highly Effective Contraceptive Methods That Are User-Independent**  (Failure rate of < 1% per year) |
| • Implantable progestogen-only hormonal contraception associated with inhibition of ovulationa  • Intrauterine device (IUD)  • Intrauterine hormone-releasing system (IUS)  • Bilateral tubal occlusion/ ligation  **Azoospermic partner (vasectomized or due to medical cause)**  A vasectomized partner is a highly effective contraception method provided that the partner is the sole male sexual partner of the WOCBP and the absence of sperm has been confirmed. If not, an additional highly effective method of contraception should be used.  **Sexual abstinence**  Sexual abstinence is considered a highly effective method only if defined as refraining from heterosexual intercourse during the entire period of risk associated with the study treatment. The reliability of sexual abstinence needs to be evaluated in relation to the duration of the study and the preferred and usual lifestyle of the participant. |

**Effective/Acceptable Contraceptive Methods** (Failure rate of > 1% per year when used

consistently and correctly)

• Progestogen-only oral hormonal contraception, where inhibition of ovulation is not the primary mode of action

• Male or female condom with or without spermicide b

• Cap, diaphragm or sponge with spermicide b

a) Hormonal contraception may be susceptible to interaction with the IMP, which may reduce the efficacy of the contraception method.

Typical use failure rates may differ from those when used consistently and correctly. Use should be consistent with local regulations regarding the use of contraceptive methods for participants participating in clinical studies.

b) A combination of male condom with either cap, diaphragm, or sponge with spermicide (double barrier methods) are also considered acceptable, but not highly effective, birth control methods (i.e., when the risk of teratogenicity and genotoxicity is unlikely).

**3. PREGNANCY TESTING**

For WOCBP enrolled in the study, blood sample and urine pregnancy tests will be performed according to Schedule of Activity tables (see Section 1.3). If a urine pregnancy test is positive, it must be confirmed by a blood pregnancy test.

Pregnancy testing will be performed whenever a menstrual cycle is missed or when pregnancy is otherwise suspected and according to local practice.

**4. COLLECTION OF PREGNANCY INFORMATION**

• **Female participants who become pregnant**

The Investigator will collect pregnancy information on any female participant, who becomes pregnant while participating in this study (see Section 8.2.5 Pregnancy). Information will be recorded on the Clinical Trial Pregnancy Reporting Form and submitted to the Sponsor within 24 hours of learning of a participant's pregnancy. The participant will be followed to determine the outcome of the pregnancy. The Investigator will collect follow-up information on the participant and the neonate, which will be forwarded to the Sponsor. Monitoring of the participant should continue until conclusion of the pregnancy. Any termination of pregnancy will be reported, regardless of fetal status (presence or absence of anomalies) or indication for procedure.

While pregnancy itself is not considered an AE or SAE, and should not be recorded on the AE eCRF, any pregnancy complication will be reported as an AE or SAE. A spontaneous abortion is always considered an SAE and will be reported as such. Any post-study pregnancy related SAE considered reasonably related to the study treatment by the Investigator will be reported to the Sponsor as described in Appendix 2. While the

Investigator is not obligated to actively seek this information in former study participants, he/she may learn of an SAE through spontaneous reporting.

Any female participant who becomes pregnant while participating in the study will discontinue study treatment and be withdrawn from the study.

Additionally, attempts should be made to collect and report infant health information. When permitted by the site, an Authorization for the Use and Disclosure of Infant Health Information would need to be signed by one or both parents (as per local regulations) to allow for follow-up on the infant. If the authorization has been signed, the infant's health status at birth should be recorded on the Clinical Trial Pregnancy Reporting Form. In addition, the Sponsor may collect follow-up information on the infant's health status at

6 and 12 months after birth.

**5 ABORTIONS**

Any spontaneous abortion should be classified as a serious adverse event (as the Sponsor considers spontaneous abortions to be medically significant events), recorded on the Adverse Event eCRF, and reported to the Sponsor immediately *(i.e., without undue delay*; see Section 5 of Appendix 2).

Any induced abortion due to maternal toxicity and/or embryofetal toxicity should also be classified as serious adverse event, recorded on the Adverse Event eCRF, and reported to the Sponsor immediately *(i.e., without undue delay*; see Section 5 of Appendix 2).

Elective or therapeutic abortion not associated with an underlying maternal or embryofetal toxicity (e.g., induced abortion for personal reasons) does not require expedited reporting but should be reported as outcome of pregnancy on the Clinical Trial Pregnancy Reporting Form.

**6 CONGENITAL ANOMALIES/BIRTH DEFECTS**

Any congenital anomaly/birth defect in a child born to a female participant exposed to study treatment should be classified as a serious adverse event, recorded on the Adverse Event eCRF, and reported to the Sponsor immediately *(i.e., without undue delay)*.
